# Continuous assessment in medical education: Exploring students’ views on the progress test

**DOI:** 10.1371/journal.pone.0314848

**Published:** 2024-12-19

**Authors:** Helena Landim Gonçalves Cristóvão, Nathalia Bavaresco Gonçalves Cristóvão, Lenina Mariana de Moraes Castellari, André Bavaresco Gonçalves Cristóvão, Emerson Roberto dos Santos, Alex Bertolazzo Quitério, Ryan Emiliano da Silva, Denise Cristina Mós Vaz Oliani, Alba Regina de Abreu Lima, Vânia Maria Sabadoto Brienze, Dorotéia Rossi da Silva Souza, Tatiane Iembo, Thais Santana Gastardelo Bizotto, Júlio César André

**Affiliations:** 1 Center for Studies and Development of Health Education—Faculty of Medicine of São José do Rio Preto (CEDES / FAMERP), São José do Rio Preto, São Paulo, Brazil; 2 University of Santo Amaro (UNISA), São Paulo, Brazil; 3 University Hospital Center Cova da Beira, University of Beira Interior, Covilhã, Portugal; 4 Faculty of Medicine in São José do Rio Preto (FACERES), São José do Rio Preto, São Paulo, Brazil; Faculdade Sao Leopoldo Mandic, BRAZIL

## Abstract

The Progress Test (PT) is a valuable tool for the continuous assessment of medical students’ learning. This quantitative, cross-sectional study aimed to understand the perceptions of 908 medical students (702 from a private and 206 from a public institution) in São Paulo, Brazil, regarding the design and implementation of PT. Analysis included descriptive statistics, Spearman’s correlation, Pearson’s Chi-square test, and Fisher’s exact test. Results revealed a strong positive correlation between academic progression and expected PT performance. Students from both institutions positively perceived PT construction and received adequate information about its importance. Significant differences were observed between institutions regarding classroom discussion of PT questions and content adequacy. The study highlights PT’s strengths and challenges, emphasizing the need for curriculum alignment, individualized feedback, and strategies to enhance student motivation. It contributes to the field of medical education assessment by providing insights for improving PT implementation and effectiveness. Future research directions include exploring stakeholder perspectives, conducting longitudinal studies, and examining PT’s impact across diverse educational contexts.This research underscores the value of student perspectives in developing effective assessment practices, supporting the evolution of competent medical professionals through meaningful educational strategies.

## Introduction

The 1970s was a period when numerous curricular changes began to occur in higher education courses. This educational movement resulted in the creation and introduction of the Progress Test (PT) at the end of that decade [1–3]. Initially developed at the University of Limburg in Maastricht, Netherlands, the PT has since gained global recognition as a valuable tool for continuous assessment of medical students’ learning [4].

The PT’s global adoption has been diverse, with implementations in countries such as the United Kingdom, Canada, Germany, and Brazil, each adapting the test to their specific educational contexts [5,6]. In Brazil, the first PT was applied in 1998, expanding to more than 60 medical school teaching institutions [7,8]. Currently, the PT in Brazil consists of 120 multiple-choice questions, with four response options, which assess students’ knowledge in the seven main areas of medicine: gynecology and obstetrics, surgery, internal medicine, pediatrics, public health, basic sciences, and bioethics [2,9].

From a theoretical perspective, the PT can be understood through the lens of Vygotsky’s concept of zones of proximal development [10]. This approach allows for the systematic design of curricular interfaces that guide students from their current developmental zone to their potential developmental zone [11]. This theoretical framework aligns with the PT’s goal of continuously and systematically monitoring students’ learning processes throughout their medical education [12–15].

While the benefits of PT are widely reported in the literature as positive for the learning process, few published studies explore the perceptions of medical students who take it, especially in the Brazilian context [14,16–18]. Studies from other countries have provided valuable insights. For instance, undergraduate students in the United Kingdom reported that constant learning, intrinsic motivation, understanding, and application of knowledge in clinical scenarios were important for the success of PT. Students in the Netherlands felt that, instead of being oriented towards the "mechanized" reproduction of knowledge, PT is an assessment of meaning [3,19].

However, it is important to note that the reception of new assessment methods can vary across different cultural contexts, reinforcing the socio-interactionist stance that forms a fundamental element of the basic epistemological matrix for understanding developmental zones [20,21]. This cultural variability underscores the need for context-specific research on PT perceptions and effectiveness.

Recent research has highlighted the importance of understanding students’ beliefs and perceptions about assessment methods, including the PT, as these can significantly influence their learning approaches and outcomes [22]. This study, which explored medical students’ perceptions of continuous assessment methods, including the PT, found that students generally viewed such methods positively, perceiving them as beneficial for their learning and professional development.

Considering the originality of this study in the Brazilian context and its potential contributions to the improvement of PT and the advancement of knowledge in the area of educational assessment in medicine [23], and given that students are the main protagonists in the assessments used in institutional programs, it is imperative to evaluate their perceptions and experiences to inform the future development of assessment methods [24].

Thus, this study aims to address the following research questions:

1. How do medical students from two distinct Brazilian institutions perceive the design and implementation of the PT?
2. What are the students’ beliefs about the effectiveness of the PT in assessing their medical knowledge and skills?
3. How do students’ perceptions of the PT align with or differ from those reported in international contexts?

To frame these questions theoretically, we draw upon the conceptual framework of "assessment for learning" [25], which emphasizes the formative role of assessment in guiding and enhancing student learning. This framework allows us to explore how students’ perceptions of the PT align with its intended purpose as a tool for continuous learning and development. By addressing these questions, this study aims to contribute to the growing body of literature on student perceptions of assessment methods in medical education, with a specific focus on the Brazilian context. The findings will provide valuable insights for educators and policymakers involved in the design and implementation of assessment strategies in medical education.

## Materials and methods

### Study design and setting

This study employed a quantitative, cross-sectional design to investigate medical students’ perceptions of the PT at two higher education institutions in São Paulo, Brazil: one public and one private. The choice of these institutions aimed to capture potential differences in perceptions related to the distinct academic contexts and student profiles often associated with public and private institutions in Brazil.

### Participants and sample size

Participants included medical students from both institutions who had completed at least one PT and volunteered to participate in the study. To ensure representativeness, a sample size calculation was performed using a confidence level of 95% and a margin of error of 5%, considering the estimated student populations of each institution. This calculation resulted in a target sample size of 200 students for the public institution and 650 students for the private institution. The difference in sample sizes between the two institutions reflects the variation in their respective student populations.

### Data collection instrument

Data were collected using a self-administered questionnaire specifically developed for this study. The questionnaire comprised three sections:

1. Demographics: This section collected information on participants’ age, gender, year of medical school, and institution type (public or private).
2. Perceptions of PT Construction and Institutional Movements: This section used Likert-type scales to assess students’ opinions on various aspects of the PT, including clarity of questions, relevance to the curriculum, and perceived impact on learning.
3. Access to Commented Answer Key and PT Results: This section used both Likert-type scales and open-ended questions to explore students’ experiences with accessing and utilizing feedback provided through the PT.

Before implementation, the questionnaire underwent a pilot test with a small group of medical students (n = 50) to ensure clarity, comprehensibility, and relevance of the questions. Feedback from the pilot test was used to refine the questionnaire and ensure its suitability for the target population. The complete questionnaire, in both Portuguese (original language) and English, is available in S1 Appendix.

This study was conducted following the ethical guidelines outlined by the Research Ethics Committee (REC), which approved the research protocol (Protocol number: 3.570.977). Participation in the study was voluntary and anonymous. Informed consent was obtained from all participants, who were informed of the study’s objectives, procedures, and their right to withdraw at any time without penalty.

### Data analysis

The data were collected, coded, and tabulated in Excel, and imported into IBM-SPSS Statistics version 28 software (IBM Corporation, NY, USA) for exploratory and comparative analysis between groups. Exploratory data analysis included descriptive statistics for numerical variables and numbers, and proportion for categorical variables [26]. Spearman’s non-parametric correlation analysis was performed to verify the correlation between discrete and ordinal variables (undergraduate semester and questionnaire item).

The results were demonstrated by Spearman’s correlation coefficient, measured using a scale of values from +1 to -1; for a value close to +1, a perfect positive linear correlation is assumed (i.e., the higher the value of one variable, the higher the value of the other variable), and for a coefficient value close to -1, a perfect negative linear correlation is assumed (i.e., the higher the value of one variable, the lower the value of the other). Values close to zero indicated the absence of correlation. The strength of the correlation between two variables was interpreted as follows: r |≤0.25| = absence of correlation; |0.26–0.50| = weak correlation; |0.51–0.75| = moderate correlation, and |>0.75| = strong correlation. The "r" result was found in the sample. On the other hand, the 95% CI (Confidence Interval) showed the r values extrapolated to the population, with 95% confidence [27,28].

The comparison between students from the two HEIs was performed using Pearson’s Chi-square test or Fisher’s exact test [26,27,29]. When statistical significance was found, multiple comparisons (pairwise method) were performed using the z-test for difference in proportions [30], with p-values adjusted by the Bonferroni correction, to verify where the effect of the differences was. The results were expressed in the tables as numbers and percentages, with notations in letters indicating similarities or differences between categories (IBM SPSS Statistics Algorithms, 2020).

## Results

### Sample characteristics

The study encompassed 908 medical students, comprising 702 (65.3% of 1,075 enrolled students) from a private medical school and 206 (46.2% of 446 enrolled students) from a public medical school. Table 1 presents the demographic characteristics of the participants.

**Table 1:** Demographic characteristics of study participants.

Characteristic	Private Institution (n = 702)	Public Institution (n = 206)	Total (n = 908)
Age, n (%)			
17–20 years	215 (30.6)	48 (23.3)	263 (29.0)
21–25 years	350 (49.9)	131 (63.6)	481 (53.0)
26–30 years	108 (15.4)	25 (12.1)	133 (14.6)
31–35 years	16 (2.3)	0 (0.0)	16 (1.8)
36–39 years	4 (0.6)	0 (0.0)	4 (0.4)
≥40 years	9 (1.3)	2 (1.0)	11 (1.2)
Gender, n (%)			
Female	398 (56.7)	112 (54.4)	510 (56.2)
Male	304 (43.3)	94 (45.6)	398 (43.8)
Year of study, n (%)			
1st year	201 (28.6)	59 (28.6)	260 (28.6)
2nd year	115 (16.4)	33 (16.0)	148 (16.3)
3rd year	76 (10.8)	23 (11.2)	99 (10.9)
4th year	52 (7.4)	15 (7.3)	67 (7.4)
5th year	47 (6.7)	14 (6.8)	61 (6.7)
6th year	211 (30.1)	62 (30.1)	273 (30.1)

Source: Authors.

The predominant age group was 21–25 years, representing 350 (49.9%) participants from the private institution and 131 (63.6%) from the public institution. Female students constituted the majority in both institutions, accounting for 56.7% and 54.4% of participants in the private and public schools, respectively. The distribution of students across years of study was similar between the two institutions, with the highest proportion of participants in the first year (28.6%) and the sixth year (30.1%) of medical education.

### Student perception and course progression

Non-parametric analysis revealed a strong positive correlation between the semester of study and students’ perception of the percentage of questions they expected to answer correctly in the Progress Test (PT) at both institutions (Table 2).

**Table 2:** Correlation between year of study and expected correct answers in the PT.

Item	rs (95% CI)	Interpretation
Percentage of questions expected to answer correctly	0.752 (0.713 to 0.786)	Strong positive correlation

### PT adequacy and institutional actions

The majority of students from both institutions strongly agreed with the adequacy of PT construction and institutional actions to promote student adherence. Correlation analysis (Table 3) demonstrated no significant correlation between responses and semester of study, except for the item "Completion time is adequate for the content," which exhibited a weak positive correlation.

**Table 3:** Correlation between year of study and responses regarding PT construction adequacy and institutional actions.

Item	rs (95% CI)	Interpretation
Question statements and alternatives are clear	-0.033 (-0.107 to 0.041)	No correlation
Completion time is adequate for the content	0.153 (0.080 to 0.225)	Weak positive correlation
Received prior information from the institution about test importance	-0.051 (-0.124 to 0.023)	No correlation

Source: Author.

rs = Spearman’s correlation coefficient; IC, Confidence Interval.

### Access to answer key and PT results

The majority of students expressed strong interest in accessing the commented answer key and PT results. Correlation analysis (Table 4) revealed a weak positive correlation between semester of study and interest in accessing the commented answer key.

**Table 4:** Correlation between year of study and responses regarding access to answer key and PT results.

Item	rs (95% CI)	Interpretation
Intends to access the commented answer key	0.186 (0.113 to 0.257)	Weak positive correlation
Intends to access the results	0.106 (0.032 to 0.178)	No significant correlation

Source: Author.

rs = Spearman’s correlation coefficient; IC, Confidence Interval.

### Utilization of PT results by institutions

Comparative analysis of student responses regarding the use of PT results by the institution (Table 5) revealed significant differences between the two schools, particularly for the item "Questions are subsequently discussed in the classroom."

**Table 5:** Comparative analysis of PT results utilization by institutions.

Item	Responses	Private (%)	Public (%)	P-value*
Questions are subsequently discussed in the classroom	Strongly disagree	28.3	62.4	< 0.001
	Strongly agree	15.6	2.9	
The content covered in your institution is adequate for taking the test	Partially agree	32.7	45.6	< 0.001
	Strongly agree	41.9	26.2	

Source: Author.

*Pearson’s Chi-square test.

Correlation analysis (Table 6) demonstrated a weak positive correlation between semester of study and the perception that questions are discussed in the classroom.

**Table 6:** Correlation between year of study and responses regarding the utilization of PT results by the Higher Education Institution (HEI).

Item	rs (95% CI)	Interpretation
Questions are subsequently discussed in the classroom	0.314 (0.244 to 0.381)	Weak positive correlation
Importance of discussing questions in the classroom	0.141 (0.068 to 0.231)	No significant correlation
The content covered in your institution is adequate for taking the test	-0.202 (-0.273 to -0.129)	Weak negative correlation

Source: Author.

rs = Spearman’s correlation coefficient; CI = Confidence Interval.

### Student motivation and use of PT results

Comparative analysis between students from the two institutions (Table 7) revealed significant differences in three of the four items related to motivation and use of PT results.

**Table 7:** Comparative analysis of student motivation and use of PT results.

Item	Responses	Private (%)	Public (%)	P-value*
Motivated to take the test	Strongly agree	51.8	32.5	< 0.001
Importance of taking the test for academic development	Strongly agree	71.8	51.0	< 0.001
Takes into account the evolution of test performance to guide studies	Strongly agree	36.8	22.4	0.001

Source: Author.

*Pearson’s Chi-square test.

Correlation analysis (Table 8) showed no significant correlations between responses and semester of study.

**Table 8:** Correlation between year of study and responses regarding student motivation and use of PT results for academic development.

Item	rs (95% CI)	Interpretation
Motivated to take the test	-0.082 (-0.155 to -0.008)	No significant correlation
Importance of taking the test for academic development	-0.129 (-0.201 to -0.055)	No significant correlation
Takes into account test performance to assess academic development	-0.103 (-0.194 to -0.011)	No significant correlation
Takes into account the evolution of test performance to guide studies	0.127 (0.035 to 0.217)	No significant correlation

Source: Author.

rs = Spearman’s correlation coefficient; CI = Confidence Interval.

## Discussion

The present study aimed to understand the perceptions of medical students from two distinct institutions regarding the design and implementation of the Progress Test (PT). The results obtained showed that the PT offers teachers and institutions the opportunity to review the teaching-learning processes related to their course, knowledge area, and competency maturation depending on the year of graduation, in addition to helping identify strengths and weaknesses in the curricular structure, which is useful for finding possible solutions to the obstacles presented and for improving teaching tools [12,14,31–33].

From the perspective of Vygotsky’s theory, the PT represents an opportunity for institutions to monitor the perimeter of their current zones of development through scaffolding that observes the progression of difficulty and skillfully integrates the focal knowledge and competencies of each learning cycle, as well as evaluating the idiosyncrasies of the seven main areas observed [34]. This approach aligns with the longitudinal nature of the PT, allowing for a more holistic view of student development throughout the course [35].

The results of the present study corroborate the effectiveness and reliability of the PT as an instrument for assessing learning during undergraduate training in health professions [36]. Given its broad scope, PTs can reveal curricular deficiencies in student preparation, knowledge gaps, or areas of low performance that need attention, offering realistic and contextualized content, oriented towards individual monitoring [16,37]. This finding is reinforced by the "strong positive correlation" found in the present research regarding students’ perception, from both HEIs, in the sense of answering a higher percentage of PT questions as the semester progresses, since their academic development can generate confidence according to the level of maturity achieved.

Cultural differences play a significant role in shaping students’ perceptions of the PT. A study by Verhoeven et al. [38] found that students from diverse cultural backgrounds may interpret and approach the PT differently, influenced by their educational experiences and cultural values. This cultural dimension adds complexity to the implementation and interpretation of PT results across different institutions and regions.

Considering the need to adapt the PT to the various local cultural and academic needs [39,40], the participants of this study had a positive perception that the two HEIs adequately constructed the PT to promote student adherence to its completion. These data provide important feedback for the institutions, highlighting the importance of encouraging students to adopt a longitudinal self-directed learning style and understand the consolidation of the knowledge developed for the proper exercise of the profession [32,33,38]. Therefore, it is up to the pedagogical projects of the courses to point out, in regulatory terms, the tools by which this spiral curricular approach will be worked on in daily practice.

The analysis of students’ motivation to take the PT, the use of results for academic development, and the evolution of performance in the test to guide studies revealed significant statistical differences between the HEIs studied. Students from the private school demonstrated greater agreement regarding these items compared to students from the public institution. This divergence can be explained by the different profiles of students and the insertion of active methodologies in the institutions, which favor interest in formative assessments [41]. One strategy to address this situation would be to conduct the PT more frequently, around two to four times a year, potentially leading to a more positive impact on students’ perception of the test’s value [42].

Surprisingly, no correlation was observed between responses about motivation and the use of PT results by the student for their academic development with the progression of the undergraduate semester in the two medical schools studied. This lack of association may be related to high academic demands, pressure from summative assessments, anxiety about the risk of not progressing in the program, and concern about the proximity of the residency exam, which has a different format from the PT [43]. However, Norman et al. [44] showed that the implementation of PT can reduce failures in medical licensing exams, since conventional exams encourage mechanical memorization in favor of comprehensive reasoning, while, conversely, PT interrupts this effect and allows for deep learning, which may favor performance in other types of exams, such as the medical residency exam [45].

The influence of summative assessments on students’ motivation to learn is another relevant aspect to consider [46]. Given the lack of direct consequences of formative test results, as is the case of PT in the HEIs analyzed in this study, students may be less motivated to put forth their best effort in this assessment. This phenomenon can be explained by the Expectancy-Value Theory (EVT), a conceptual framework frequently used in the context of test-taking motivation [47]. Future research could explore strategies to enhance students’ intrinsic motivation to engage with the PT, potentially improving its effectiveness as a learning tool.

Regarding access to the commented answer key and the PT result, most students from both schools strongly agreed with these items. Research conducted indicates that many students wish to access commented answer keys after taking PTs, as they believe this practice helps them better understand the mistakes made and direct their future studies more effectively [24,48]. However, a significant statistical difference was observed in relation to the item ’The questions are later discussed in the classroom’, in which students from the private institution had a greater perception of the use of PT results compared to students from the public HEI. This finding highlights the importance of understanding how different institutions implement PT to promote learning within each specific reality. In addition to thinking of PT as a subsidy for the institutional validation of curricular practices, it is necessary to promote dialogical reflections that allow the generation of longitudinal feedback to be offered to students, so that this tool can be a valuable resource in the systematization of academic mentoring processes and monitoring of individual acquisition of expected competencies [31].

The adequate approach to curricular content in the institutions studied to take the PT also showed greater agreement among students from the private HEI compared to the public one. Hence the importance of the institutional pedagogical project being aligned with the PT matrix so that students can verify the development of their cognitive performance in the various areas of the course and curriculum, and identify their potential problems, in order to value this exam through this analysis [7]. In addition, it is essential to consider the different assessment cultures in each institution, since schools with traditional teaching tend to value summative assessments, while those that employ active methodologies use a higher proportion of formative assessments [49].

The integration of PT into a programmatic assessment system, as suggested by Van der Vleuten et al. [50], could optimize both the learning function and decision-making in competency-based educational contexts, using well-founded assessment principles. Although this programmatic assessment model is well received in educational practice [51,52], many consider it complex and theoretical. To address this, Van der Vleuten et al. [53] described concrete tips for implementing programmatic assessment in educational institutions. Future research could explore the effectiveness of these implementation strategies across diverse educational settings.

The uniqueness of the present study is linked to its relevance in assessing medical students’ perceptions of PT, which can be used by academic managers to develop new interventions in the curriculum, improving the quality of health courses. A limitation of this study is the sample restricted to two institutions, which may limit the generalization of the results to other contexts. In addition, the research was based exclusively on student perceptions, not covering the perspective of other actors involved in the process, such as teachers and educational managers. Thus, as a future perspective, more research is needed in other educational institutions to compare and deepen the understanding of the impact of PT on the teaching-learning process of medical students, as well as to verify the effect that any changes in curricular design have on these indicators.

It is important to emphasize that structural modifications in the educational system are not sufficient; a fundamental reformulation of educational models is imperative. Higher education institutions must move beyond the model of transmitting information disconnected from practical reality. Pedagogical processes need to emphasize debate and critical reflection, with teachers acting as facilitators and catalysts, while students take active responsibility for their learning [54]. Students must understand the meaning and applicability of what they learn, using information critically and developing the skills and competencies necessary to become professionals capable of transforming their environment.

## Conclusions

This study examined medical students’ perceptions of the Progress Test (PT) design and implementation across two distinct institutions. Our findings underscore the PT’s significance as a continuous, formative assessment tool that enables the monitoring of knowledge development throughout the medical curriculum.

The research revealed both strengths and challenges in PT implementation, highlighting the need for alignment between PT content and course curricula, as well as the importance of providing individualized feedback to students. These insights can inform strategies to optimize PT implementation and enhance its effectiveness as a learning tool.

By considering student perspectives, educational institutions can develop more meaningful assessment practices that support the development of competent medical professionals. This study contributes to the field of medical education assessment, offering evidence-based insights for enhancing Progress Tests and opening avenues for future research in this area.

## Supporting information

S1 Appendix The complete questionnaire, in both Portuguese (original language) and English. (DOCX)

S1 File (PDF)

S2 File (PDF)

S3 File (PDF)

S4 File (PDF)
