# Supplementary material for: Continuous assessment in medical education: Exploring students’ views on the progress test
Source: PLoS One. 2024 Dec 19;19(12):e0314848. doi: 10.1371/journal.pone.0314848 (PMC11658631; doi:10.1371/journal.pone.0314848)
Supplement: S1 Appendix — (DOCX) [file pone.0314848.s001.docx]

Appendix A: The complete questionnaire, in both Portuguese (original language) and English.

|  | *Portuguese (original language)* | *English* |
| --- | --- | --- |
| Section 1: Demographics: | | |
| Section 2: Perceptions of PT Construction and Institutional Movements | | |
| 1. | Os enunciados das questões e as alternativas são claras para responder. | The wording of the questions and the alternatives are clear to answer. |
| 2. | O tempo de realização é adequado para o conteúdo. | The time taken to complete the test is adequate for the content. |
| 3. | Recebi informação prévia da Instituição sobre a importância do teste. | I have received prior information from the institution about the importance of the test. |
| 4 | Estou motivado para fazer o teste. | I am motivated to take the test. |
| Section 3: Access to Commented Answer Key and PT Results: | | |
| 5 | Pretendo acessar o gabarito comentado. | I intend to access the commented template. |
| 6 | Pretendo acessar o resultado. | I intend to access the result. |
| 7 | As questões são posteriormente discutidas na sala de aula. | The questions are then discussed in the classroom. |
| 8 | A discussão das questões em sala de aula é importante. | Discussing the questions in class is important. |
| 9 | O conteúdo abordado em minha Instituição é adequado para a realização do teste. | The content covered at my institution is suitable for the test. |
| 10 | Reconheço a importância da realização do teste para o desenvolvimento acadêmico | I recognise the importance of taking the test for academic development |
| 11 | Levo em conta os resultados no teste para avaliar o desenvolvimento acadêmico. | I take test results into account to assess academic development. |
| 12 | Levo em conta a evolução do desempenho no teste para nortear os estudos | I take into account the evolution of performance in the test to guide my studies |
| Section 2 and 3 answered on a 5-point Likert scale (Strongly disagree, Partially disagree, Neither agree nor disagree, Partially agree, Fully agree) | | |
