# Supplementary material for: Continuous assessment in medical education: Exploring students’ views on the progress test
Source: PLoS One. 2024 Dec 19;19(12):e0314848. doi: 10.1371/journal.pone.0314848 (PMC11658631; doi:10.1371/journal.pone.0314848)
Supplement: S1 File — (PDF) [file pone.0314848.s002.pdf]

Relatório dos resultados do estudo:

O teste de progresso de quem realiza o mesmo: o discente

São José do Rio Preto, 8 de setembro de 2021

SUMÁRIO

I. Metodologia da análise estatística 3

1. Caracterização da amostra 3
2. Banco de dados 3
3. Análise estatística dos dados 3

II. Resultados 4

1. ANALISE 1 (FAMERP) 4
2. ANALISE 2 (comparação entre as duas IES) 26

III. Referências 33

I - Metodologia da análise estatística

1. Caracterização da amostra

A amostra do estudo foi composta por 220 alunos da FAMERP e 709 alunos da UNISA.

2. Banco de dados

Os dados foram recebidos cadastrados no Excel. Posteriormente, foram importados para o software IBM-SPSS *Statistics* versão 28 (IBM Corporation, NY, USA) para análise exploratória dos dados e análise comparativa entre grupos.

3. Análise estatística dos dados

A análise exploratória dos dados incluiu as estatísticas descritivas, média, mediana, desvio-padrão, valor mínimo e valor máximo para variáveis numéricas e número e proporção para variáveis categóricas. A análise de correlação de Spearman foi realizada para verificar a correlação entre as variáveis discretas e ordinais (semestre da graduação e item do questionário); o resultado foi demostrando pelo coeficiente de correlação de Spearman e seu respectivo intervalo de confiança de 95% (BONETT & WRIGHT, T. A, 2000; CONOVER, 1999; SIEGEL & CASTELLAN, 2006; BISHARA & HITTNER, 2017). A comparação entre alunos das duas IES foi realizada pelo teste Qui-quadrado de Pearson ou exato de Fisher (CONOVER, 1999; SIEGEL & CASTELLAN, 2006; FIELD, 2009) Ao se encontrar significância estatística, realizou-se comparações múltiplas (método *pairwise*) pelo teste z para diferença de proporções (BUSSAB & MORETTIN, 2017), com valores de p ajustados pela correção de Bonferroni, para se verificar onde estava o efeito das diferenças; os resultados foram expressos nas tabelas em número e porcentagem, com notações em letras sinalizando as semelhanças ou diferença entre as categorias (IBM SPSS Statistics Algorithms, 2020; MACDONALD & GARDNER, 2000). Análise estatística foi realizada mediante os softwares IBM-SPSS *Statistics* versão 28 (IBM Corporation, NY, USA) e R (R CORE TEAM, 2015).

II – Resultados

ANÁLISE 1: FAMERP

1. Análise descritiva da amostra

A Tabela abaixo demonstra a faixa etária e semestre da graduação, entre os 220 alunos que participaram da pesquisa.

Tabela
Faixa etária e semestre de graduação entre os 220 participantes incluídos no estudo.

Variável	N = 220
Idade, n (%)	
17 a 20 anos	32 (14,5)
21 a 25 anos	131 (59,5)
26 a 30 anos	38 (17,3)
31 a 35 anos	5 (2,3)
Acima de 40 anos	1 (0,5)
Não respondeu	13 (5,9)
Semestre de graduação, n (%)	
2º. Semestre do 1º ano (2º Semestre)	46 (20,9)
2º. Semestre do 2º ano (4º Semestre)	32 (14,5)
2º. Semestre do 3º ano (6º Semestre)	42 (19,1)
2º. Semestre do 5º ano (10º Semestre)	37 (16,8)
2º. Semestre do 6º ano (12º Semestre)	50 (22,8)
Não respondeu	13 (5,9)

Variáveis categóricas estão descritas em número (porcentagem)

Comentário: não participaram da pesquisa alunos do 2º. Semestre do 4º ano (8º Semestre) do curso.

2. Análise descritiva das respostas às perguntas do instrumento aplicado

A seguir, a Tabela abaixo demonstra a distribuição das respostas dos alunos aos itens aplicados, em relação à autopercepção do aluno no desempenho esperado no TP.

Tabela
Autopercepção do aluno no desempenho esperado no Teste de Progresso.

Itens	N (%)
Porcentagem de questões que espera acertar, n (%)	
0 a 20%	32 (14,5)
20 a 40%	52 (23,6)
40 a 60%	44 (20)
60 a 80%	55 (25)
80 a 100%	23 (10,5)
Não respondeu	14 (6,4)
Área que acredita ter melhor desempenho, n (%)	
Básica	71 (32,4)
Saúde coletiva	44 (20)
Clínica	41 (18,6)
Cirurgia	30 (13,6)
GO	11 (5)
Pediatra	10 (4,5)
Não respondeu	13 (5,9)

GO, ginecologia obstetrícia.

A Tabela abaixo demonstra a distribuição das respostas dos alunos aos itens, em relação à adequação da construção do TP e possíveis movimentos institucionais para promover a adesão do acadêmico na realização do mesmo.

Tabela
Adequação da construção do TP e possíveis movimentos institucionais para promover a adesão do acadêmico na realização do mesmo.

Itens	N (%)
Os enunciados das questões e as alternativas são claras para responder, n (%)	
Discordo parcialmente	9 (4,1)
Não concordo nem discordo	17 (7,7)
Concordo parcialmente	90 (40,9)
Concordo plenamente	91 (41,4)
Não respondeu	13 (5,9)
Tempo de realização é adequado para o conteúdo, n (%)	
Discordo plenamente	11 (5)
Discordo parcialmente	14 (6,4)
Não concordo nem discordo	21 (9,5)
Concordo parcialmente	44 (20)
Concordo plenamente	117 (53,2)
Não respondeu	13 (5,9)
Recebeu informação prévia da Instituição sobre a importância do teste, n (%)	
Discordo plenamente	1 (0,5)
Discordo parcialmente	9 (4,1)
Não concordo nem discordo	11 (5)
Concordo parcialmente	30 (13,6)
Concordo plenamente	156 (70,9)
Não respondeu	13 (5,9)

Variáveis categóricas estão descritas em número (porcentagem)

A Tabela abaixo mostra a distribuição das respostas dos alunos aos itens sobre se pretende acessar o gabarito comentado e o resultado do TP.

Tabela
Itens sobre se pretende acessar o gabarito comentado e o resultado do TP.

Variáveis	N (%)
Pretende acessar o gabarito comentado, n (%)	
Discordo plenamente	3 (1,4)
Discordo parcialmente	8 (3,6)
Não concordo nem discordo	14 (6,4)
Concordo parcialmente	31 (14,1)
Concordo plenamente	151 (68,6)
Não respondeu	13 (5,9)
Pretende acessar o resultado, n (%)	
Discordo plenamente	2 (0,9)
Discordo parcialmente	2 (0,9)
Não concordo nem discordo	10 (4,5)
Concordo parcialmente	21 (9,5)
Concordo plenamente	172 (78,3)
Não respondeu	13 (5,9)

Variáveis categóricas estão descritas em número (porcentagem)

A Tabela abaixo mostra a distribuição das respostas dos alunos quanto ao aproveitamento dos resultados do TP pela IES.

Tabela
Aproveitamento dos resultados do TP pela IES.

Itens	N (%)
As questões são posteriormente discutidas na sala de aula, n (%)	
Discordo plenamente	128 (58,2)
Discordo parcialmente	22 (10)
Não concordo nem discordo	47 (21,4)
Concordo parcialmente	2 (0,9)
Concordo plenamente	6 (2,7)
Não respondeu	15 (6,8)
Importância da discussão das questões em sala de aula, n (%)	
Discordo parcialmente	1 (0,5)
Não concordo nem discordo	14 (6,4)
Concordo parcialmente	50 (22,6)
Concordo plenamente	141 (64,1)
Não respondeu	14 (6,4)
O conteúdo abordado em sua Instituição é adequado para a realização do teste, n (%)	
Discordo parcialmente	13 (5,9)
Não concordo nem discordo	45 (20,5)
Concordo parcialmente	94 (42,7)
Concordo plenamente	54 (24,5)
Não respondeu	14 (6,4)

TP, Teste de Progresso; IES, Instituição de Ensino Superior.

A Tabela abaixo demonstra a distribuição das respostas dos alunos aos itens que caracterizam a motivação e uso dos resultados do TP pelo próprio aluno para seu desenvolvimento acadêmico.

Tabela
Motivação e uso dos resultados do TP pelo próprio aluno para seu desenvolvimento acadêmico

Itens	N (%)
Motivado para fazer o teste, n (%)	
Discordo plenamente	16 (7,3)
Discordo parcialmente	25 (11,4)
Não concordo nem discordo	40 (18,2)
Concordo parcialmente	58 (26,3)
Concordo plenamente	67 (30,4)
Não respondeu	14 (6,4)
Importância da realização do teste para o desenvolvimento acadêmico, n (%)	
Discordo plenamente	6 (2,7)
Discordo parcialmente	10 (4,5)
Não concordo nem discordo	21 (9,5)
Concordo parcialmente	64 (29,1)
Concordo plenamente	105 (47,8)
Não respondeu	14 (6,4)
Leva em conta o desenvolvimento no teste para avaliar o desenvolvimento acadêmico, n (%)	
Discordo plenamente	19 (8,6)
Discordo parcialmente	18 (8,2)
Não concordo nem discordo	25 (11,4)
Concordo parcialmente	51 (23,2)
Concordo plenamente	52 (23,6)
Não respondeu	55 (25)
Leva em conta a evolução do desempenho no teste para nortear os estudos, n (%)	
Discordo plenamente	24 (10,9)
Discordo parcialmente	25 (11,4)
Não concordo nem discordo	31 (14,1)
Concordo parcialmente	48 (21,8)
Concordo plenamente	37 (16,8)
Não respondeu	55 (25)

Variáveis categóricas estão descritas em número (porcentagem).

Resultados da autopercepção do aluno no desempenho esperado no TP, segundo o semestre cursado.

Tabela
Distribuição das respostas ao item "Porcentagem de questões que espera acertar", segundo o semestre de graduação cursado.

Série de graduação	Porcentagem de questões que espera acertar	N (%)
2º. Semestre do 1º ano (2º Semestre)		
	0 a 20%	31/46 (67,4)
	20 a 40%	14/46 (30,4)
	80 a 100%	1/46 (2,2)
2º. Semestre do 2º ano (4º Semestre)		
	0 a 20%	1/32 (3,1)
	20 a 40%	17/32 (53,1)
	40 a 60%	12/32 (37,5)
	60 a 80%	2/32 (6,3)
2º. Semestre do 3º ano (6º Semestre)		
	20 a 40%	17/42 (40,5)
	40 a 60%	21/42 (50)
	60 a 80%	4/42 (9,5)
2º. Semestre do 5º ano (10º Semestre)		
	20 a 40%	2/36 (5,6)
	40 a 60%	5/36 (13,9)
	60 a 80%	25/36 (69,4)
	80 a 100%	4/36 (11,1)
2º. Semestre do 6º ano (12º Semestre)		
	20 a 40%	2/50 (4)
	40 a 60%	6/50 (12)
	60 a 80%	24/50 (48)
	80 a 100%	18/50 (36)

Variáveis categóricas estão descritas em número (porcentagem).

3. Análise de correlação

A análise de correlação é adequada quando se estuda a relação entre duas variáveis que tenham natureza numérica ou ordinal. O coeficiente de correlação é mensurado por meio da escala de valores +1 a -1; quando o valor está próximo de +1, assume-se a correlação linear positiva perfeita (ou seja, quanto maior o valor de uma variável, maior também será o valor da outra variável), e quando o valor do coeficiente está próximo de -1, assume-se a correlação linear negativa perfeita (ou seja, quanto maior o valor de uma variável, menor será o valor da outra); os valores próximos de zero indicam a ausência de correlação. A força da correlação entre duas variáveis pode ser interpretada da seguinte forma, segundo a literatura: r |≤0,25| = ausência de correlação; |0,26 – 0,50| = correlação fraca; |0,51 – 0,75| = correlação moderada e |>0,75| = correlação forte. O resultado do "r" é aquele encontrado na amostra. Por outro lado, o IC 95% (intervalo de confiança) mostra os valores de r extrapolados para a população, com confiança de 95%. Além disso, a visualização por gráficos de dispersão pode auxiliar na interpretação de existência ou não de correlação, pela forma como os valores dos eixos X e Y se relacionam.

Houve correlação positiva forte entre o semestre da graduação e a percepção do aluno sobre a porcentagem de questões que espera acertar, ou seja, conforme aumenta o semestre da graduação, maior foi a porcentagem de questões que o aluno espera acertar. A tabela abaixo demonstra o valor do coeficiente de correlação (r_S) e o seu intervalo de confiança. Da mesma forma, podemos visualizar a correlação entre as duas variáveis, através do gráfico de dispersão.

Tabela
Correlação entre semestre de graduação e o item "Porcentagem de questões que espera acertar".

	Semestre da graduação	
Item	r_S	IC (95%)
Porcentagem de questões que espera acertar	0,817	0,756; 0,863

r_S = coeficiente de correlação de Spearman; IC, intervalo de confiança.

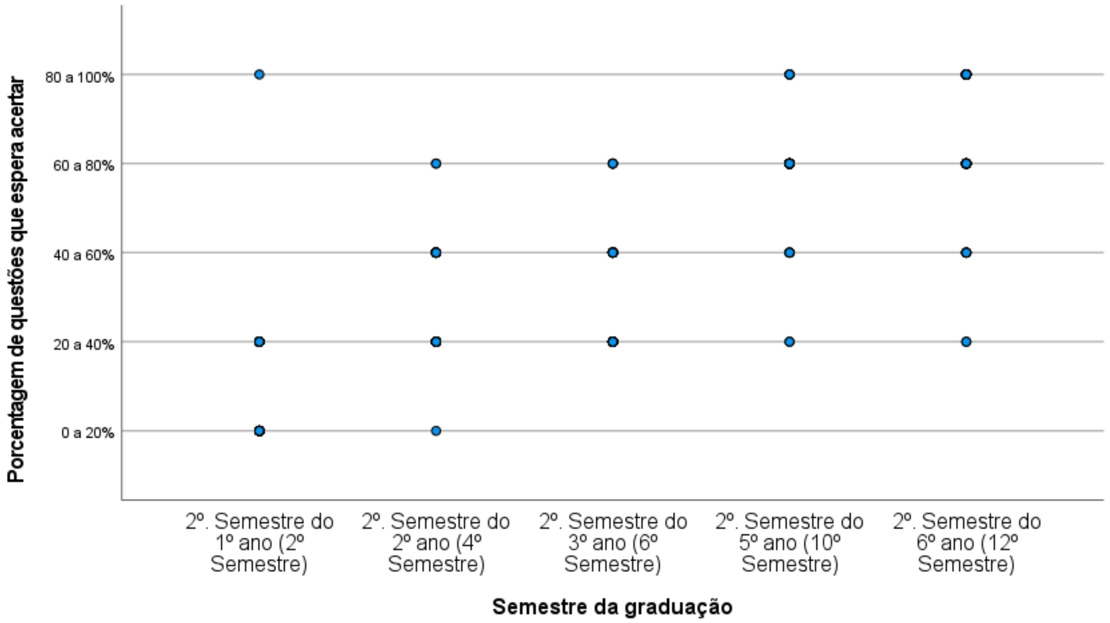

FIGURA

Gráfico de dispersão entre o item "Porcentagem de questões que espera acertar" e semestre da graduação.

A seguir, é apresentada a análise de correlação entre o semestre da graduação (2º semestre de cada ano de curso) e as respostas a cada um dos 12 itens do instrumento, que permitiam resposta em escala tipo Likert.

Na Tabela abaixo, podemos observar a correlação entre as respostas acerca da adequação da construção do TP e possíveis movimentos institucionais para promover a adesão do acadêmico na realização do mesmo e o semestre da graduação. Quanto ao item "Tempo de realização é adequado para o conteúdo", podemos observar que houve uma fraca correlação positiva (observar o IC 95%), ou seja, conforme avançamos no semestre, as pontuações mais baixas foram desaparecendo e permanecem as pontuações mais altas de resposta (observar o gráfico de dispersão). Por outro lado, para os itens "Os enunciados das questões e as alternativas são claras para responder" e "Recebeu informação prévia da Instituição sobre a importância do teste" considera-se que não houve correlação.

Tabela
Correlação entre o semestre de graduação e as respostas sobre adequação da construção do TP e possíveis movimentos institucionais para promover a adesão do acadêmico na realização do mesmo *.

	Semestre de graduação	
Item	r_s	IC (95%)
Os enunciados das questões e as alternativas são claras para responder	0,087	-0,050; 0,221
Tempo de realização é adequado para o conteúdo	0,441	0,318; 0,549
Recebeu informação prévia da Instituição sobre a importância do teste	0,203	0,067; 0,332

r_s = coeficiente de correlação de Spearman; IC, intervalo de confiança.
*As respostas às perguntas do instrumento foram relatadas através de uma escala tipo Likert, onde: 0 = discordo plenamente; 1 = discordo parcialmente; 2 = não concordo e nem discordo; 3 = concordo parcialmente; 4 = concordo plenamente.

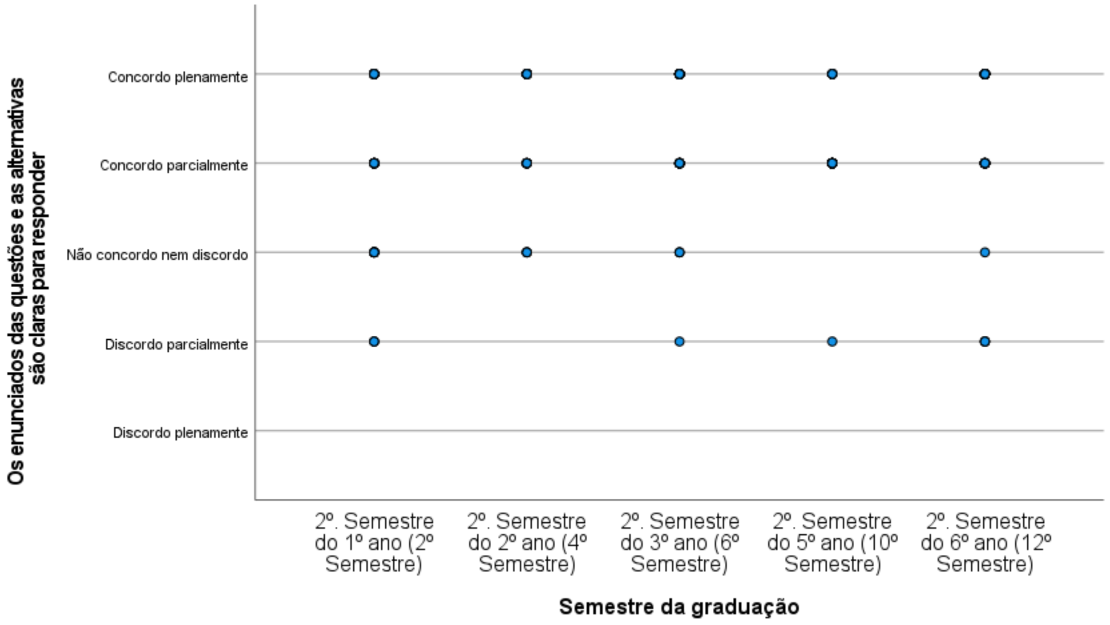

FIGURA

Gráfico de dispersão entre o item "Os enunciados das questões e as alternativas são claras para responder" e semestre da graduação.

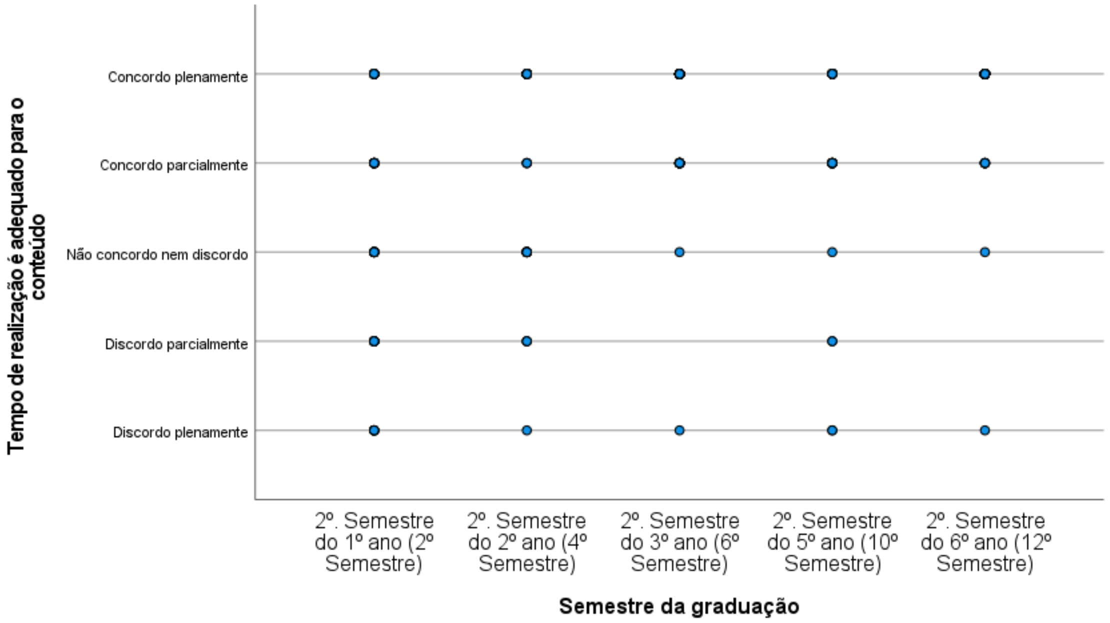

FIGURA

Gráfico de dispersão entre o item "O tempo de realização é adequado para o conteúdo" e semestre da graduação.

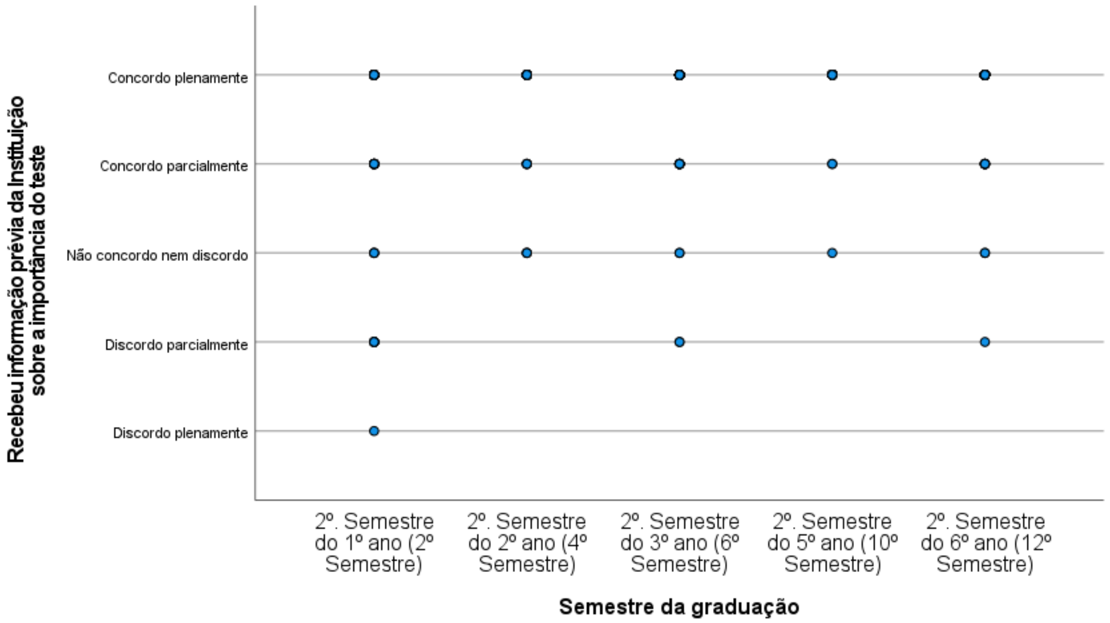

FIGURA

Gráfico de dispersão entre o item “Recebeu informação prévia da Instituição sobre a importância do teste” e semestre da graduação.

Na Tabela abaixo, podemos observar a correlação entre as respostas sobre se pretende acessar o gabarito comentado e resultado do TP e o semestre da graduação. Quanto ao item “Pretende acessar o gabarito comentado”, podemos observar que houve uma fraca correlação positiva (observar o IC 95%), ou seja, conforme avançamos no semestre, as pontuações mais baixas foram desaparecendo e permanecem as pontuações mais altas de resposta (observar o gráfico de dispersão). Por outro lado, para o item “Pretende acessar o resultado” considera-se que não houve correlação.

Tabela
Correlação entre o semestre da graduação e as respostas sobre se pretende acessar o gabarito comentado e resultado do TP*

	Semestre de graduação	
Item	r_s	IC (95%)
Pretende acessar o gabarito comentado	0,296	0,164; 0,418
Pretende acessar o resultado	0,095	-0,042; 0,229

r_s = coeficiente de correlação de Spearman; IC, intervalo de confiança.
*As respostas às perguntas do instrumento foram relatadas através de uma escala tipo Likert, onde: 0 = discordo plenamente; 1 = discordo parcialmente; 2 = não concordo e nem discordo; 3 = concordo parcialmente; 4 = concordo plenamente.

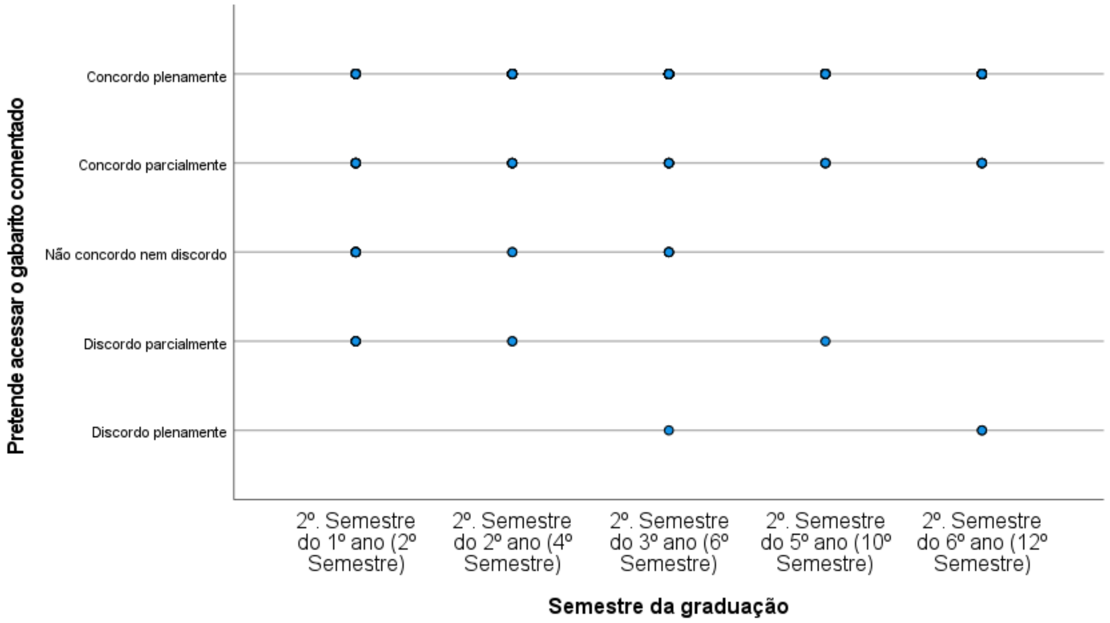

FIGURA

Gráfico de dispersão entre o item ‘‘Pretende acessar o gabarito comentado” e semestre da graduação.

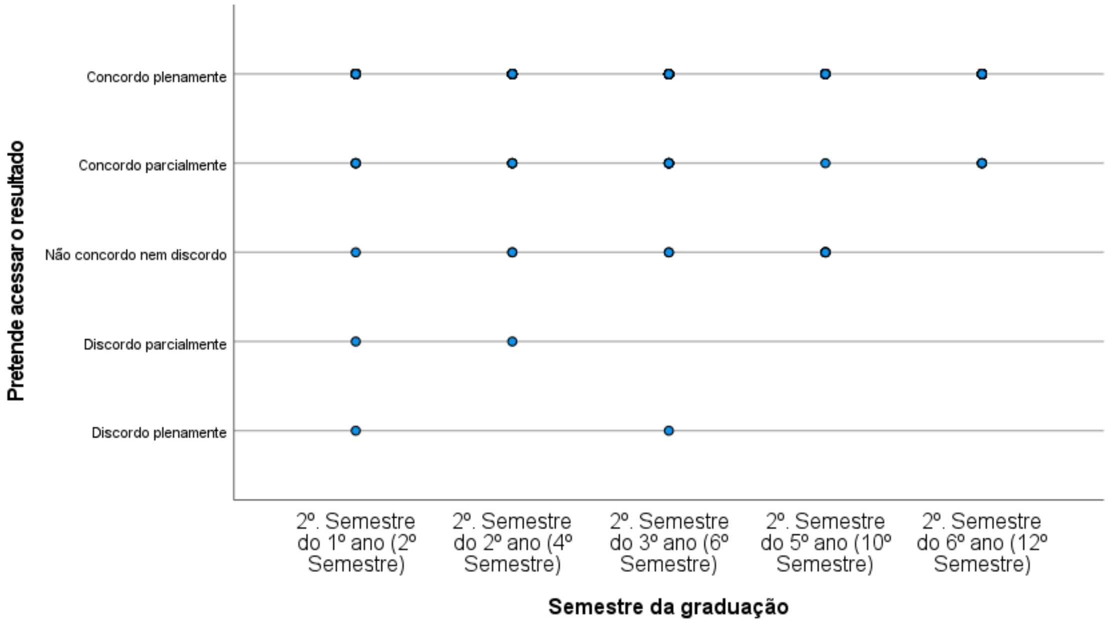

FIGURA

Gráfico de dispersão entre o item “Pretende acessar o resultado” e semestre da graduação.

Na Tabela abaixo, podemos observar a correlação entre as respostas quanto ao aproveitamento dos resultados do TP pela IES e o semestre da graduação. O coeficiente de correlação para o item "As questões são posteriormente discutidas na sala de aula" demonstrou fraca correlação negativa (confirmada pelo intervalo de confiança de 95%), ou seja, conforme mais avançado o semestre, menor a pontuação das respostas em escala Likert.

A percepção do aluno quanto às demais questões foi independente do semestre cursado, como verificado pelo coeficiente de correlação. Observar os respectivos gráficos de dispersão.

Tabela
Correlação entre o semestre de graduação e as respostas quanto ao aproveitamento dos resultados do TP pela IES*.

	Semestre da graduação	
Item	r_s	IC (95%)
As questões são posteriormente discutidas na sala de aula	-0,411	-0,523; -0,285
Importância da discussão das questões em sala de aula	0,154	0,012; 0,290
O conteúdo abordado em sua Instituição é adequado para a realização do teste	0,157	0,015; 0,293

r_s = coeficiente de correlação de Spearman; IC, intervalo de confiança.

*As respostas às perguntas do instrumento foram relatadas através de uma escala tipo Likert, onde: 0 = discordo plenamente; 1 = discordo parcialmente; 2 = não concordo e nem discordo; 3 = concordo parcialmente; 4 = concordo plenamente.

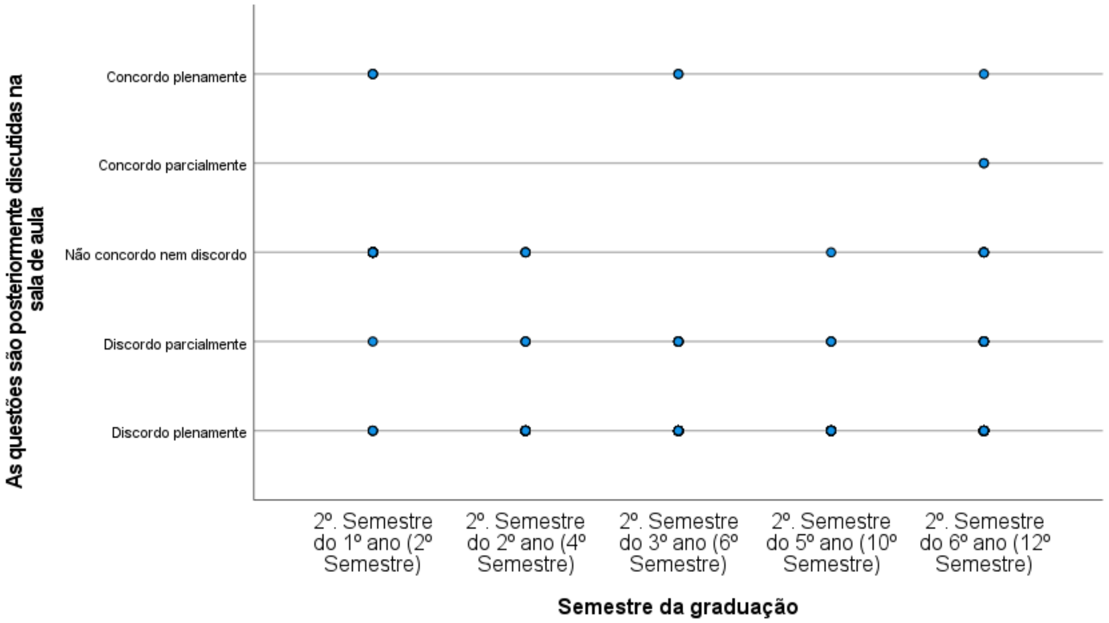

FIGURA

Gráfico de dispersão entre o item “As questões são posteriormente discutidas na sala de aula” e semestre da graduação.

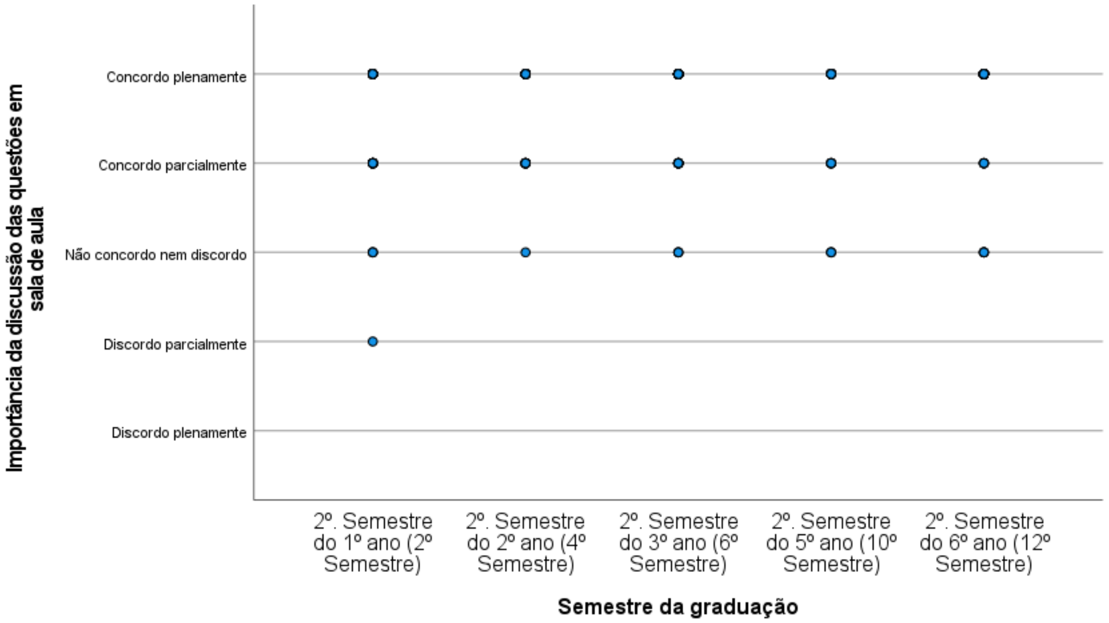

FIGURA

Gráfico de dispersão entre o item “Importância da discussão das questões em sala de aula” e semestre da graduação.

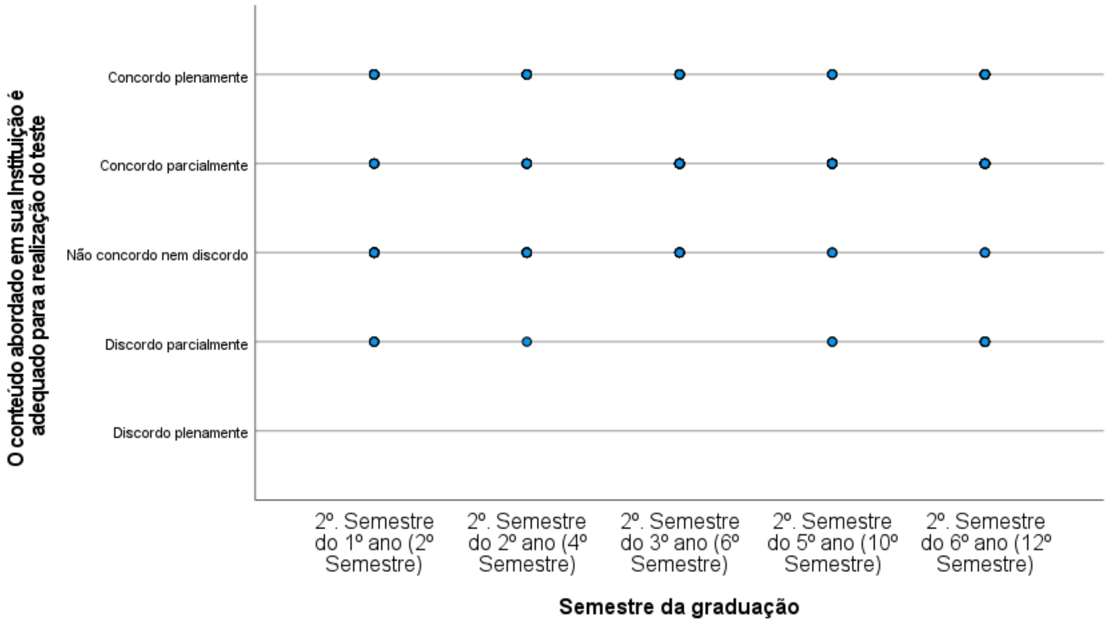

FIGURA

Gráfico de dispersão entre o item “O conteúdo abordado em sua Instituição é adequado para a realização do teste” e semestre da graduação.

Na Tabela abaixo, podemos observar que não houve correlação entre as respostas acerca da motivação e uso dos resultados do TP pelo próprio aluno para seu desenvolvimento acadêmico e o semestre da graduação. Portanto, a percepção do aluno quanto a estas questões foi independente do semestre cursado. Isto está ilustrado nos respectivos gráficos de dispersão.

Tabela
Correlação entre a semestre de graduação e as respostas quanto à motivação e uso dos resultados do TP pelo próprio aluno para seu desenvolvimento acadêmico *.

	Série de graduação	
Item	r_s	IC (95%)
Motivado para fazer o teste	0,147	0,005;0,283
Importância da realização do teste para o desenvolvimento acadêmico	-0,001	-0,142; 0,140
Leva em conta o desenvolvimento no teste para avaliar o desenvolvimento acadêmico	0,093	-0,068; 0,249
Leva em conta a evolução do desempenho no teste para nortear os estudos	0,075	-0,085; 0,232

r_s = coeficiente de correlação de Spearman; IC, intervalo de confiança.
*As respostas às perguntas do instrumento foram relatadas através de uma escala tipo Likert, onde: 0 = discordo plenamente; 1 = discordo parcialmente; 2 = não concordo e nem discordo; 3 = concordo parcialmente; 4 = concordo plenamente.

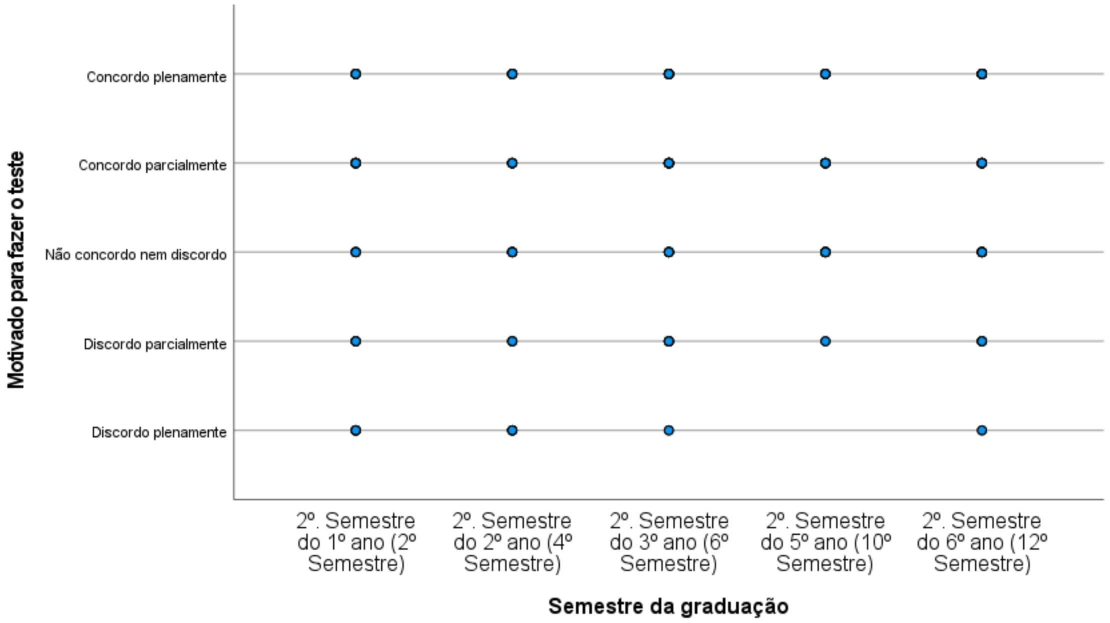

FIGURA

Gráfico de dispersão entre o item “Motivado para fazer o teste” e semestre da graduação.

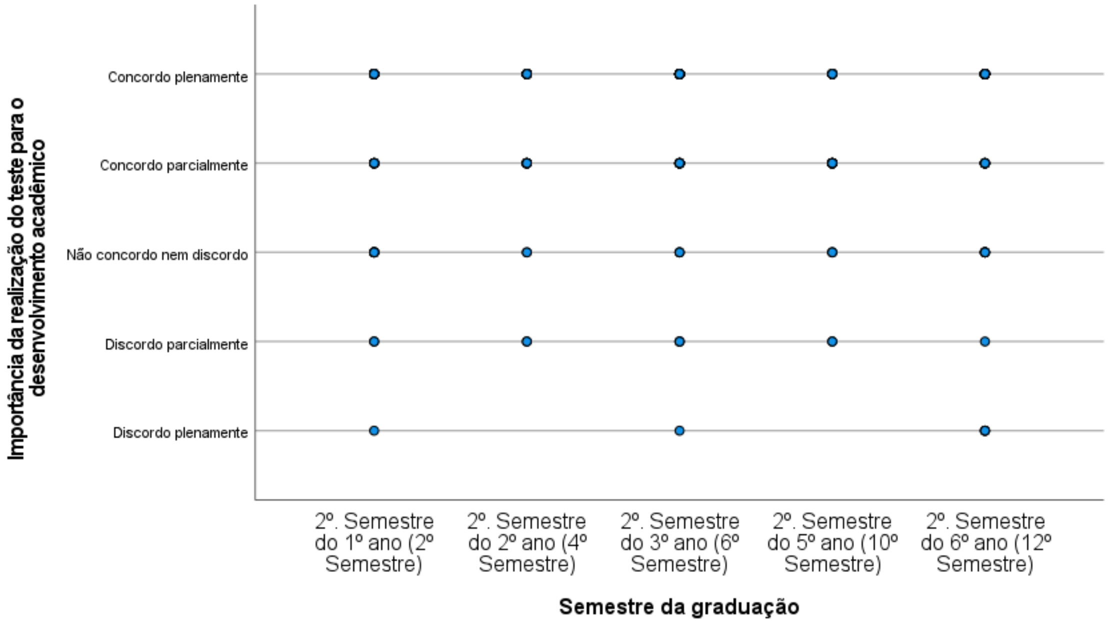

FIGURA

Gráfico de dispersão entre o item “Importância da realização do teste para o desenvolvimento acadêmico” e semestre da graduação.

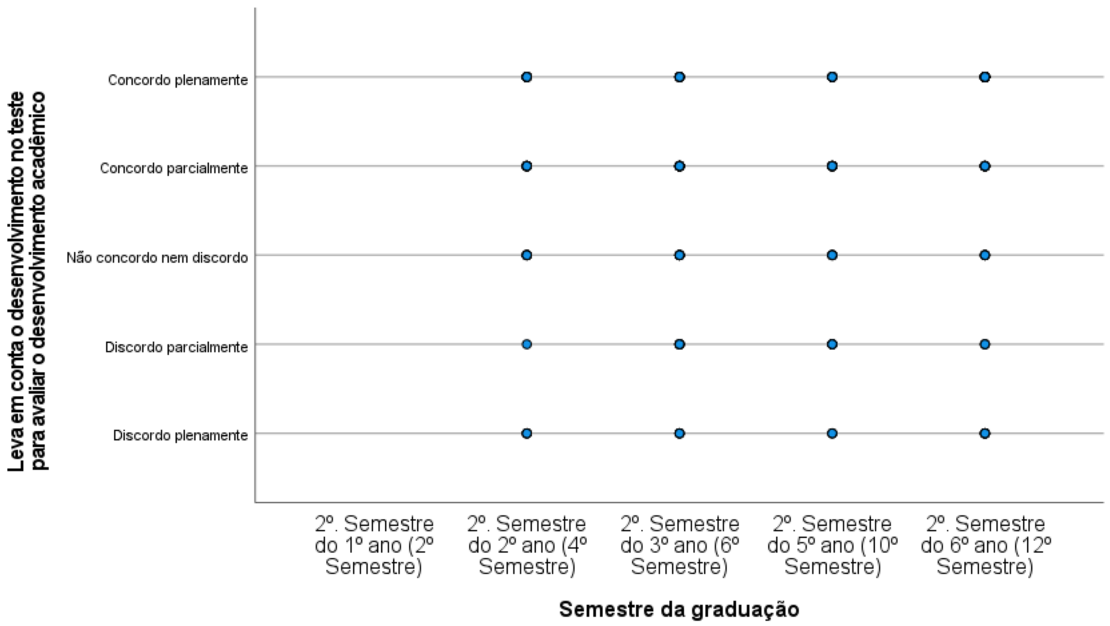

FIGURA

Gráfico de dispersão entre o item "Leva em conta o desenvolvimento no teste para avaliar o desenvolvimento acadêmico" e semestre da graduação.

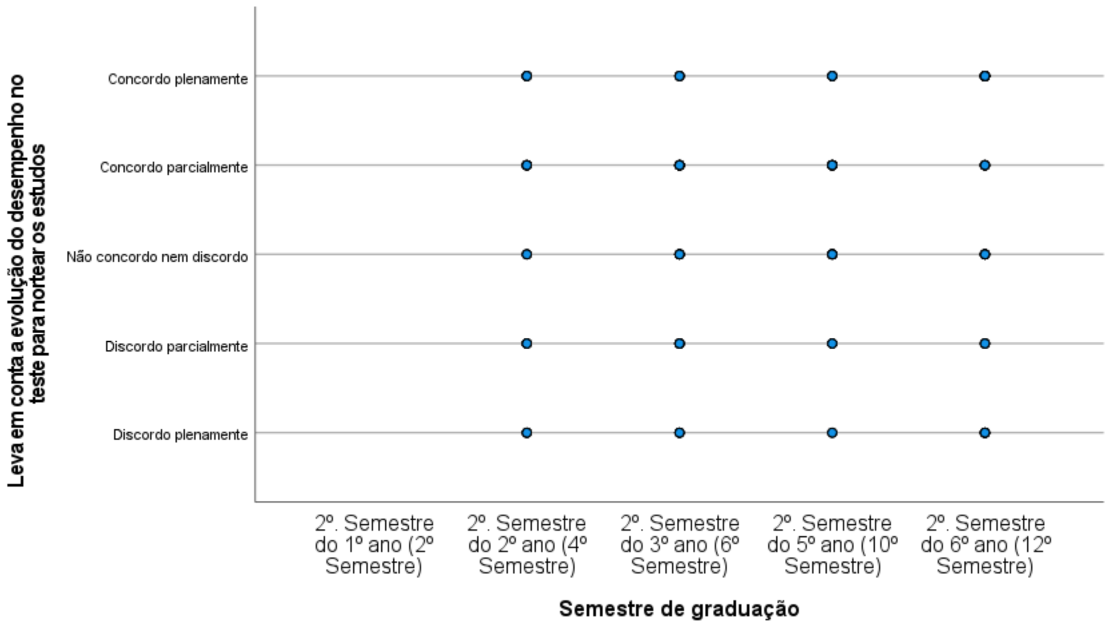

FIGURA

Gráfico de dispersão entre o item "Leva em conta a evolução do desempenho no teste para nortear os estudos" e o semestre da graduação.

ANÁLISE 2: Análise comparativa entre as duas IES

4. Análise descritiva da amostra

Tabela

Faixa etária e ano de graduação dos alunos das duas IES.

	IES	
	UNISA	FAMERP
Variável	N = 709	N = 220
Idade, n (%)		
17 a 20 anos	263 (37,1)	32 (14,5)
21 a 25 anos	350 (49,3)	131 (59,5)
26 a 30 anos	67 (9,4)	38 (17,3)
31 a 35 anos	16 (2,3)	5 (2,3)
36 a 39 anos	4 (0,6)	0 (0,0)
Acima de 40 anos	9 (1,3)	1 (0,5)
Não respondeu	0 (0,0)	13 (5,9)
Ano de graduação, n (%)		
1ºAno	260(36,7)	46 (20,9)
2ºAno	148(20,9)	32 (14,5)
3ºAno	99(14)	42 (19,1)
4ºAno	67(9,4)	0 (0,0)
5ºAno	61(8,6)	37 (16,8)
6ºAno	74(10,4)	50 (22,8)
Não respondeu	0 (0,0)	13 (5,9)

Variáveis categóricas estão descritas em número (porcentagem); 1ºAno = 1º. semestre e 2º. semestre; 2ºAno = 3º. semestre e 4º. semestre; 3ºAno = 5º. semestre e 6º. semestre; 4ºAno = 7º. semestre e 8º. semestre; 5ºAno = 9º. semestre e 10º. semestre; 6ºAno = 11º. semestre e 12º. Semestre; IES, instituição de ensino superior.

Tabela
Distribuição das respostas do item “Porcentagem de questões que espera acertar”, segundo o ano de graduação cursado pelos alunos das duas IES.

Ano de graduação	Porcentagem de questões que espera acertar	IES	
		UNISA	FAMERP
1º ano			
	0 a 20%	157 (60,4)	31/46 (67,4)
	20 a 40%	86 (33,1)	14/46 (30,4)
	40 a 60%	10 (3,8)	0/46 (0,0)
	60 a 80%	2 (0,8)	0/46 (0,0)
	80 a 100%	5 (1,9)	1/46 (2,2)
2º ano			
	0 a 20%	28 (18,9)	1/32 (3,1)
	20 a 40%	90 (60,8)	17/32 (53,1)
	40 a 60%	26 (17,6)	12/32 (37,5)
	60 a 80%	3 (2,0)	2/32 (6,3)
	80 a 100%	1 (0,7)	0/32 (0,0)
3º ano			
	0 a 20%	2 (2,0)	0/42 (0,0)
	20 a 40%	32 (32,3)	17/42 (40,5)
	40 a 60%	48 (48,6)	21/42 (50)
	60 a 80%	13 (13,1)	4/42 (9,5)
	80 a 100%	4 (4,0)	0/42 (0,0)
4º ano			
	20 a 40%	6 (9,0)	0 (0,0)
	40 a 60%	37 (55,2)	0 (0,0)
	60 a 80%	23 (34,3)	0 (0,0)
	80 a 100%	1 (1,5)	0 (0,0)
5º ano			
	0 a 20%	1 (1,6)	2/36 (5,6)
	20 a 40%	1 (1,6)	0/36 (0,0)
	40 a 60%	27 (44,3)	5/36 (13,9)
	60 a 80%	30 (49,2)	25/36 (69,4)
	80 a 100%	2 (3,3)	4/36 (11,1)
6º ano			
	0 a 20%	0 (0,0)	2/50 (4)
	20 a 40%	1 (1,4)	0/50 (0,0)
	40 a 60%	22 (29,7)	6/50 (12)
	60 a 80%	46 (62,1)	24/50 (48)
	80 a 100%	5 (6,8)	18/50 (36)

1ºAno = 1º. semestre e 2º. semestre; 2ºAno = 3º. semestre e 4º. semestre; 3ºAno = 5º. semestre e 6º. semestre; 4ºAno = 7º. semestre e 8º. semestre; 5ºAno = 9º. semestre e 10º. semestre; 6ºAno = 11º. semestre e 12º. Semestre; IES, instituição de ensino superior.

5. Análise comparativa entre as IES

Tabela
Análise comparativa do item "área que acredita ter melhor desempenho" entre os alunos das duas IES.

		IES			
Item	Respostas	UNISA	FAMERP	Total	Valor P
Área que acredita ter melhor desempenho	Básica	255	71	326	0,117
		36,0%	34,3%	35,6%	
	Clínica	127	41	168	
		17,9%	19,8%	18,3%	
	Cirurgia	74	30	104	
		10,4%	14,5%	11,4%	
	GO	78	11	89	
		11,0%	5,3%	9,7%	
	Pediatria	41	10	51	
		5,8%	4,8%	5,6%	
	Saúde coletiva	134	44	178	
		18,9%	21,3%	19,4%	
	Total	709	207	916	
		100,0%	100,0%	100,0%	

Teste Qui-quadrado de Pearson; IES, instituição de ensino superior.

Tabela
Análise comparativa dos itens sobre adequação do TP e possíveis movimentos institucionais para promover a adesão do acadêmico na realização do TP entre os alunos das duas IES.

Item	Respostas	IES			Valor P
		UNISA	FAMERP	Total	
Os enunciados das questões e as alternativas são claras para responder	Discordo plenamente	2	0	2	0,101
		0,3%	0,0%	0,2%	
	Discordo parcialmente	22	9	31	
		3,1%	4,3%	3,4%	
	Não concordo nem discordo	90	17	107	
		12,8%	8,2%	11,8%	
	Concordo parcialmente	248	9	338	
		35,3%	43,5%	37,1%	
	Concordo plenamente	341	91	432	
		48,5%	44,0%	47,5%	
	Total	703	207	910	
		100,0%	100,0%	100,0%	
Tempo de realização é adequado para o conteúdo	Discordo plenamente	23	11	34	0,689
		3,3%	5,3%	3,7%	
	Discordo parcialmente	51	14	65	
		7,2%	6,8%	7,1%	
	Não concordo nem discordo	78	21	99	
		11,0%	10,1%	10,8%	
	Concordo parcialmente	163	44	207	
		23,1%	21,3%	22,6%	
	Concordo plenamente	392	117	509	
		55,4%	56,5%	55,7%	
	Total	707	207	914	
		100,0%	100,0%	100,0%	
Recebeu informação prévia da Instituição sobre a importância do teste	Discordo plenamente	14	1	15	0,621
		2,0%	0,5%	1,6%	
	Discordo parcialmente	27	9	36	
		3,8%	4,3%	3,9%	
	Não concordo nem discordo	31	11	42	
		4,4%	5,3%	4,6%	
	Concordo parcialmente	106	30	136	
		15,0%	14,5%	14,9%	
	Concordo plenamente	530	156	686	
		74,9%	75,4%	75,0%	
	Total	708	207	915	
		100,0%	100,0%	100,0%	

Teste Qui-quadrado de Pearson; TP, teste de progresso; IES, instituição de ensino superior.

Tabela
Análise comparativa os itens sobre “pretende acessar o gabarito comentado” e “pretende acessar resultado” entre os alunos das duas IES.

Item	Respostas	IES			Valor P
		UNISA	FAMERP	Total	
Pretende acessar o gabarito comentado	Discordo plenamente	13	3	16	0,364
		1,8%	1,4%	1,7%	
	Discordo parcialmente	19	8	27	
		2,7%	3,9%	3,0%	
	Não concordo nem discordo	59	14	73	
		8,3%	6,8%	8,0%	
	Concordo parcialmente	75	31	106	
		10,6%	15,0%	11,6%	
	Concordo plenamente	542	151	693	
		76,6%	72,9%	75,7%	
	Total	708	207	915	
		100,0%	100,0%	100,0%	
Pretende acessar o resultado	Discordo plenamente	7	2	9	0,964
		1,0%	1,0%	1,0%	
	Discordo parcialmente	9	2	11	
		1,3%	1,0%	1,2%	
	Não concordo nem discordo	40	10	50	
		5,6%	4,8%	5,5%	
	Concordo parcialmente	63	21	84	
		8,9%	10,1%	9,2%	
	Concordo plenamente	589	172	761	
		83,2%	83,1%	83,2%	
	Total	708	207	915	
		100,0%	100,0%	100,0%	

Teste Qui-quadrado de Pearson; TP, teste de progresso; IES, instituição de ensino superior.

A seguir, seguem as comparações em relação ao aproveitamento dos resultados do TP pela IES, na percepção do aluno.

Como observa-se na tabela abaixo, houve diferença significante quanto às respostas, para o item "as questões são posteriormente discutidas na sala de aula". Apenas a categoria "discordo parcialmente" foi semelhante entre as duas IES, enquanto que todas as outras apresentaram diferença significante, como pode ser observado pela notação das letras "a" e "b". Uma proporção maior de alunos Famerp discordaram plenamente de que as questões são posteriormente discutidas na sala de aula, enquanto que os alunos da UNISA apresentaram uma proporção maior de posição neutra ou concordância, do que os alunos Famerp.

Quanto ao item "o conteúdo abordado na sua instituição é adequado para a realização do teste, houve diferença somente para as categorias "concordo parcialmente"(maior proporção de alunos Famerp) e "concordo plenamente" (maior proporção de alunos UNISA).

Tabela
Análise comparativa dos itens sobre o aproveitamento dos resultados do TP pela IES entre os alunos das duas IES.

Item	Respostas	IES			Valor P
		UNISA	FAMERP	Total	
As questões são posteriormente discutidas na sala de aula	Discordo plenamente	200_a	128_b	328	< **0,001***
		28,3%	62,4%	36,0%	
	Discordo parcialmente	105_a	22_a	127	
		14,9%	10,7%	13,9%	
	Não concordo nem discordo	231_a	47_b	278	
		32,7%	22,9%	30,5%	
	Concordo parcialmente	60_a	2_b	62	
		8,5%	1,0%	6,8%	
	Concordo plenamente	110_a	6_b	116	
		15,6%	2,9%	12,7%	
	Total	706	205	911	
		100,0%	100,0%	100,0%	
Importância da discussão das questões em sala de aula	Discordo plenamente	10	0	10	0,051†
		1,4%	0,0%	1,1%	
	Discordo parcialmente	16	1	17	
		2,3%	0,5%	1,9%	
	Não concordo nem discordo	48	14	62	
		6,8%	6,8%	6,8%	
	Concordo parcialmente	125	50	175	
		17,7%	24,3%	19,2%	
	Concordo plenamente	507	141	648	
		71,8%	68,4%	71,1%	
	Total	706	206	912	

		100,0%	100,0%	100,0%	
O conteúdo abordado em sua Instituição é adequado para a realização do teste	Discordo plenamente	8_a	0_a	8	**< 0,001***
		1,1%	0,0%	0,9%	
	Discordo parcialmente	49_a	13_a	62	
		7,0%	6,3%	6,8%	
	Não concordo nem discordo	122_a	45_a	167	
		17,3%	21,8%	18,4%	
	Concordo parcialmente	230_a	94_b	324	
		32,7%	45,6%	35,6%	
	Concordo plenamente	295_a	54_b	349	
		41,9%	26,2%	38,4%	
	Total	704	206	910	
		100,0%	100,0%	100,0%	

*Teste Qui-quadrado de Pearson; †Teste Exato de Fisher; TP, teste de progresso; IES, instituição de ensino superior.

A seguir, são mostradas as comparações para os itens sobre motivação e uso dos resultados do TP pelo próprio aluno para seu desenvolvimento acadêmico, entre os alunos das duas IES.

Como pode ser observado nas tabelas abaixo, houve diferença significante para 3 itens.

Para o item “motivado para fazer o teste”, houve diferença nas categorias “discordo parcialmente”, com uma proporção maior de alunos Famerp do que UNISA, posição neutra “não concordo nem discordo” maior também para alunos Famerp e “concordo plenamente” maior proporção de alunos UNISA.

Para o item “importância da realização do teste para o desenvolvimento acadêmico”, as categorias “discordo parcialmente” e “concordo parcialmente” apresentaram maior proporção de alunos Famerp; por outro lado, a categoria “concordo plenamente” foi escolhida pela maioria dos alunos UNISA (71,8%), com diferença significante em relação aos alunos Famerp (51%).

Para o item “leva em consideração o desempenho no teste para nortear os estudos”, a categoria “discordo parcialmente” apresentou maior proporção para alunos Famerp, enquanto a categoria “concordo plenamente” apresentou maior proporção para alunos UNISA.

Tabela
Análise comparativa dos itens sobre motivação e uso dos resultados do TP pelo próprio aluno para seu desenvolvimento acadêmico entre os alunos das duas IES.

Item	Respostas	IES			Valor P
		UNISA	FAMERP	Total	
Motivado para fazer o teste	Discordo plenamente	41_a	16_a	57	**< 0,001**
		5,8%	7,8%	6,3%	
	Discordo parcialmente	37_a	25_b	62	
		5,3%	12,1%	6,8%	
	Não concordo nem discordo	82_a	40_b	122	
		11,7%	19,4%	13,4%	
	Concordo parcialmente	179_a	58_a	237	
		25,5%	28,2%	26,1%	
	Concordo plenamente	364_a	67_b	431	
		51,8%	32,5%	47,4%	
	Total	703	206	909	
		100,0%	100,0%	100,0%	
Importância da realização do teste para o desenvolvimento acadêmico	Discordo plenamente	11_a	6_a	17	**< 0,001**
		1,6%	2,9%	1,9%	
	Discordo parcialmente	9_a	10_b	19	
		1,3%	4,9%	2,1%	

	Não concordo nem discordo	49_a	21_a	70	
		7,0%	10,2%	7,7%	
	Concordo parcialmente	129_a	64_b	193	
		18,4%	31,1%	21,3%	
	Concordo plenamente	504_a	105_b	609	
		71,8%	51,0%	67,1%	
	Total	702	206	908	
		100,0%	100,0%	100,0%	
Leva em conta o desenvolvimento no teste para avaliar o desenvolvimento acadêmico	Discordo plenamente	36	19	55	0,132
		8,0%	11,5%	8,9%	
	Discordo parcialmente	30	18	48	
		6,6%	10,9%	7,8%	
	Não concordo nem discordo	64	25	89	
		14,2%	15,2%	14,4%	
	Concordo parcialmente	140	51	191	
		31,0%	30,9%	31,0%	
	Concordo plenamente	182	52	234	
		40,3%	31,5%	37,9%	
	Total	452	165	617	
		100,0%	100,0%	100,0%	
Leva em conta a evolução do desempenho no teste para nortear os estudos	Discordo plenamente	49_a	24_a	73	**0,001**
		10,9%	14,5%	11,9%	
	Discordo parcialmente	31_a	25_b	56	
		6,9%	15,2%	9,1%	
	Não concordo nem discordo	82_a	31_a	113	
		18,2%	18,8%	18,3%	
	Concordo parcialmente	123_a	48_a	171	
		27,3%	29,1%	27,8%	
	Concordo plenamente	166_a	37_b	203	
		36,8%	22,4%	33,0%	
	Total	451	165	616	
		100,0%	100,0%	100,0%	

Teste Qui-quadrado de Pearson; TP, teste de progresso; IES, instituição de ensino superior.

6. Referências

1. BISHARA, A. J; HITTNER J.B. **Confidence intervals for correlations when data are not normal**. Behav Res 49, 294–309, 2017.

2. BONETT, D. G; WRIGHT, T. A. **Sample size requirements for estimating Pearson, Kendall and Spearman correlations**. Psychometrika, 65, 23–28, 2000.
3. BUSSAB, W.O.; MORETTIN, P.A. **Estatística Básica**. Saraiva, 9ª edição., São Paulo, 2017.
4. CONOVER, W.J. **Practical nonparametric statistics**. New York: John Wiley & Sons, 1999.
5. FIELD, A. **Descobrindo a Estatística usando o SPSS**. Tradução, consultoria e supervisão de Lorí Viali. Editora Artmed, 2009.
6. IBM Corporation. **IBM SPSS Statistics Algorithms**, 27.edição. IBM Corp.: Armonk, NY, USA, 2020.

7. MACDONALD, P. L.; GARDNER, R. C. **Type I error rate comparisons of post hoc procedures for I j Chi-Square tables**. Educational and psychological measurement, 60(5), 735-754, 2000.
8. R CORE TEAM. R: **A language and environment for statistical computing**. R Foundation for Statistical Computing, Vienna, Austria, 2015. URL https://www.R-project.org/
9. SIEGEL S.; CASTELLAN Jr NJ. **Estatística Não Paramétrica para Ciências do Comportamento**. Bookman, 2ª edição, São Paulo, 2006.
