# Supplementary material for: Continuous assessment in medical education: Exploring students’ views on the progress test
Source: PLoS One. 2024 Dec 19;19(12):e0314848. doi: 10.1371/journal.pone.0314848 (PMC11658631; doi:10.1371/journal.pone.0314848)
Supplement: S2 File — (PDF) [file pone.0314848.s003.pdf]

Relatório dos resultados do estudo:

O teste de progresso de quem realiza o mesmo: o discente

São José do Rio Preto, 18 de agosto de 2021

SUMÁRIO

I. Metodologia da análise estatística 3

1. Caracterização da amostra 3
2. Banco de dados 3
3. Análise estatística dos dados 3

II. Resultados ....

1. Análise descritiva da amostra 4
2. Análise descritiva das respostas às perguntas do instrumento aplicado 5
3. Análise de correlação 13

III. Referências 26

I - Metodologia da análise estatística

1. Caracterização da amostra

A amostra do estudo foi composta por 709 participantes.

2. Banco de dados

Os dados foram recebidos cadastrados no Excel. Posteriormente, foram importados para o software IBM-SPSS *Statistics* versão 27 (IBM Corporation, NY, USA) para análise exploratória dos dados e análise comparativa entre grupos.

3. Análise estatística dos dados

A análise exploratória dos dados incluiu as estatísticas descritivas, média, mediana, desvio-padrão, valor mínimo e valor máximo para variáveis numéricas e número e proporção para variáveis categóricas. Para análise do comportamento das variáveis contínuas, considerou-se as estatísticas descritivas, gráficos de histograma e boxplot e o teste específico para o pressuposto teórico de normalidade Kolmogorov-Smirnov (CONOVER, 1999). A análise de correlação de Spearman foi realizada para verificar a correlação entre as variáveis discretas e ordinais (semestre da graduação e item do questionário); o resultado foi demostrando pelo coeficiente de correlação de Spearman e seu respectivo intervalo de confiança de 95% (BONETT & WRIGHT, T. A, 2000; SIEGEL & CASTELLAN, 2006; BISHARA & HITTNER, 2017). Análise estatística foi realizada mediante os softwares IBM-SPSS *Statistics* versão 27 (IBM Corporation, NY, USA).

II - Resultados

1. Análise descritiva da amostra

A Tabela abaixo demonstra a faixa etária e semestre da graduação, entre os 709 alunos que participaram da pesquisa.

Tabela
Faixa etária e semestre de graduação entre os 709 participantes incluídos no estudo.

Variável	N = 709
Idade, n (%)	
17 a 20 anos	263 (37,1)
21 a 25 anos	350 (49,3)
26 a 30 anos	67 (9,4)
31 a 35 anos	16 (2,3)
36 a 39 anos	4 (0,6)
Acima de 40 anos	9 (1,3)
Semestre de graduação, n (%)	
1º. Semestre	149 (21,0)
2º. Semestre	111 (15,7)
3º. Semestre	44 (6,2)
4º. Semestre	104 (14,5)
5º. Semestre	63 (8,9)
6º. Semestre	36 (5,1)
7º. Semestre	36 (5,1)
8º. Semestre	31 (4,4)
9º. Semestre	38 (5,4)
10º. Semestre	23 (3,2)
11º. Semestre	31 (4,4)
12º. Semestre	43 (6,1)

Variáveis categóricas estão descritas em número (porcentagem)

2. Análise descritiva das respostas às perguntas do instrumento aplicado

A seguir, a Tabela abaixo demostra a distribuição das respostas dos alunos aos itens aplicados, em relação à autopercepção do aluno no desempenho esperado no TP.

Tabela
Autopercepção do aluno no desempenho esperado no Teste de Progresso.

Itens	N (%)
Porcentagem de questões que espera acertar, n (%)	
0 a 20%	188 (26,5)
20 a 40%	216 (30,5)
40 a 60%	170 (24)
60 a 80%	117 (16,5)
80 a 100%	18 (2,5)
Área que acredita ter melhor desempenho opção 1, n (%)	
Básica	255 (36)
Clínica	127 (17,9)
Cirurgia	74 (10,4)
GO	78 (11)
Pediatria	41 (5,8)
Saúde coletiva	134 (18,9)
Área que acredita ter melhor desempenho opção 2; n (%)	
Cirurgia	1 (0,1)
Pediatria	2 (0,3)
Saúde coletiva	1 (0,1)
Não respondeu	705 (99,5)

A Tabela abaixo demostra a distribuição das respostas dos alunos aos itens, em relação à adequação da construção do TP e possíveis movimentos institucionais para promover a adesão do acadêmico na realização do mesmo.

Tabela
Adequação da construção do TP e possíveis movimentos institucionais para promover a adesão do acadêmico na realização do mesmo.

Itens	N (%)
Os enunciados das questões e as alternativas são claras para responder, n (%)	
Discordo plenamente	2 (0,3)
Discordo parcialmente	22 (3,1)
Não concordo nem discordo	90 (12,7)
Concordo parcialmente	248 (35)
Concordo plenamente	341 (48,1)
Não respondeu	6 (0,8)
Tempo de realização é adequado para o conteúdo, n (%)	
Discordo plenamente	23 (3,2)
Discordo parcialmente	51 (7,2)
Não concordo nem discordo	78 (11)
Concordo parcialmente	163 (23)
Concordo plenamente	392 (55,3)
Não respondeu	2 (0,3)
Recebeu informação prévia da Instituição sobre a importância do teste, n (%)	
Discordo plenamente	14 (2)
Discordo parcialmente	27 (3,7)
Não concordo nem discordo	31 (4,4)
Concordo parcialmente	106 (15)
Concordo plenamente	530 (74,8)
Não respondeu	1 (0,1)

A Tabela abaixo mostra a distribuição das respostas dos alunos aos itens sobre se pretende acessar o gabarito comentado e resultado do TP.

Tabela
Itens sobre se pretende acessar o gabarito comentado e o resultado do TP.

Variáveis	N (%)
Pretende acessar o gabarito comentado, n (%)	
Discordo plenamente	13 (1,8)
Discordo parcialmente	19 (2,7)
Não concordo nem discordo	59 (8,3)
Concordo parcialmente	75 (10,6)
Concordo plenamente	542 (76,5)
Não respondeu	1 (0,1)
Pretende acessar o resultado, n (%)	
Discordo plenamente	7 (1)
Discordo parcialmente	9 (1,3)
Não concordo nem discordo	40 (5,6)
Concordo parcialmente	63 (8,9)
Concordo plenamente	589 (83,1)
Não respondeu	1 (0,1)

A Tabela abaixo mostra a distribuição das respostas dos alunos quanto ao aproveitamento dos resultados do TP pela IES.

Tabela
Aproveitamento dos resultados do TP pela IES.

Itens	N (%)
As questões são posteriormente discutidas na sala de aula, n (%)	
Discordo plenamente	200 (28,2)
Discordo parcialmente	105 (14,8)
Não concordo nem discordo	231 (32,6)
Concordo parcialmente	60 (8,5)
Concordo plenamente	110 (15,5)
Não respondeu	3 (0,4)
Importância da discussão das questões em sala de aula, n (%)	
Discordo plenamente	10 (1,4)
Discordo parcialmente	16 (2,3)
Não concordo nem discordo	48 (6,8)
Concordo parcialmente	125 (17,6)
Concordo plenamente	507 (71,5)
Não respondeu	3 (0,4)
O conteúdo abordado em sua Instituição é adequado para a realização do teste, n (%)	
Discordo plenamente	8 (1,1)
Discordo parcialmente	49 (6,9)
Não concordo nem discordo	122 (17,2)
Concordo parcialmente	230 (32,4)
Concordo plenamente	295 (41,7)
Não respondeu	5 (0,7)

TP, Teste de Progresso; IES, Instituição de Ensino Superior.

A Tabela abaixo demonstra a distribuição das respostas dos alunos aos itens que caracterizam a motivação e uso dos resultados do TP pelo próprio aluno para seu desenvolvimento acadêmico.

Tabela
Motivação e uso dos resultados do TP pelo próprio aluno para seu desenvolvimento acadêmico

Itens	N (%)
Motivado para fazer o teste, n (%)	
Discordo plenamente	41 (5,8)
Discordo parcialmente	37 (5,2)
Não concordo nem discordo	82 (11,6)
Concordo parcialmente	179 (25,3)
Concordo plenamente	364 (51,3)
Não respondeu	6 (0,8)
Importância da realização do teste para o desenvolvimento acadêmico, n (%)	
Discordo plenamente	11 (1,6)
Discordo parcialmente	9 (1,3)
Não concordo nem discordo	49 (6,9)
Concordo parcialmente	129 (18,1)
Concordo plenamente	504 (71,1)
Não respondeu	7 (1)
Leva em conta o desenvolvimento no teste para avaliar o desenvolvimento acadêmico, n (%)	
Discordo plenamente	36 (5,1)
Discordo parcialmente	30 (4,2)
Não concordo nem discordo	64 (9)
Concordo parcialmente	140 (19,7)
Concordo plenamente	182 (25,7)
Não respondeu	257 (36,3)
Leva em conta a evolução do desempenho no teste para nortear os estudos, n (%)	
Discordo plenamente	49 (6,9)
Discordo parcialmente	31 (4,4)
Não concordo nem discordo	82 (11,6)
Concordo parcialmente	123 (17,3)
Concordo plenamente	166 (23,4)
Não respondeu	258 (36,4)

Variáveis categóricas estão descritas em número (porcentagem).

Resultados da autopercepção do aluno no desempenho esperado no TP, segundo o semestre cursado.

Tabela
Distribuição das respostas ao item "Porcentagem de questões que espera acertar", segundo o semestre de graduação cursado.

Série de graduação	Porcentagem de questões que espera acertar	N (%)
1º. Semestre		
	0 a 20%	83/149 (55,8)
	20 a 40%	54/149 (36,2)
	40 a 60%	8/149 (5,4)
	60 a 80%	2/149 (1,3)
	80 a 100%	2/149 (1,3)
2º. Semestre		
	0 a 20%	74/111 (66,7)
	20 a 40%	32/111 (28,8)
	40 a 60%	2/111 (1,8)
	80 a 100%	3/111 (2,7)
3º. Semestre		
	0 a 20%	14/44 (31,8)
	20 a 40%	26/44 (59,1)
	40 a 60%	3/44 (6,8)
	80 a 100%	1/44 (2,3)
4º. Semestre		
	0 a 20%	14/104 (13,5)
	20 a 40%	64/104 (61,5)
	40 a 60%	23/104 (22,1)
	60 a 80%	3/104 (2,9)
5º. Semestre		
	0 a 20%	2/63 (3,2)
	20 a 40%	28/63 (44,4)
	40 a 60%	26/63 (41,3)
	60 a 80%	5/63 (7,9)
	80 a 100%	2/63 (3,2)
6º. Semestre		
	20 a 40%	4/36 (11,1)
	40 a 60%	22/36 (61,1)
	60 a 80%	8/36 (22,2)
	80 a 100%	2/36 (5,6)
7º. Semestre		
	20 a 40%	6/36 (16,7)
	40 a 60%	20/36 (55,5)
	60 a 80%	9/36 (25)
	80 a 100%	1/36 (2,8)
8º. Semestre		
	40 a 60%	17/31 (54,8)

	60 a 80%	14/31 (45,2)
9º. Semestre		
	20 a 40%	1/38 (2,6)
	40 a 60%	16/38 (42,1)
	60 a 80%	20/38 (52,7)
	80 a 100%	1/38 (2,6)
10º. Semestre		
	0 a 20%	1/23 (4,3)
	40 a 60%	11/23 (47,9)
	60 a 80%	10/23 (43,5)
	80 a 100%	1/23 (4,3)
11º. Semestre		
	20 a 40%	1/31 (3,2)
	40 a 60%	11/31 (35,5)
	60 a 80%	17/31 (54,8)
	80 a 100%	2/31 (6,5)
12º. Semestre		
	40 a 60%	11/43 (25,6)
	60 a 80%	29/43 (67,4)
	80 a 100%	3/43 (7,0)

A seguir os semestres foram agrupados de 2 em 2, para resultar em “ano da graduação”.
A distribuição das respostas dos alunos para o item “Porcentagem de questões que espera acertar”, segundo o ano da graduação, pode ser vista na Tabela abaixo.

Tabela
Distribuição das respostas ao item “Porcentagem de questões que espera acertar”, segundo o ano de graduação.

	Ano de graduação					
	1ºAno	2ºAno	3ºAno	4ºAno	5ºAno	6ºAno
Porcentagem de questões que espera acertar	N = 260	N = 148	N = 99	N = 67	N = 61	N = 74
0 a 20%	157 (60,4)	28 (18,9)	2 (2,0)	0 (0,0)	1 (1,6)	0 (0,0)
20 a 40%	86 (33,1)	90 (60,8)	32 (32,4)	6 (9,0)	1 (1,6)	1 (1,4)
40 a 60%	10 (3,8)	26 (17,6)	48 (48,6)	37 (55,2)	27 (44,3)	22 (29,7)
60 a 80%	2 (0,8)	3 (2,0)	13 (13,1)	23 (34,3)	30 (49,2)	46 (62,1)
80 a 100%	5 (1,9)	1 (0,7)	4 (4,0)	1 (1,5)	2 (3,3)	5 (6,8)

1ºAno = 1º. semestre e 2º. semestre; 2ºAno = 3º. semestre e 4º. semestre; 3ºAno = 5º. semestre e 6º. semestre; 4ºAno = 7º. semestre e 8º. semestre; 5ºAno = 9º. semestre e 10º. semestre; 6ºAno = 11º. semestre e 12º. Semestre.

3. Análise de correlação

A análise de correlação é adequada quando se estuda a relação entre duas variáveis que tenham natureza numérica ou ordinal. O coeficiente de correlação é mensurado por meio da escala de valores +1 a -1; quando o valor está próximo de +1, assume-se a correlação linear positiva perfeita (ou seja, quanto maior o valor de uma variável, maior também será o valor da outra variável), e quando o valor do coeficiente está próximo de -1, assume-se a correlação linear negativa perfeita (ou seja, quanto maior o valor de uma variável, menor será o valor da outra); os valores próximos de zero indicam a ausência de correlação. A força da correlação entre duas variáveis pode ser interpretada da seguinte forma, segundo a literatura: r |≤0,25| = ausência de correlação; |0,26 – 0,50| = correlação fraca; |0,51 – 0,75| = correlação moderada e |>0,75| = correlação forte. O resultado do "r" é aquele encontrado na amostra. Por outro lado, o IC 95% (intervalo de confiança) mostra os valores de r extrapolados para a população, com confiança de 95%. Além disso, a visualização por gráficos de dispersão pode auxiliar na interpretação de existência ou não de correlação, pela forma como os valores dos eixos X e Y se relacionam.

Houve correlação positiva forte entre a série da graduação e a percepção do aluno sobre a porcentagem de questões que espera acertar, ou seja, conforme aumenta a série da graduação, maior foi a porcentagem de questões que o aluno espera acertar. A tabela abaixo demonstra o valor do coeficiente de correlação (r_S) e o seu intervalo de confiança. Da mesma forma, podemos visualizar a correlação entre as duas variáveis, através do gráfico de dispersão.

Tabela
Correlação entre série de graduação e o item "Porcentagem de questões que espera acertar".

	Série da graduação	
Item	r_S	IC (95%)
Porcentagem de questões que espera acertar	0,752	0,713; 0,786

r_S = coeficiente de correlação de Spearman; IC, intervalo de confiança.

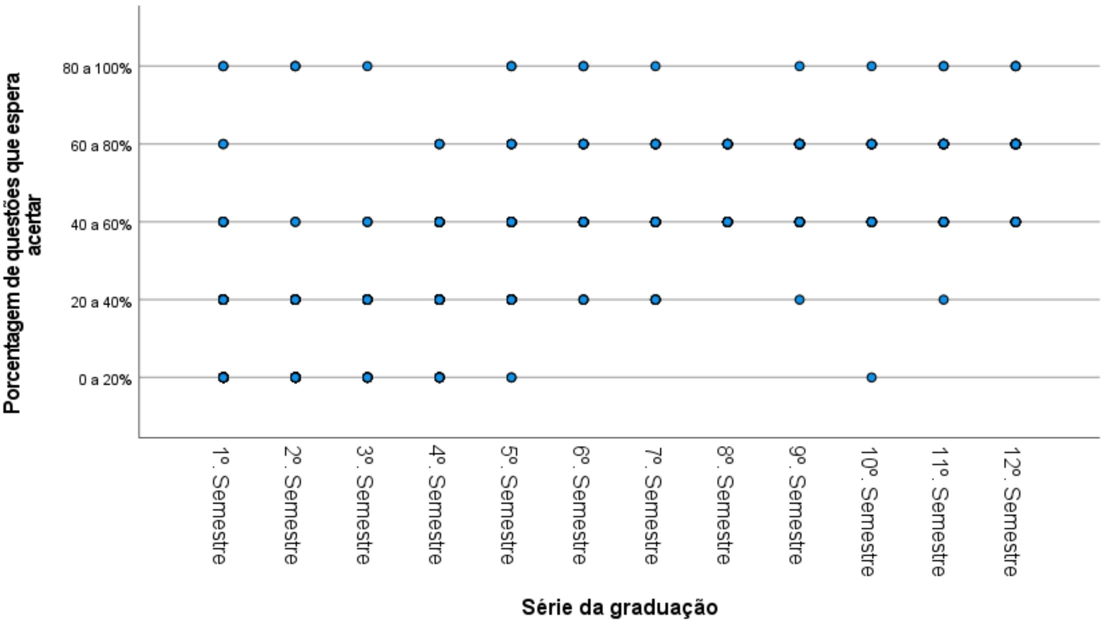

FIGURA

Gráfico de dispersão entre o item "Porcentagem de questões que espera acertar" e série da graduação.

A seguir, é apresentada a análise de correlação entre a série da graduação (12 semestres) e as respostas a cada um dos 12 itens do instrumento, que permitiam resposta em escala tipo Likert.

Na Tabela abaixo, podemos observar que não houve correlação entre as respostas acerca da adequação da construção do TP e possíveis movimentos institucionais para promover a adesão do acadêmico na realização do mesmo e a série da graduação. Portanto, a percepção do aluno quanto a estes itens foi independente do semestre cursado. Isto está ilustrado nos respectivos gráficos de dispersão.

Tabela
Correlação entre a série de graduação e as respostas sobre adequação da construção do TP e possíveis movimentos institucionais para promover a adesão do acadêmico na realização do mesmo *.

	Série de graduação	
Item	r_s	IC (95%)
Os enunciados das questões e as alternativas são claras para responder	-0,033	-0,107; 0,041
Tempo de realização é adequado para o conteúdo	0,153	0,080; 0,225
Recebeu informação prévia da Instituição sobre a importância do teste	-0,051	-0,124; 0,023

r_s = coeficiente de correlação de Spearman; IC, intervalo de confiança.
*As respostas às perguntas do instrumento foram relatadas através de uma escala tipo Likert, onde: 0 = discordo plenamente; 1 = discordo parcialmente; 2 = não concordo e nem discordo; 3 = concordo parcialmente; 4 = concordo plenamente.

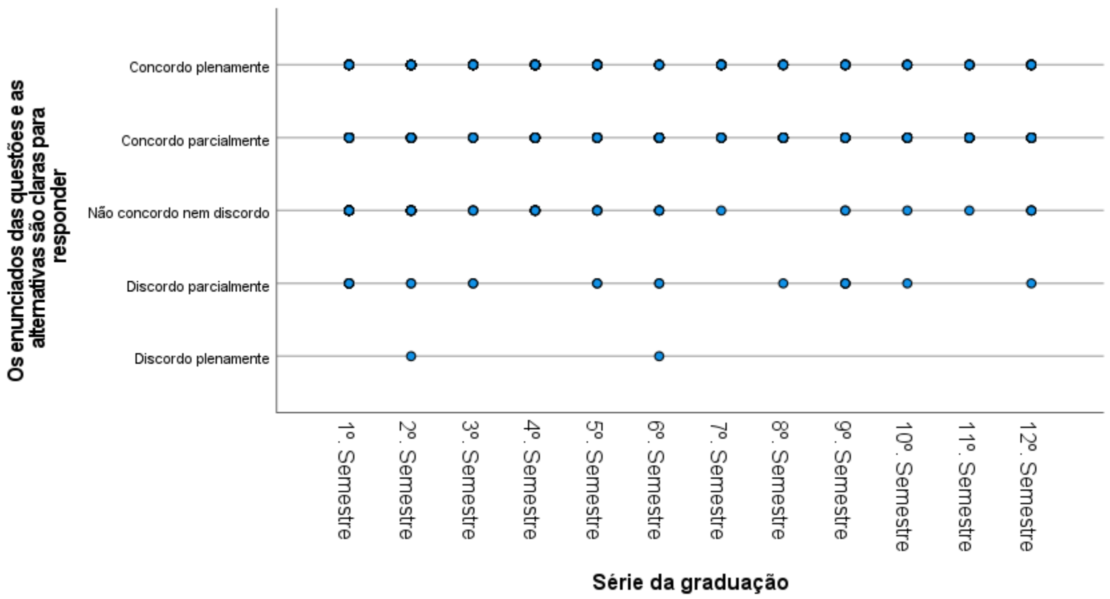

FIGURA

Gráfico de dispersão entre o item “Os enunciados das questões e as alternativas são claras para responder” e série da graduação.

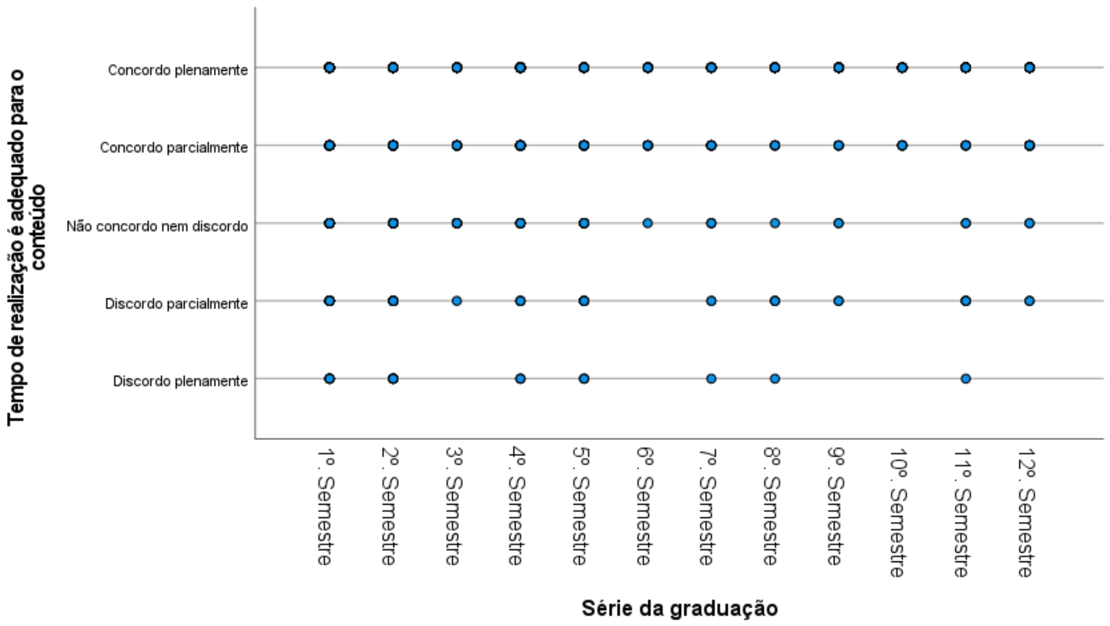

FIGURA

Gráfico de dispersão entre o item “O tempo de realização é adequado para o conteúdo” e série da graduação.

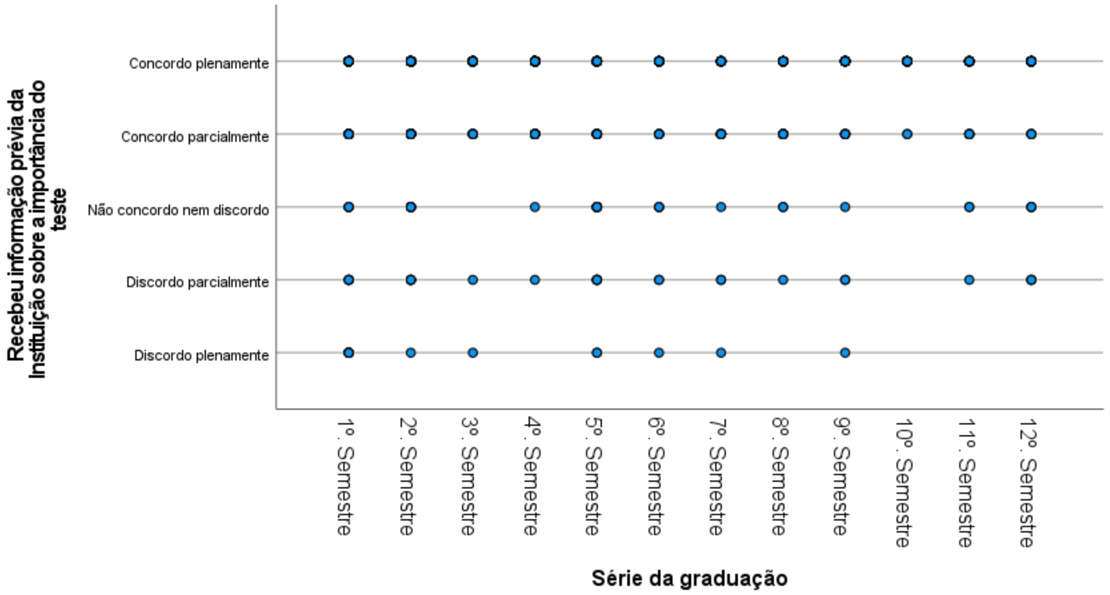

FIGURA

Gráfico de dispersão entre o item "Recebeu informação prévia da Instituição sobre a importância do teste" e série da graduação.

Na Tabela abaixo, podemos observar a correlação entre as respostas sobre se pretende acessar o gabarito comentado e resultado do TP e a série da graduação. Quanto ao item “Pretende acessar o gabarito comentado”, podemos observar que houve uma fraca correlação positiva (observar o IC 95%), ou seja, conforme avançamos no semestre, as pontuações mais baixas foram desaparecendo e permanecem as pontuações mais altas de resposta (observar o gráfico de dispersão). Por outro lado, para o item “Pretende acessar o resultado” considera-se que não houve correlação.

Tabela
Correlação entre a série de graduação e as respostas sobre se pretende acessar o gabarito comentado e resultado do TP*

	Série de graduação	
Item	r_s	IC (95%)
Pretende acessar o gabarito comentado	0,186	0,113; 0,257
Pretende acessar o resultado	0,106	0,032; 0,178

r_s = coeficiente de correlação de Spearman; IC, intervalo de confiança.
*As respostas às perguntas do instrumento foram relatadas através de uma escala tipo Likert, onde: 0 = discordo plenamente; 1 = discordo parcialmente; 2 = não concordo e nem discordo; 3 = concordo parcialmente; 4 = concordo plenamente.

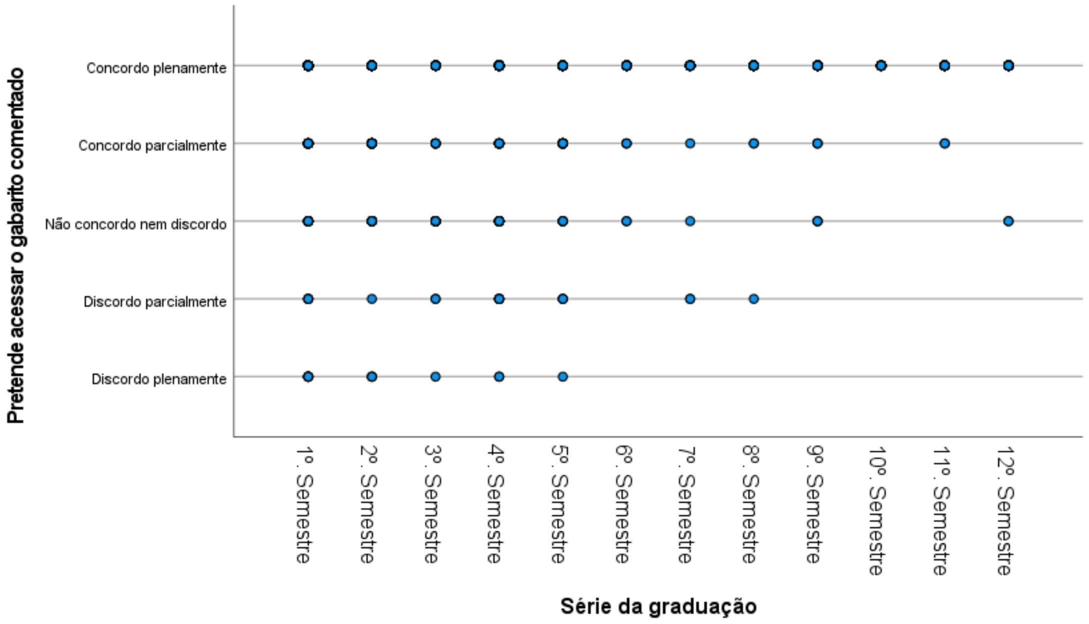

FIGURA

Gráfico de dispersão entre o item “Pretende acessar o gabarito comentado” e série da graduação.

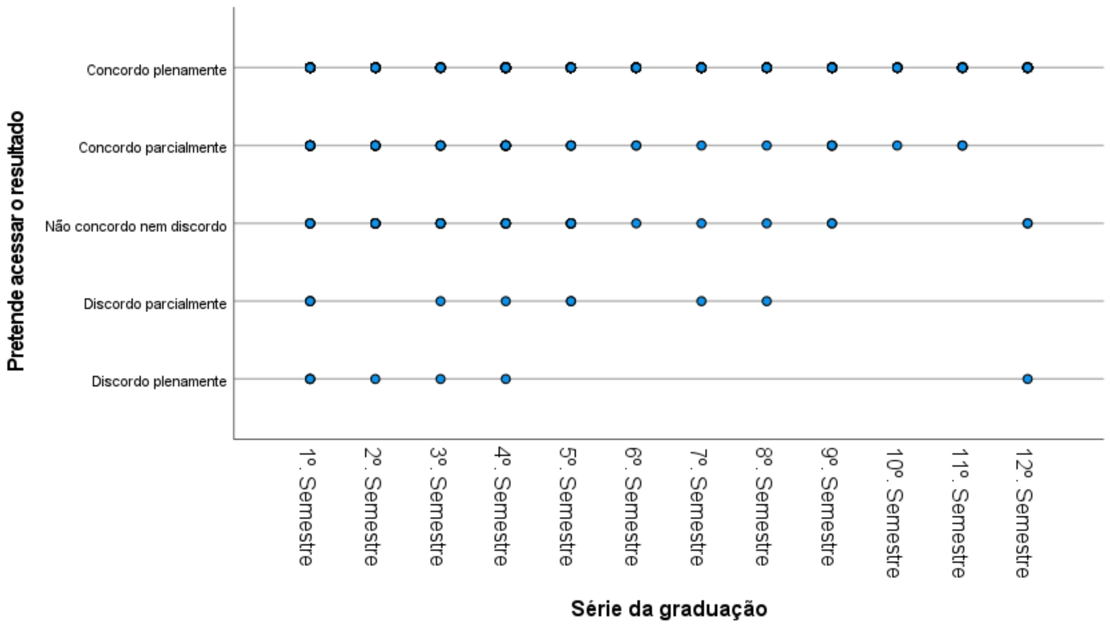

FIGURA

Gráfico de dispersão entre o item “Pretende acessar o resultado” e série da graduação.

Na Tabela abaixo, podemos observar a correlação entre as respostas quanto ao aproveitamento dos resultados do TP pela IES e a série da graduação. O coeficiente de correlação para o item “As questões são posteriormente discutidas na sala de aula” demonstrou fraca correlação positiva (confirmada pelo intervalo de confiança de 95%), ou seja, conforme mais avançado o semestre, maior a pontuação das respostas em escala Likert.

A percepção do aluno quanto às demais questões foi independente do semestre cursado, como verificado pelo coeficiente de correlação. Observar os respectivos gráficos de dispersão.

Tabela
Correlação entre a série de graduação e as respostas quanto ao aproveitamento dos resultados do TP pela IES*.

	Série de graduação	
Item	r_s	IC (95%)
As questões são posteriormente discutidas na sala de aula	0,314	0,244; 0,381
Importância da discussão das questões em sala de aula	0,141	0,068; 0,231
O conteúdo abordado em sua Instituição é adequado para a realização do teste	-0,202	-0,273; -0,129

r_s = coeficiente de correlação de Spearman; IC, intervalo de confiança.
*As respostas às perguntas do instrumento foram relatadas através de uma escala tipo Likert, onde: 0 = discordo plenamente; 1 = discordo parcialmente; 2 = não concordo e nem discordo; 3 = concordo parcialmente; 4 = concordo plenamente.

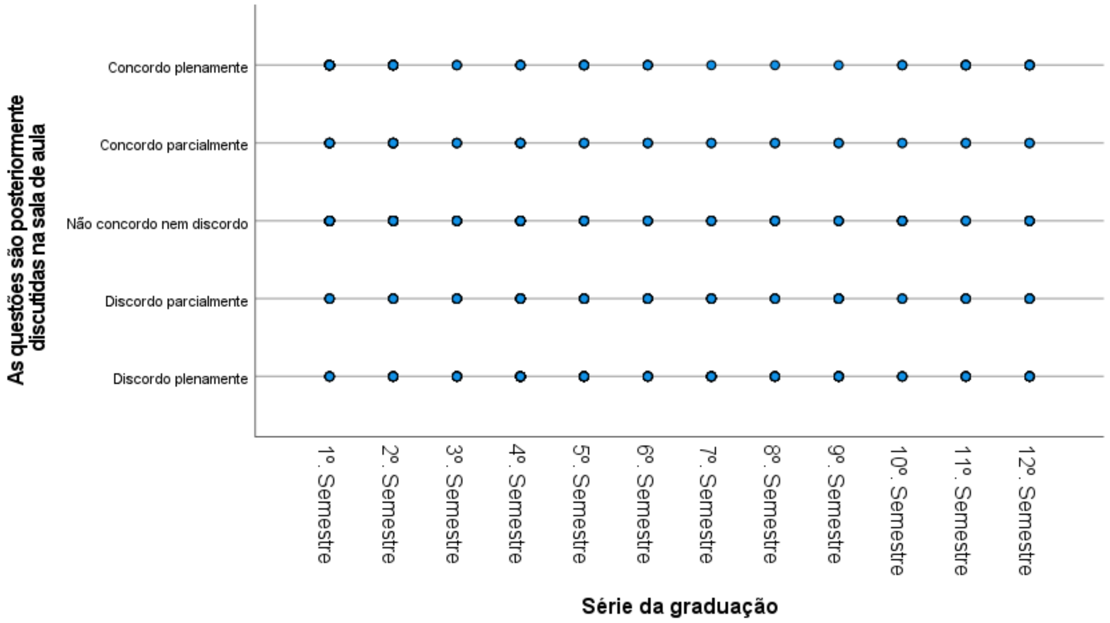

FIGURA

Gráfico de dispersão entre o item "As questões são posteriormente discutidas na sala de aula" e série da graduação.

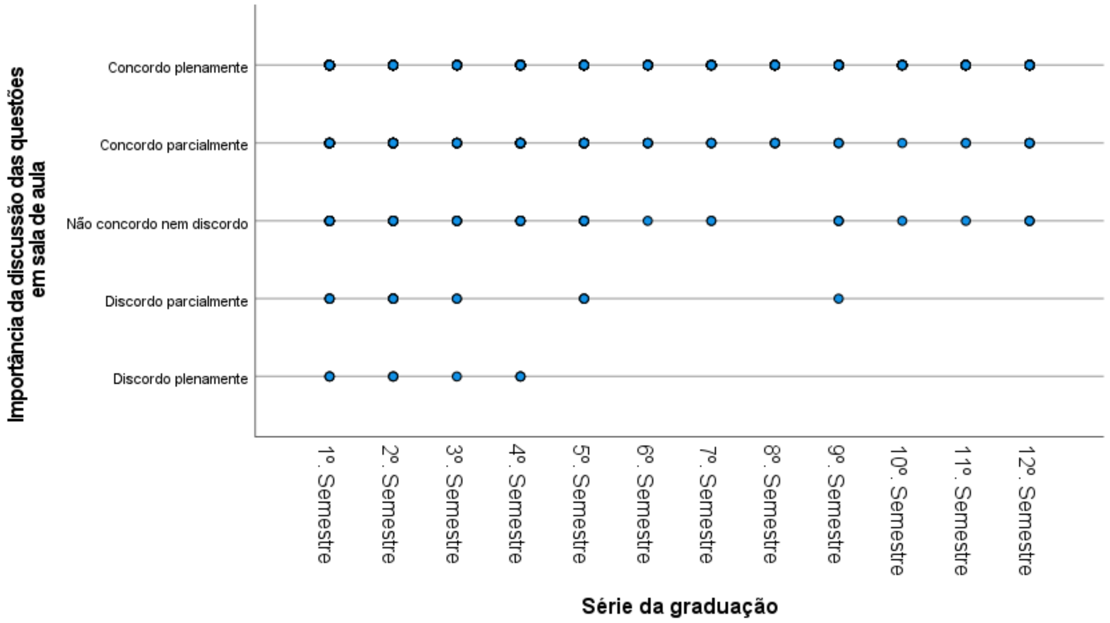

FIGURA

Gráfico de dispersão entre o item "Importância da discussão das questões em sala de aula" e série da graduação.

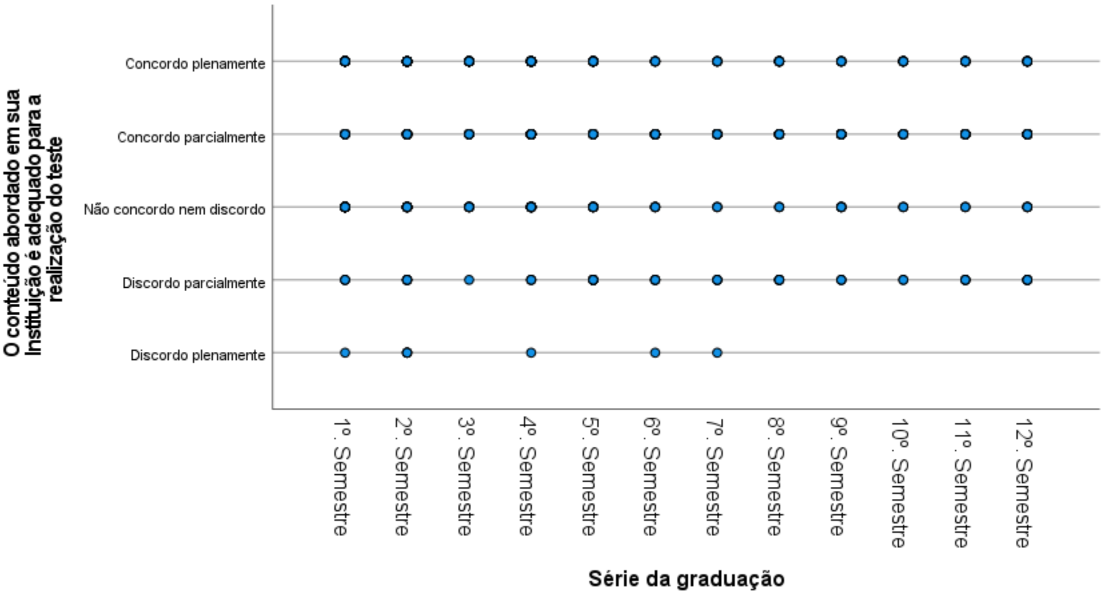

FIGURA

Gráfico de dispersão entre o item "O conteúdo abordado em sua Instituição é adequado para a realização do teste" e série da graduação.

Na Tabela abaixo, podemos observar que não houve correlação entre as respostas acerca da motivação e uso dos resultados do TP pelo próprio aluno para seu desenvolvimento acadêmico e a série da graduação. Portanto, a percepção do aluno quanto a estas questões foi independente do semestre cursado. Isto está ilustrado nos respectivos gráficos de dispersão.

Tabela
Correlação entre a série de graduação e as respostas quanto à motivação e uso dos resultados do TP pelo próprio aluno para seu desenvolvimento acadêmico *.

	Série de graduação	
Item	r_s	IC (95%)
Motivado para fazer o teste	-0,082	-0,155; -0,008
Importância da realização do teste para o desenvolvimento acadêmico	-0,129	-0,201; -0,055
Leva em conta o desenvolvimento no teste para avaliar o desenvolvimento acadêmico	-0,103	-0,194; -0,011
Leva em conta a evolução do desempenho no teste para nortear os estudos	0,127	0,035; 0,217

r_s = coeficiente de correlação de Spearman; IC, intervalo de confiança.
*As respostas às perguntas do instrumento foram relatadas através de uma escala tipo Likert, onde: 0 = discordo plenamente; 1 = discordo parcialmente; 2 = não concordo e nem discordo; 3 = concordo parcialmente; 4 = concordo plenamente.

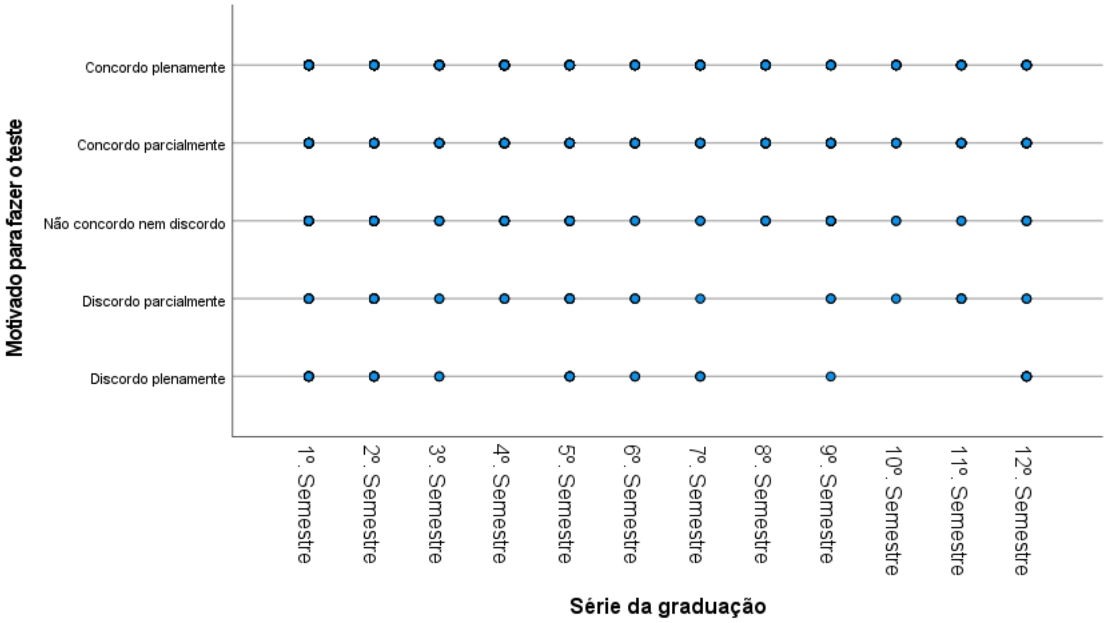

FIGURA

Gráfico de dispersão entre o item “Motivado para fazer o teste” e série da graduação.

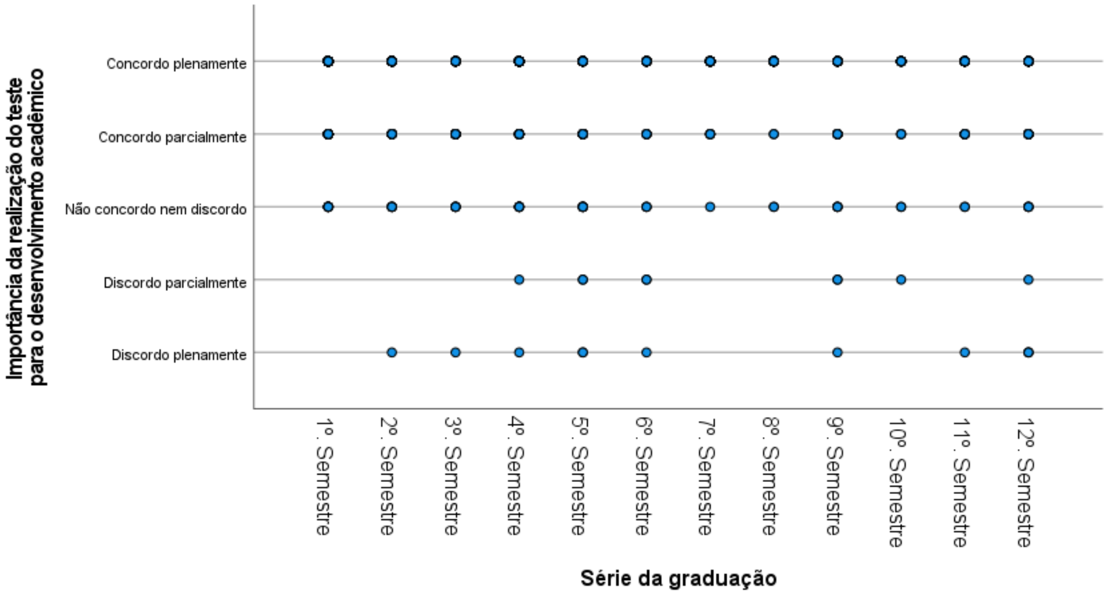

FIGURA

Gráfico de dispersão entre o item “Importância da realização do teste para o desenvolvimento acadêmico” e série da graduação.

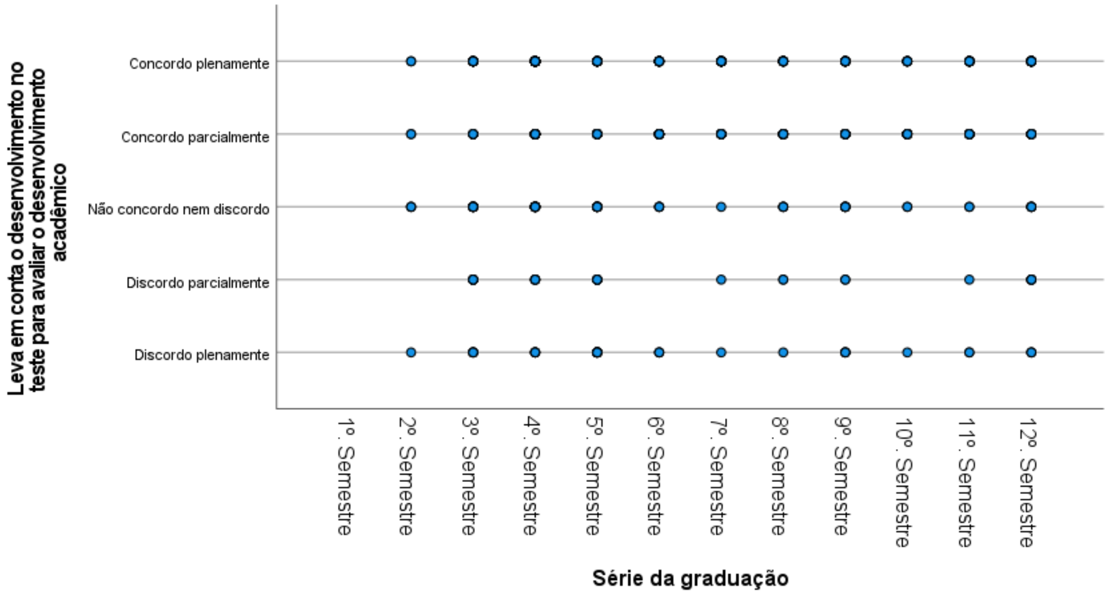

FIGURA

Gráfico de dispersão entre o item "Leva em conta o desenvolvimento no teste para avaliar o desenvolvimento acadêmico" e série da graduação.

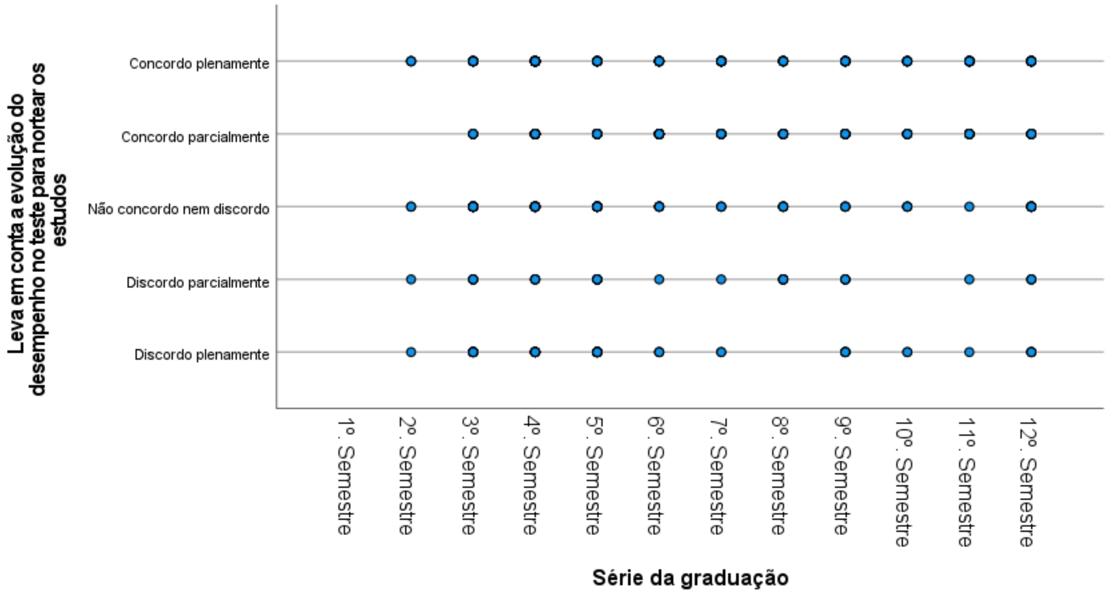

FIGURA

Gráfico de dispersão entre o item "Leva em conta a evolução do desempenho no teste para nortear os estudos" e série da graduação.

4. Referências

1. CONOVER, W.J. **Practical nonparametric statistics**. New York: John Wiley & Sons, 1999.
2. SIEGEL S.; CASTELLAN Jr NJ. **Estatística Não Paramétrica para Ciências do Comportamento**. Bookman, 2ª edição, São Paulo, 2006.
3. BONETT, D. G; WRIGHT, T. A. **Sample size requirements for estimating Pearson, Kendall and Spearman correlations**. Psychometrika, 65, 23–28, 2000.
4. BISHARA, A. J; HITTNER J.B. **Confidence intervals for correlations when data are not normal**. Behav Res 49, 294–309, 2017.
