# Supplementary material for: Continuous assessment in medical education: Exploring students’ views on the progress test
Source: PLoS One. 2024 Dec 19;19(12):e0314848. doi: 10.1371/journal.pone.0314848 (PMC11658631; doi:10.1371/journal.pone.0314848)
Supplement: S3 File — (PDF) [file pone.0314848.s004.pdf]

Machine Translated by Google

Report of study results:

The progress test of whoever performs it: the student

August 18, 2021

Machine Translated by Google

SUMMARY

I. Methodology of statistical analysis 3

1. Sample characterization 3
2. Database 3
3. Statistical analysis of data 3

II. Results ....

1. Descriptive analysis of the sample 4
2. Descriptive analysis of the responses to the instrument's questions applied 5
3. Correlation analysis 13

III. References 26

Machine Translated by Google

I - Methodology of statistical analysis

1. Sample characterization

The study sample consisted of 709 participants.

2. Database

The data was received registered in Excel. Later, it was imported into IBM-SPSS *Statistics* version 27 software (IBM Corporation, NY, USA) for exploratory data analysis and comparative analysis between groups.

3. Statistical analysis of data

Exploratory data analysis included descriptive statistics, mean, median, standard deviation, minimum value and maximum value for numerical variables and number and proportion for categorical variables. To analyze the behavior of continuous variables, descriptive statistics, histogram graphs and boxplot and the specific test for the theoretical assumption of Kolmogorov- normality Smirnov (CONOVER, 1999). Spearman's correlation analysis was performed to verify the correlation between discrete and ordinal variables (undergraduate semester and item of the questionnaire); the result was demonstrated by Spearman's correlation coefficient and its respective 95% confidence interval (BONETT & WRIGHT, T. A, 2000; SIEGEL & CASTELLAN, 2006; BISHARA & HITTNER, 2017). Statistical analysis was performed using IBM-SPSS *Statistics* software version 27 (IBM Corporation, NY, USA).

Machine Translated by Google

II - Results

1. Descriptive analysis of the sample

The table below shows the age range and semester of graduation among the 709 students who participated in the survey.

Table

Age range and semester of graduation among the 709 participants included in the study.

Variable	N = 709
Age, n (%)	
17 to 20 years	263 (37.1)
21 to 25 years	350 (49.3)
26 to 30 years	67 (9.4)
31 to 35 years	16 (2.3)
36 to 39 years old	4 (0.6)
Over 40 years old	9 (1.3)
Undergraduate semester, n (%)	
1st Semester	149 (21.0)
2nd Semester	111 (15.7)
3rd Semester	44 (6.2)
4th Semester	104 (14.5)
5th Semester	63 (8.9)
6th Semester	36 (5.1)
7th Semester	36 (5.1)
8th Semester	31 (4.4)
9th Semester	38 (5.4)
10th Semester	23 (3.2)
11th Semester	31 (4.4)
12th Semester	43 (6.1)

Categorical variables are described in number (percentage)

Machine Translated by Google

2. Descriptive analysis of the responses to the questions in the instrument applied

Next, the Table below shows the distribution of students' responses to the items applied, in relation to the student's self-perception of the expected performance in the TP.

Table

Student self-perception of expected performance in the Progress Test.

Items	N (%)
Percentage of questions you expect to get right, n (%)	
0 to 20%	188 (26.5)
20 to 40%	216 (30.5)
40 to 60%	170 (24)
60 to 80%	117 (16.5)
80 to 100%	18 (2.5)
Area believed to have better performance option 1, n (%)	
Basic	255 (36)
Clinic	127 (17.9)
Surgery	74 (10.4)
GO	78 (11)
Pediatrics	41 (5.8)
Public health	134 (18.9)
Area believed to have better performance option 2; n (%)	
Surgery	1 (0.1)
Pediatrics	2 (0.3)
Collective health	1 (0.1)
Did not respond	705 (99.5)

Machine Translated by Google

The Table below shows the distribution of students' responses to the items, in relation to adequacy of the TP construction and possible institutional movements to promote the academic's adherence to carrying it out.

Table

Adequacy of the construction of the TP and possible institutional movements to promote academic participation in its implementation.

Items	N (%)
The questions and alternatives are clear to answer, n (%)	
I completely disagree	2 (0.3)
I partially disagree	22 (3.1)
I neither agree nor disagree	90 (12.7)
I partially agree	248 (35)
I completely agree	341 (48.1)
Did not respond	6 (0.8)
Completion time is adequate for the content, n (%)	
I completely disagree	23 (3.2)
I partially disagree	51 (7.2)
I neither agree nor disagree	78 (11)
I partially agree	163 (23)
I completely agree	392 (55.3)
Did not respond	2 (0.3)
Received prior information from the Institution about the importance of the test, n (%)	
I completely disagree	14 (2)
I partially disagree	27 (3.7)
I neither agree nor disagree	31 (4.4)
I partially agree	106 (15)
I completely agree	530 (74.8)
Did not respond	1 (0.1)

Machine Translated by Google

The Table below shows the distribution of students' responses to the items about whether they intend to access the commented template and TP result.

Table

Items about whether you want to access the commented answer sheet and the TP result.

Variables	N (%)
Do you want to access the commented template, n (%)	
I completely disagree	13 (1.8)
I partially disagree	19 (2.7)
I neither agree nor disagree	59 (8.3)
I partially agree	75 (10.6)
I completely agree	542 (76.5)
Did not respond	1 (0.1)
Do you want to access the result, n (%)	
I completely disagree	7 (1)
I partially disagree	9 (1.3)
I neither agree nor disagree	40 (5.6)
I partially agree	63 (8.9)
I completely agree	589 (83.1)
Did not respond	1 (0.1)

The Table below shows the distribution of students' responses regarding achievement of the TP results by the IES.

Table

Use of TP results by the HEI.

Items	N (%)
The issues are then discussed in the classroom, n (%)	
I completely disagree	200 (28.2)
I partially disagree	105 (14.8)
I neither agree nor disagree	231 (32.6)
I partially agree	60 (8.5)
I completely agree	110 (15.5)
Did not respond	3 (0.4)
Importance of discussing issues in class, n (%)	
I completely disagree	10 (1.4)
I partially disagree	16 (2.3)
I neither agree nor disagree	48 (6.8)
I partially agree	125 (17.6)
I completely agree	507 (71.5)
Did not respond	3 (0.4)
The content covered in your Institution is suitable for taking the test, n (%)	
I completely disagree	8 (1.1)
I partially disagree	49 (6.9)
I neither agree nor disagree	122 (17.2)
I partially agree 230 (32.4)	
I completely agree 295 (41.7)	
No answer 5 (0.7)	

TP, Progress Test; IES, Higher Education Institution.

Machine Translated by Google

The Table below shows the distribution of students' responses to the items that characterize the motivation and use of the TP results by the student himself for his own academic development.

Table

Motivation and use of TP results by the student himself for his academic development

Items	N (%)
Motivated to take the test, n (%)	
I completely disagree I	41 (5.8)
partially disagree I neither	37 (5.2)
agree nor disagree I partially	82 (11.6)
agree I completely agree	179 (25.3)
Did not respond Importance	364 (51.3)
of taking the test	6 (0.8)
for academic development, n (%)	
I completely disagree I	11 (1.6)
partially disagree I neither	9 (1.3)
agree nor disagree I partially	49 (6.9)
agree I completely agree I	129 (18.1)
did not answer It takes into	504 (71.1)
account the	7 (1)
development in the test to assess academic development, n (%)	
I completely disagree I	36 (5.1)
partially disagree I neither	30 (4.2)
agree nor disagree I partially	64 (9)
agree I completely agree I	140 (19.7)
did not answer It takes into	182 (25.7)
account the	257 (36.3)
evolution of performance in the test to guide the studies, n (%)	
Strongly disagree 49 (6.9)	
Partially disagree 31 (4.4)	
Neither agree nor disagree 82 (11.6)	
I partially agree 123 (17.3)	
I completely agree 166 (23.4)	
No answer 258 (36.4)	

Categorical variables are described in number (percentage).

Machine Translated by Google

Results of student self-perception in expected performance in TP, according to the semester completed.

Table

Distribution of responses to the item "Percentage of questions you expect to get right", according to the semester of undergraduate studies taken.

Graduation series	Percentage of questions you expect to get right	N (%)
1st Semester		
	0 to 20%	83/149 (55.8)
	20 to 40%	54/149 (36.2)
	40 to 60%	8/149 (5.4)
	60 to 80%	2/149 (1.3)
	80 to 100%	2/149 (1.3)
2nd Semester		
	0 to 20%	74/111 (66.7)
	20 to 40%	32/111 (28.8)
	40 to 60%	2/111 (1.8)
	80 to 100%	3/111 (2.7)
3rd Semester		
	0 to 20%	14/44 (31.8)
	20 to 40%	26/44 (59.1)
	40 to 60%	3/44 (6.8)
	80 to 100%	1/44 (2.3)
4th Semester		
	0 to 20%	14/104 (13.5)
	20 to 40%	64/104 (61.5)
	40 to 60%	23/104 (22.1)
	60 to 80%	3/104 (2.9)
5th Semester		
	0 to 20%	2/63 (3.2)
	20 to 40%	28/63 (44.4)
	40 to 60%	26/63 (41.3)
	60 to 80%	5/63 (7.9)
	80 to 100%	2/63 (3.2)
6th Semester		
	20 to 40%	4/36 (11.1)
	40 to 60%	22/36 (61.1)
	60 to 80%	8/36 (22.2)
	80 to 100%	2/36 (5.6)
7th Semester		
	20 to 40%	6/36 (16.7)
	40 to 60%	20/36 (55.5)
	60 to 80%	9/36 (25)
	80 to 100%	1/36 (2.8)
8th Semester		
	40 to 60%	17/31 (54.8)

Machine Translated by Google

	60 to 80%	14/31 (45.2)
9th Semester		
	20 to 40%	1/38 (2.6)
	40 to 60%	16/38 (42.1)
	60 to 80%	20/38 (52.7)
	80 to 100%	1/38 (2.6)
10th Semester		
	0 to 20%	1/23 (4.3)
	40 to 60%	11/23 (47.9)
	60 to 80%	10/23 (43.5)
	80 to 100%	1/23 (4.3)
11th Semester		
	20 to 40%	1/31 (3.2)
	40 to 60%	11/31 (35.5)
	60 to 80%	17/31 (54.8)
	80 to 100%	2/31 (6.5)
12th Semester		
	40 to 60%	11/43 (25.6)
	60 to 80%	29/43 (67.4)
	80 to 100%	3/43 (7.0)

Then the semesters were grouped into 2s, to result in “graduation year”.

The distribution of student responses to the item “Percentage of questions expected get it right”, according to the year of graduation, can be seen in the Table below.

Table

Distribution of responses to the item “Percentage of questions you expect to get right”, according to the year of graduation.

	Graduation year					
	1st Year	2nd Year	3rd Year	4th Year	5th Year	6th Year
Percentage of questions you expect to get right	N = 260 N = 148 N = 99 N = 67 N = 61 N = 74					
0 to 20%	157 (60.4) 28 (18.9) 2 (2.0) 0 (0.0) 1 (1.6) 86 (33.1) 90 (60.8) 32 (32.4) 6					0 (0.0)
20 to 40%	(9.0) 1 (1.6) 10 (3.8) 26 (17.6) 48 (48.6) 37 (55.2) 27 (44.3) 22 (29.7)					1 (1.4)
40 to 60%						
60 to 80%	2 (0.8) 3 (2.0) 13 (13.1) 23 (34.3) 30 (49.2) 46 (62.1)					
80 to 100%	5 (1.9) 1 (0.7) 4 (4.0) 5 (6.8)			1 (1.5)	2 (3.3)	

1st Year = 1st semester and 2nd semester; 2nd Year = 3rd semester and 4th semester; 3rd Year = 5th semester and 6th semester; 4th Year = 7th semester and 8th semester; 5th Year = 9th semester and 10th semester; 6th Year = 11th semester and 12th semester.

Machine Translated by Google

3. Correlation analysis

Correlation analysis is appropriate when studying the relationship between two variables that have a numerical or ordinal nature. The correlation coefficient is measured by means of from the value scale +1 to -1; when the value is close to +1, correlation is assumed perfect positive linear (i.e. the higher the value of a variable, the higher it will also be the value of the other variable), and when the value of the coefficient is close to -1, it is assumed the perfect negative linear correlation (i.e., the higher the value of a variable, the lower will be the value of the other); values close to zero indicate the absence of correlation. The strength of correlation between two variables can be interpreted as follows, according to the literature: r |ÿ0.25| = no correlation; |0.26 – 0.50| = weak correlation; |0.51 – 0.75| = moderate correlation and |>0.75| = strong correlation. The result of “r” is that found in the sample. On the other hand, the 95% CI (confidence interval) shows the r values extrapolated to the population, with 95% confidence. Furthermore, the visualization by scatter plots can help in the interpretation of existence or not correlation, by the way the values of the X and Y axes relate to each other.

There was a strong positive correlation between the undergraduate series and the student's perception of the percentage of questions you expect to get right, that is, as the series of questions increases degree, the higher the percentage of questions the student expected to get right. The table below shows the value of the correlation coefficient (rs) and its confidence interval.
Likewise, we can visualize the correlation between the two variables, through the scatter plot.

Table
Correlation between undergraduate series and the item "Percentage of questions you expect to get right".

Item	Graduation series	
	lol	IC (95%)
Percentage of questions expected to be answered	0.752	0.713; 0.786

correctly rs = Spearman's correlation coefficient; CI, confidence interval.

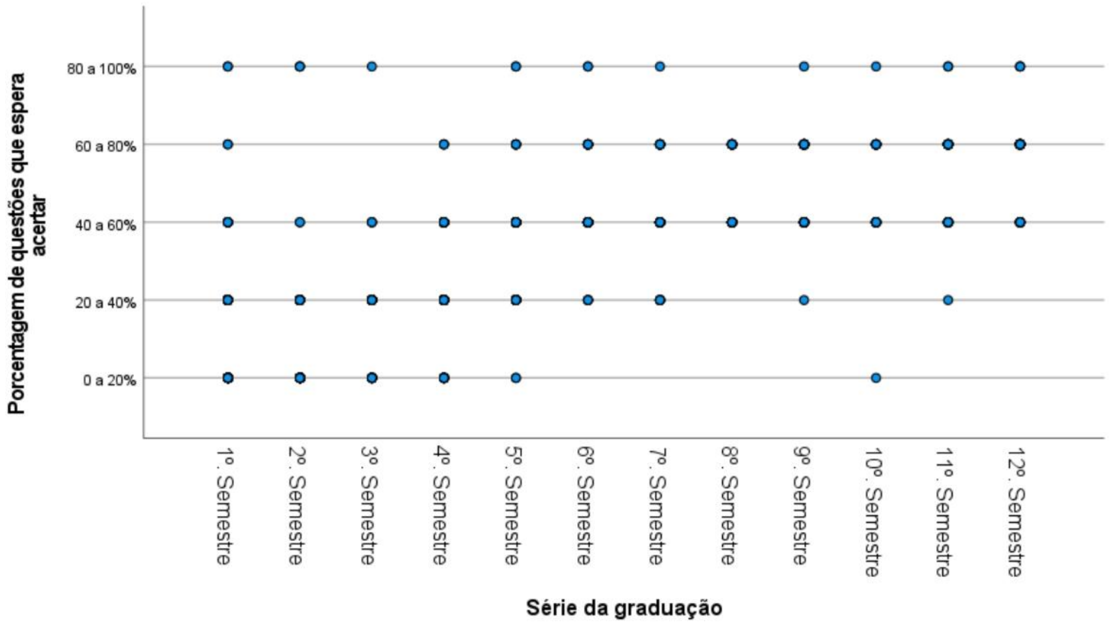

FIGURE

Scatter plot between the item "Percentage of questions you expect to get right" and the series graduation.

Machine Translated by Google

The correlation analysis between the undergraduate series (12 semesters) is presented below. and the responses to each of the 12 items of the instrument, which allowed responses on a scale Likert type.

In the Table below, we can see that there was no correlation between the responses regarding the adequacy of the construction of the TP and possible institutional movements to promote the academic's adherence to carrying it out and the degree series. Therefore, the student perception of these items was independent of the semester attended. This is illustrated in the respective scatter plots.

Table

Correlation between the undergraduate series and the responses on the adequacy of the TP construction and possible institutional movements to promote academic adherence to its completion *.

Item	Graduation series	
	lol	IC (95%)
The questions and alternatives are clear to answer.	-0.033	-0.107; 0.041
Completion time is appropriate for the content	0.153	0.080; 0.225
Received prior information from the Institution about the importance of the test	-0.051	-0.124; 0.023

rs = Spearman correlation coefficient; CI, confidence interval.
*The responses to the instrument's questions were reported using a Likert-type scale, where: 0 = I completely disagree; 1 = I partially disagree; 2 = I neither agree nor disagree; 3 = I partially agree; 4 = I completely agree.

Machine Translated by Google

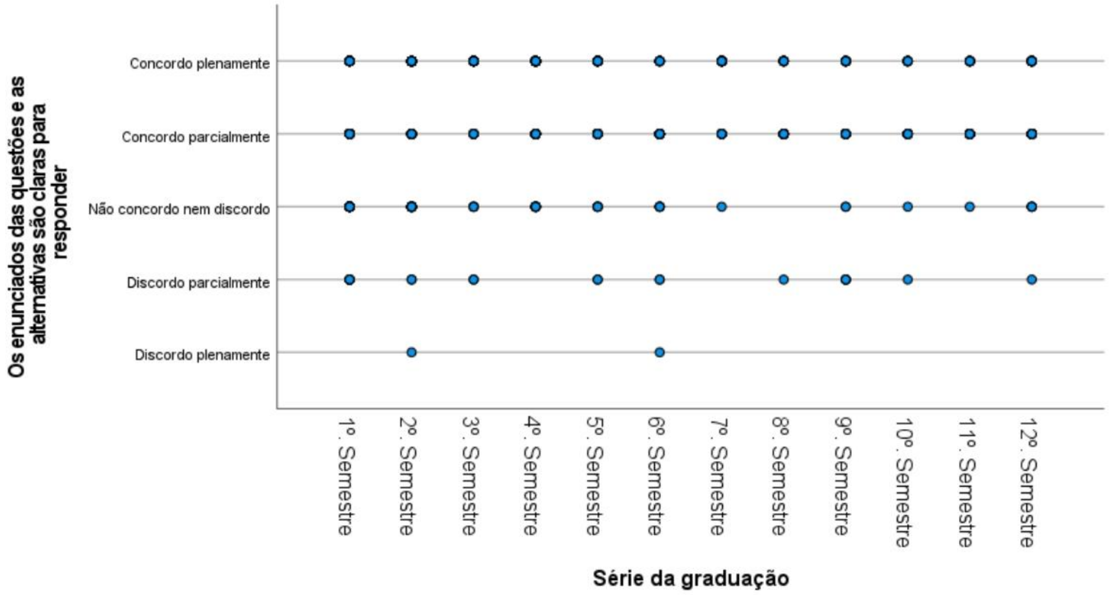

FIGURE

Scatter plot between the item "The questions and alternatives are clear to answer" and undergraduate series.

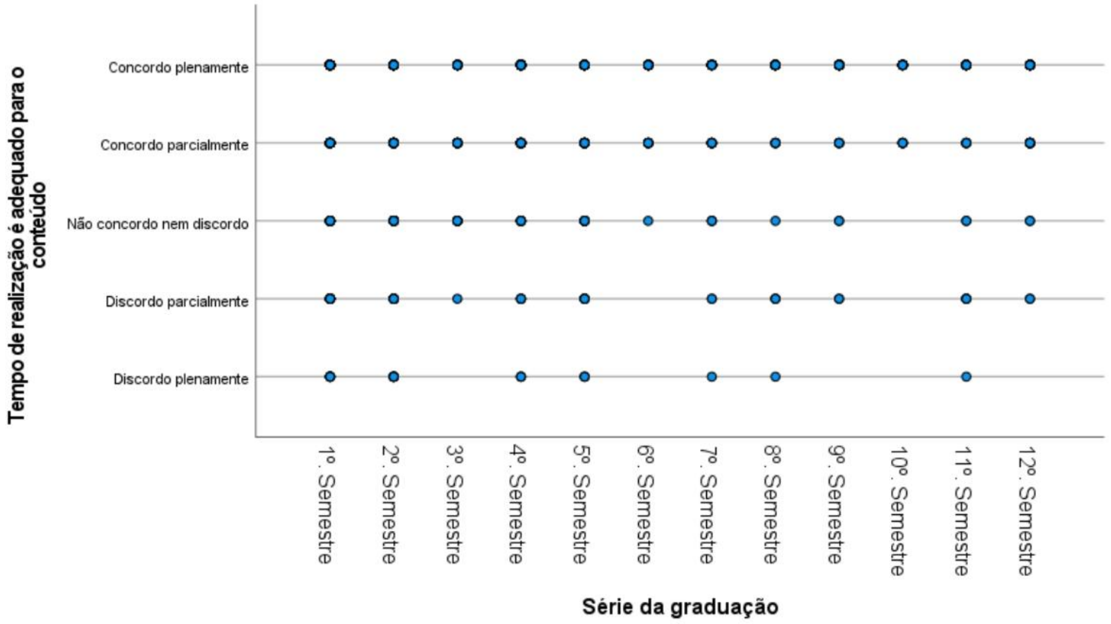

FIGURE

Scatter plot between the item "The completion time is adequate for the content" and the undergraduate series.

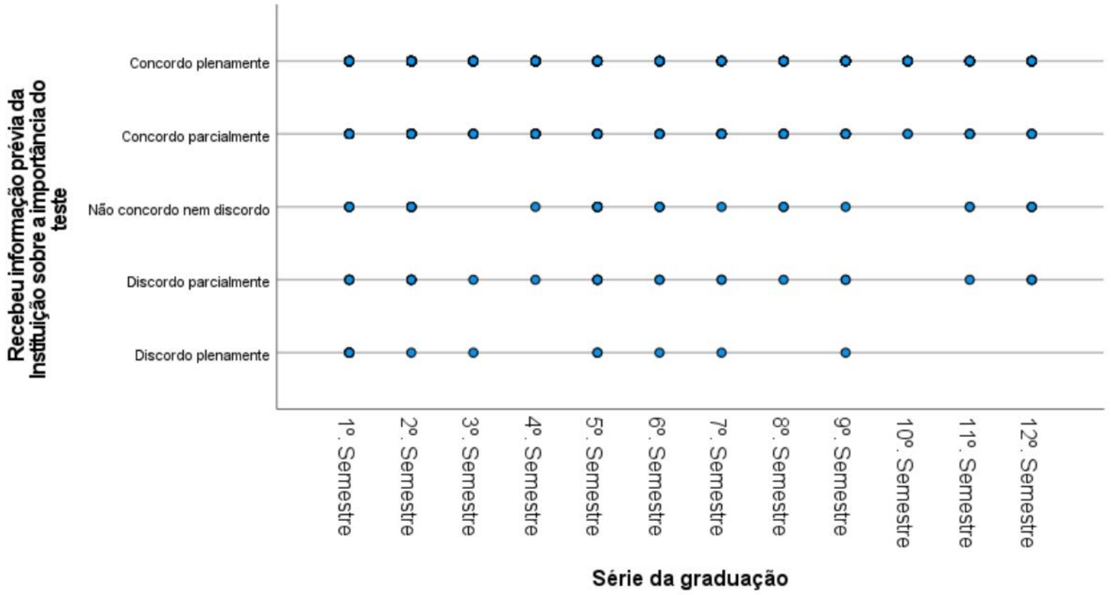

FIGURE

Scatter plot between the item “Received prior information from the Institution about the importance of the test” and undergraduate series.

Machine Translated by Google

In the Table below, we can see the correlation between the answers about whether they intend to access the commented template and TP result and the graduation series. Regarding the item "Do you want to access the commented template", we can see that there was a weak positive correlation (observe the 95% CI), that is, as we progress through the semester, the lower scores have been disappearing and the higher scores remain response (observe the scatter plot). On the other hand, for the item "Do you intend to access the result" is considered to have no correlation.

Table

Correlation between the undergraduate series and the answers about whether you intend to access the commented answer sheet and the TP result*

Item	Graduation series	
	lol	IC (95%)
Do you want to access the commented template?	0.186	0.113; 0.257
Want to access the result?	0.106	0.032; 0.178

rs = Spearman correlation coefficient; CI, confidence interval.

*The responses to the instrument's questions were reported using a Likert-type scale, where: 0 = I completely disagree; 1 = I partially disagree; 2 = I neither agree nor disagree; 3 = I partially agree; 4 = I completely agree.

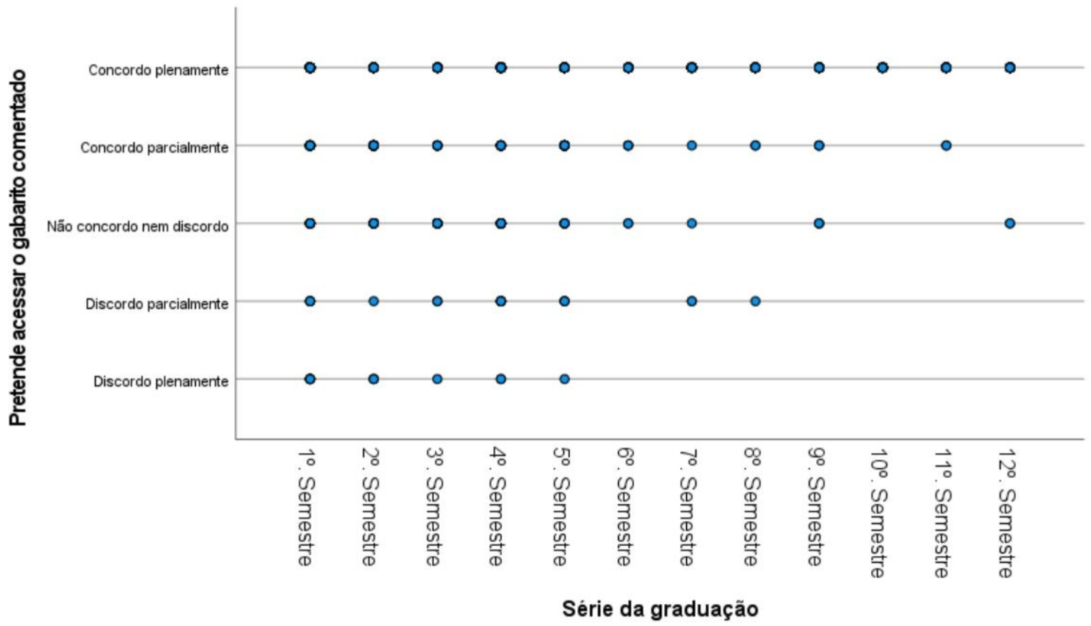

FIGURE

Scatter plot between the item "Do you want to access the commented answer sheet" and the undergraduate series.

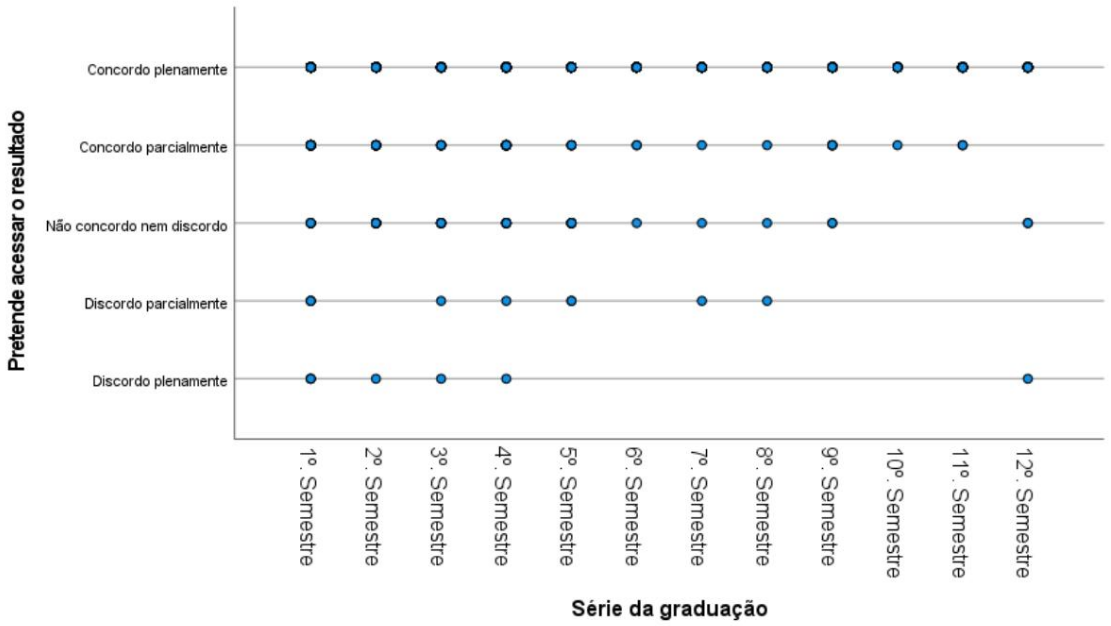

FIGURE

Scatter plot between the item “Do you intend to access the result” and the undergraduate series.

Machine Translated by Google

In the Table below, we can observe the correlation between the responses regarding use of TP results by the HEI and the undergraduate series. The coefficient of correlation for the item “The questions are later discussed in the classroom” demonstrated a weak positive correlation (confirmed by the 95% confidence interval), that is, the further the semester progresses, the higher the score for the responses on the scale Likert.

The student's perception regarding the other questions was independent of the semester attended, as verified by the correlation coefficient. Observe the respective graphs of dispersal.

Table

Correlation between the undergraduate series and the responses regarding the use of TP results by the HEI*.

Item	Graduation series	
	lol	IC (95%)
The questions are then discussed in the classroom.	0.314	0.244; 0.381
Importance of discussing issues in the classroom	0.141	0.068; 0.231
The content covered at your institution is suitable for taking the test	-0.202	-0.273; -0.129

rs = Spearman correlation coefficient; CI, confidence interval.

*The responses to the instrument's questions were reported using a Likert-type scale, where: 0 = I completely disagree; 1 = I partially disagree; 2 = I neither agree nor disagree; 3 = I partially agree; 4 = I completely agree.

Machine Translated by Google

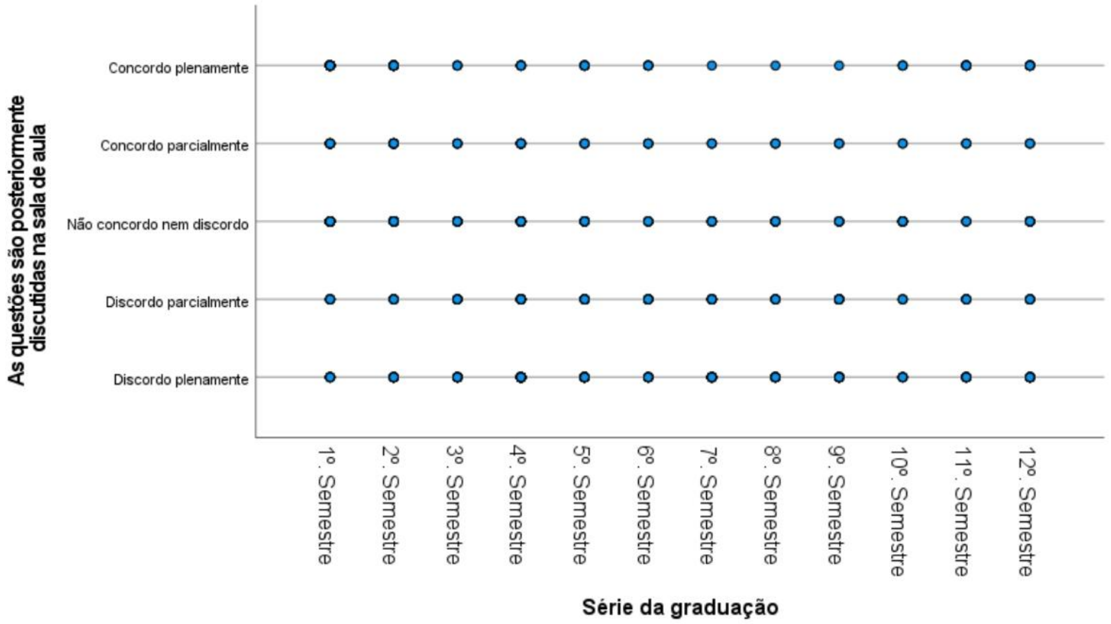

FIGURE

Scatterplot between the item "The questions are later discussed in the classroom" and undergraduate series.

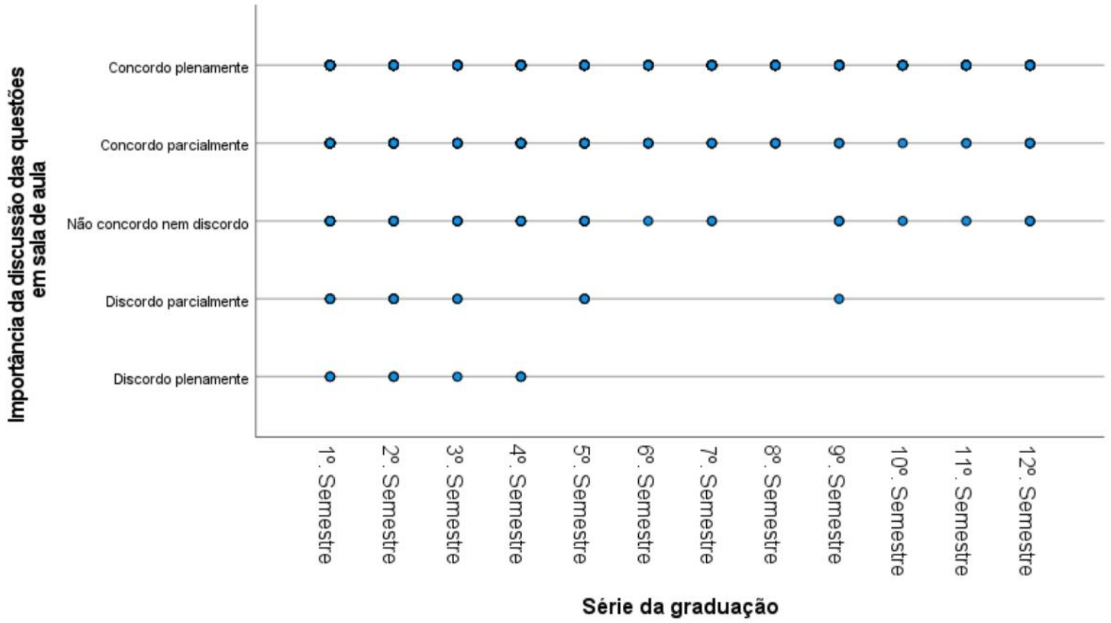

FIGURE

Scatter plot between the item "Importance of discussing issues in the classroom" and undergraduate series.

Machine Translated by Google

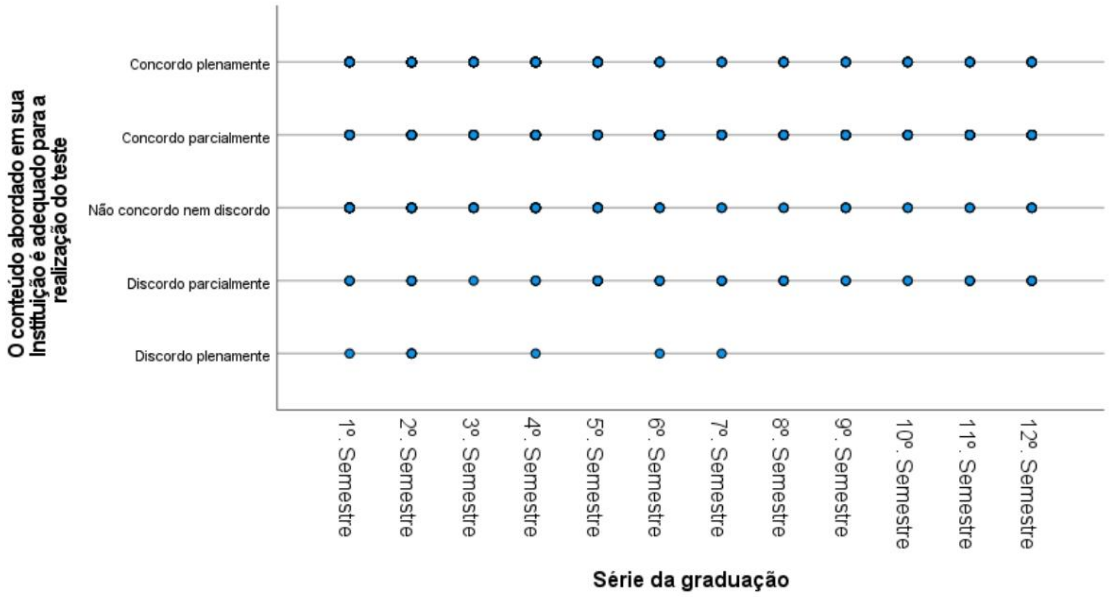

FIGURE

Scatter plot between the item “The content covered in your Institution is suitable for taking the test” and undergraduate series.

Machine Translated by Google

In the Table below, we can see that there was no correlation between the responses regarding motivation and use of TP results by the student himself for his own development academic and undergraduate series. Therefore, the student's perception of these issues was independent of the semester attended. This is illustrated in the respective graphs of dispersal.

Table

Correlation between the undergraduate series and the responses regarding the motivation and use of the TP results by the student himself for his academic development*.

Item	Graduation series	
	lol	IC (95%)
Motivated to take the test	-0.082	-0.155; -0.008
Importance of taking the test for academic development	-0.129	-0.201; -0.055
Takes into account development on the test to assess academic development	-0.103	-0.194; -0.011
Takes into account the evolution of test performance to guide studies	0.127	0.035; 0.217

rs = Spearman correlation coefficient; CI, confidence interval.

*The responses to the instrument's questions were reported using a Likert-type scale, where: 0 = I completely disagree; 1 = I partially disagree; 2 = I neither agree nor disagree; 3 = I partially agree; 4 = I completely agree.

Machine Translated by Google

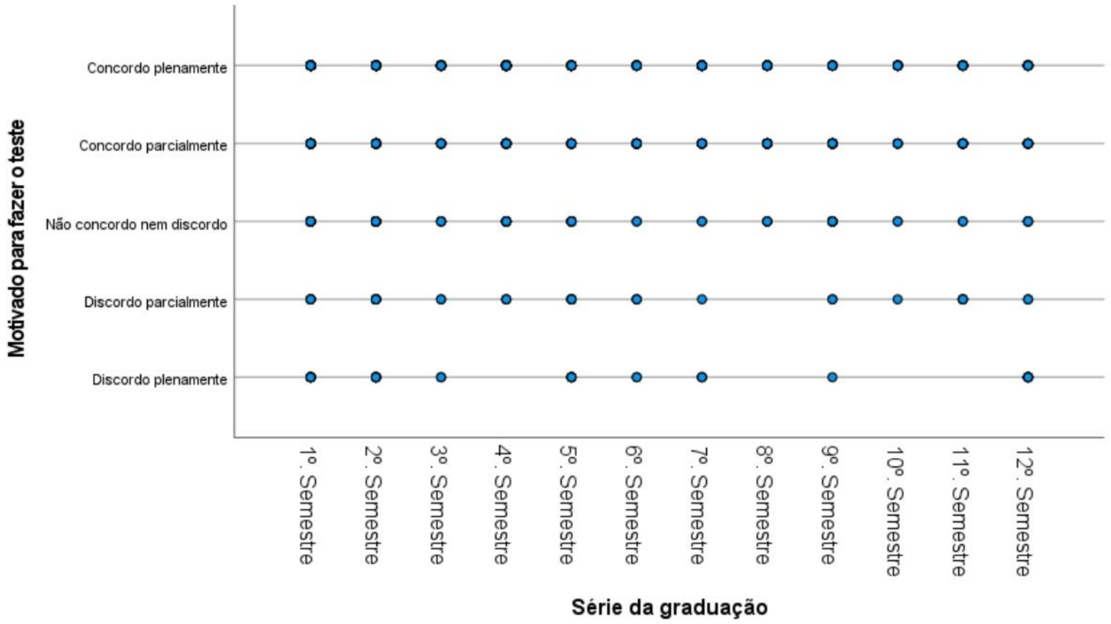

FIGURE

Scatter plot between the item “Motivated to take the test” and undergraduate series.

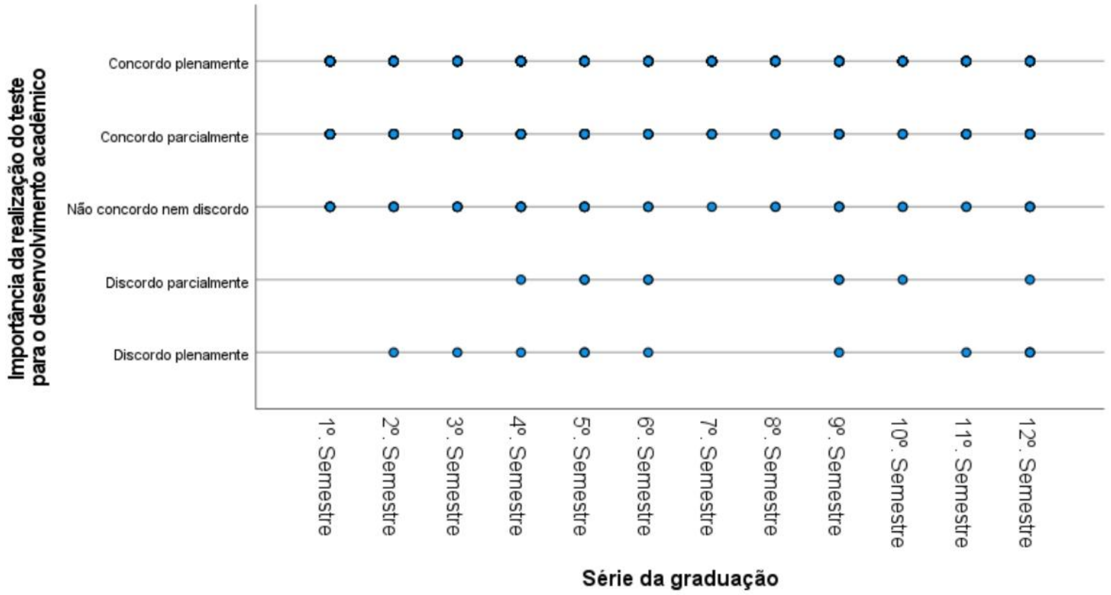

FIGURE

Scatter plot between the item “Importance of taking the test for academic development” and undergraduate series.

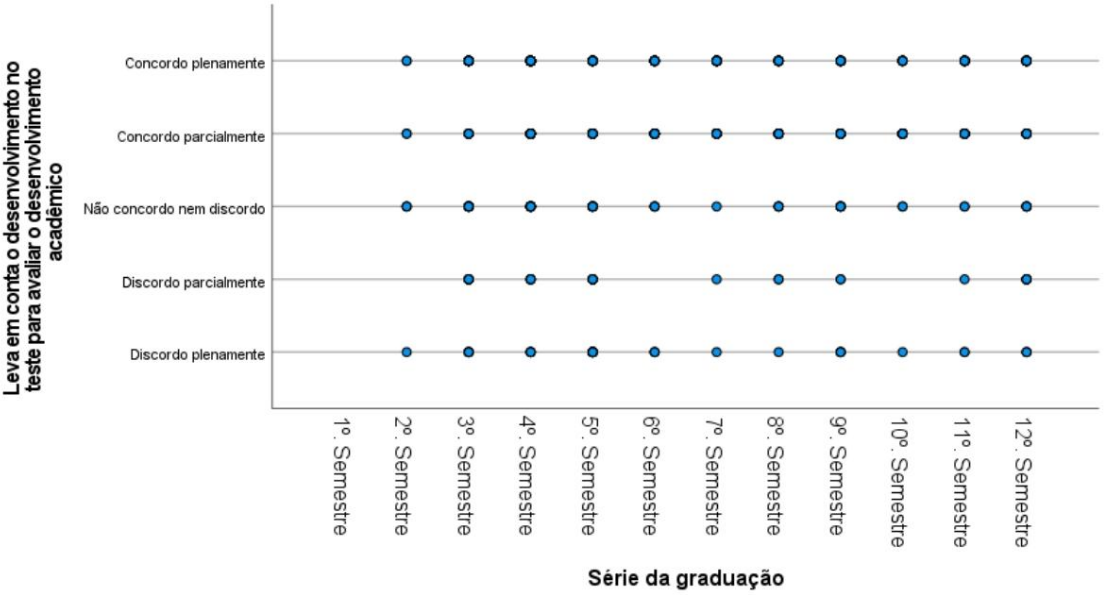

FIGURE

Scatter plot between the item “Takes into account development in the test to assess academic development” and undergraduate series.

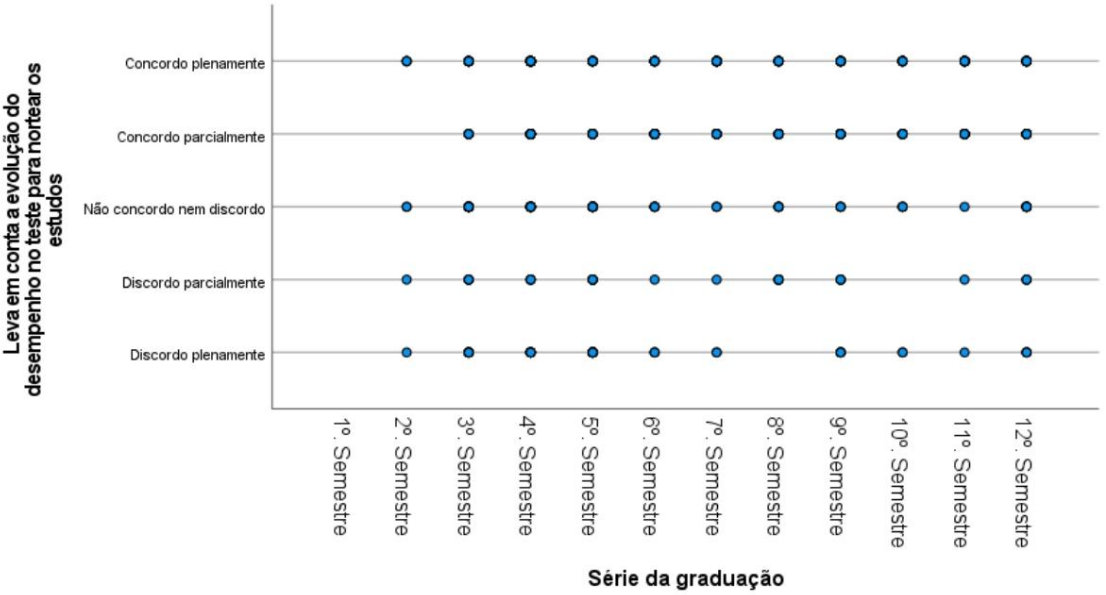

FIGURE

Scatter plot between the item “Take into account the evolution of test performance to guide studies” and undergraduate series.

4. References

1. CONOVER, WJ **Practical nonparametric statistics.** New York: John Wiley & Sons, 1999.
2. SIEGEL S.; CASTELLAN Jr NJ. **Nonparametric Statistics for Behavioral Sciences.** Bookman, 2nd edition, São Paulo, 2006.
3. BONETT, D. G; WRIGHT, TA **Sample size requirements for estimating Pearson, Kendall and Spearman correlations.** Psychometrika, 65, 23–28, 2000.
4. BISHARA, A. J; HITTNER JB **Confidence intervals for correlations when data are not normal.** Behav Res 49, 294–309, 2017.
