# Supplementary material for: Continuous assessment in medical education: Exploring students’ views on the progress test
Source: PLoS One. 2024 Dec 19;19(12):e0314848. doi: 10.1371/journal.pone.0314848 (PMC11658631; doi:10.1371/journal.pone.0314848)
Supplement: S4 File — (PDF) [file pone.0314848.s005.pdf]

Machine Translated by Google

Report of study results:

# The progress test of whoever performs it: the student

September 8, 2021

Machine Translated by Google

# SUMMARY

I. Methodology of statistical analysis .............................................................. 3

1. Sample characterization ........................................................ 3
2. Database .......................................................................................... 3
3. Statistical analysis of data .................................................................... 3

II. Results ............................................................................................... 4

1. ANALYSIS 1 (FAMERP) .............................................................. 4
2. ANALYSIS 2 (comparison between the two HEIs) ............................ 26

III. References ............................................................................................ 33

Machine Translated by Google

# I - Methodology of statistical analysis

## 1. Sample characterization

The study sample consisted of 220 FAMERP students and 709 students from UNISA.

## 2. Database

The data was received registered in Excel. Later, it was imported into IBM-SPSS *Statistics* version 28 software (IBM Corporation, NY, USA) for exploratory data analysis and comparative analysis between groups.

### 3. Statistical analysis of data

Exploratory data analysis included descriptive statistics, mean, median, standard deviation, minimum value and maximum value for numerical variables and number and proportion for categorical variables. Spearman correlation analysis was carried out to verify the correlation between discrete and ordinal variables (semester of graduation and questionnaire item); the result was demonstrated by the coefficient of Spearman correlation and its respective 95% confidence interval (BONETT & WRIGHT, T. A, 2000; CONOVER, 1999; SIEGEL & CASTELLAN, 2006; BISHARA & HITTNER, 2017). The comparison between students from the two HEIs was carried out using the test Pearson's chi-square or Fisher's exact test (CONOVER, 1999; SIEGEL & CASTELLAN, 2006; FIELD, 2009) When statistical significance was found, a multiple comparisons ( pairwise *method)* by z-test for difference in proportions (BUSSAB & MORETTIN, 2017), with p-values adjusted by the correction of Bonferroni, to verify where the effect of the differences was; the results were expressed in the tables as numbers and percentages, with letter notations indicating the similarities or differences between categories (IBM SPSS Statistics Algorithms, 2020; MACDONALD & GARDNER, 2000). Statistical analysis was performed using the IBM-SPSS *Statistics* version 28 (IBM Corporation, NY, USA) and R (R CORE) software TEAM, 2015).

Machine Translated by Google

## II – Results

### ANALYSIS 1: FAMERP

#### 1. Descriptive analysis of the sample

The table below shows the age range and semester of graduation among the 220 students who participated in the survey.

Table

Age range and semester of graduation among the 220 participants included in the study.

| Variable | N = 220 |
|---|---|
| Age, n (%) | |
| 17 to 20 years old | 32 (14.5) |
| 21 to 25 years old | 131 (59.5) |
| 26 to 30 years old | 38 (17.3) |
| 31 to 35 years old | 5 (2.3) |
| Over 40 years old No | 1 (0.5) |
| answer | 13 (5.9) |
| Undergraduate semester, n (%) | |
| 2nd. Semester of the 1st year (2nd Semester) | 46 (20.9) |
| 2nd. Semester of the 2nd year (4th Semester) | 32 (14.5) |
| 2nd. Semester of the 3rd year (6th Semester) | 42 (19.1) |
| 2nd. Semester of the 5th year (10th Semester) | 37 (16.8) |
| 2nd. Semester of the 6th year (12th Semester) | 50 (22.8) |
| Did not respond | 13 (5.9) |

Categorical variables are described in number (percentage)

Comment: Students from the 2nd semester of the 4th year (8th grade) did not participate in the research. Semester) of the course.

Machine Translated by Google

**2. Descriptive analysis of the responses to the questions in the instrument applied**

Next, the Table below shows the distribution of students' responses to the items applied, in relation to the student's self-perception of the expected performance in the TP.

Table

Student self-perception of expected performance in the Progress Test.

| Items | N (%) |
|---|---|
| Percentage of questions you expect to get right, n (%) | |
| 0 to 20% | 32 (14.5) |
| 20 to 40% | 52 (23.6) |
| 40 to 60% | 44 (20) |
| 60 to 80% | 55 (25) |
| 80 to 100% | 23 (10.5) |
| Did not respond | 14 (6.4) |
| Area that believes it has better performance, n (%) | |
| Basic | 71 (32.4) |
| Collective health | 44 (20) |
| Clinic | 41 (18.6) |
| Surgery | 30 (13.6) |
| GO | 11 (5) |
| Pediatrician | 10 (4.5) |
| No answer GO, | 13 (5.9) |

obstetrics gynecology.

Machine Translated by Google

The Table below shows the distribution of students' responses to the items, in relation to the adequacy of the construction of the TP and possible institutional movements to promote the academic's adherence to carrying it out.

Table

Adequacy of the construction of the TP and possible institutional movements to promote academic participation in its implementation.

| Items | N (%) |
|---|---|
| The questions and alternatives are clear to answer, n (%) | |
| I partially disagree | 9 (4.1) |
| I neither agree nor disagree | 17 (7.7) |
| I partially agree | 90 (40.9) |
| I completely agree | 91 (41.4) |
| Did not respond | 13 (5.9) |
| Completion time is adequate for the content, n (%) | |
| I completely disagree | 11 (5) |
| I partially disagree | 14 (6.4) |
| I neither agree nor disagree | 21 (9.5) |
| I partially agree | 44 (20) |
| I completely agree | 117 (53.2) |
| Did not respond | 13 (5.9) |
| Received prior information from the Institution about the importance of the test, n (%) | |
| I completely disagree | 1 (0.5) |
| I partially disagree | 9 (4.1) |
| I neither agree nor disagree | 11 (5) |
| I partially agree | 30 (13.6) |
| I completely agree | 156 (70.9) |
| Did not respond | 13 (5.9) |

Categorical variables are described in number (percentage)

Machine Translated by Google

The Table below shows the distribution of students' responses to the items about whether they intend to access the commented template and the TP result.

Table

Items about whether you want to access the commented answer sheet and the TP result.

| Variables | N (%) |
|---|---|
| Do you want to access the commented template, n (%) | |
| I completely disagree | 3 (1.4) |
| I partially disagree | 8 (3.6) |
| I neither agree nor disagree | 14 (6.4) |
| I partially agree | 31 (14.1) |
| I completely agree | 151 (68.6) |
| Did not respond | 13 (5.9) |
| Do you want to access the result, n (%) | |
| I completely disagree | 2 (0.9) |
| I partially disagree | 2 (0.9) |
| I neither agree nor disagree | 10 (4.5) |
| I partially agree | 21 (9.5) |
| I completely agree | 172 (78.3) |
| Did not respond | 13 (5.9) |

Categorical variables are described in number (percentage)

Machine Translated by Google

The Table below shows the distribution of students' responses regarding achievement of the TP results by the IES.

Table

Use of TP results by the HEI.

| Items | N (%) |
|---|---|
| The issues are then discussed in the classroom, n (%) | |
| I completely disagree | 128 (58.2) |
| I partially disagree | 22 (10) |
| I neither agree nor disagree | 47 (21.4) |
| I partially agree | 2 (0.9) |
| I completely agree | 6 (2.7) |
| Did not respond | 15 (6.8) |
| Importance of discussing issues in class, n (%) | |
| I partially disagree | 1 (0.5) |
| I neither agree nor disagree | 14 (6.4) |
| I partially agree | 50 (22.6) |
| I completely agree | 141 (64.1) |
| Did not respond | 14 (6.4) |
| The content covered in your Institution is suitable for taking the test, n (%) | |
| I partially disagree | 13 (5.9) |
| I neither agree nor disagree | 45 (20.5) |
| Partially agree 94 (42.7) | |
| I completely agree 54 (24.5) | |
| No answer 14 (6.4) | |

TP, Progress Test; IES, Higher Education Institution.

Machine Translated by Google

The Table below shows the distribution of students' responses to the items that characterize the motivation and use of the TP results by the student himself for his own academic development.

Table

Motivation and use of TP results by the student himself for his academic development

| Items | N (%) |
|---|---|
| Motivated to take the test, n (%) | |
| I completely disagree I | 16 (7.3) |
| partially disagree I neither | 25 (11.4) |
| agree nor disagree I partially | 40 (18.2) |
| agree I completely agree | 58 (26.3) |
| Did not respond Importance | 67 (30.4) |
| of taking the test | 14 (6.4) |
| for academic development, n (%) | |
| I completely disagree I | 6 (2.7) |
| partially disagree I neither | 10 (4.5) |
| agree nor disagree I partially | 21 (9.5) |
| agree I completely agree I | 64 (29.1) |
| did not answer It takes into | 105 (47.8) |
| account the | 14 (6.4) |
| development in the test to assess academic development, n (%) | |
| I completely disagree I | 19 (8.6) |
| partially disagree I neither | 18 (8.2) |
| agree nor disagree I partially | 25 (11.4) |
| agree I completely agree I | 51 (23.2) |
| did not answer It takes into | 52 (23.6) |
| account the | 55 (25) |
| evolution of performance in the test to guide the studies, n (%) | |
| Strongly disagree 24 (10.9) | |
| Partially disagree 25 (11.4) | |
| I neither agree nor disagree 31 (14.1) | |
| Partially agree 48 (21.8) | |
| I completely agree 37 (16.8) | |
| No answer 55 (25) | |

Categorical variables are described in number (percentage).

Machine Translated by Google

Results of student self-perception in expected performance in TP, according to the semester completed.

Table

Distribution of responses to the item "Percentage of questions you expect to get right", according to the semester of undergraduate studies taken.

| Graduation Series | Percentage of questions waiting for get it right | N (%) |
|---|---|---|
| 2nd Semester of the 1st year (2nd Semester) | | |
| | 0 to 20% | 31/46 (67.4) |
| | 20 to 40% | 14/46 (30.4) |
| | 80 to 100% | 1/46 (2.2) |
| 2nd Semester of 2nd Year (4th Semester) | | |
| | 0 to 20% | 1/32 (3.1) |
| | 20 to 40% | 17/32 (53.1) |
| | 40 to 60% | 12/32 (37.5) |
| | 60 to 80% | 2/32 (6.3) |
| 2nd Semester of the 3rd year (6th Semester) | | |
| | 20 to 40% | 17/42 (40.5) |
| | 40 to 60% | 21/42 (50) |
| | 60 to 80% | 4/42 (9.5) |
| 2nd Semester of the 5th year (10th Semester) | | |
| | 20 to 40% | 2/36 (5.6) |
| | 40 to 60% | 5/36 (13.9) |
| | 60 to 80% | 25/36 (69.4) |
| | 80 to 100% | 4/36 (11.1) |
| 2nd Semester of 6th year (12th Semester) | | |
| | 20 to 40% | 2/50 (4) |
| | 40 to 60% | 6/50 (12) |
| | 60 to 80% | 24/50 (48) |
| | 80 to 100% | 18/50 (36) |

Categorical variables are described in number (percentage).

Machine Translated by Google

### 3. Correlation analysis

Correlation analysis is appropriate when studying the relationship between two variables that have a numerical or ordinal nature. The correlation coefficient is measured by means of from the value scale +1 to -1; when the value is close to +1, correlation is assumed perfect positive linear (i.e. the higher the value of a variable, the higher it will also be the value of the other variable), and when the value of the coefficient is close to -1, it is assumed the perfect negative linear correlation (i.e., the higher the value of a variable, the lower will be the value of the other); values close to zero indicate the absence of correlation. The strength of correlation between two variables can be interpreted as follows, according to the literature: r |ÿ0.25| = no correlation; |0.26 – 0.50| = weak correlation; |0.51 – 0.75| = moderate correlation and |>0.75| = strong correlation. The result of “r” is that found in the sample. On the other hand, the 95% CI (confidence interval) shows the r values extrapolated to the population, with 95% confidence. Furthermore, the visualization by scatter plots can help in the interpretation of existence or not correlation, by the way the values of the X and Y axes relate to each other.

Machine Translated by Google

There was a strong positive correlation between the semester of graduation and the student's perception on the percentage of questions you expect to get right, that is, as the semester of graduation, the higher the percentage of questions that the student expected to answer correctly. The table below shows the value of the correlation coefficient (rs) and its range. confidence. Similarly, we can visualize the correlation between the two variables, through the scatter plot.

Table

Correlation between undergraduate semester and the item “Percentage of questions expected to be answered correctly”.

| Item | Undergraduate Semester | |
|---|---|---|
| | lol | IC (95%) |
| Percentage of questions expected to be answered | 0.817 | 0.756; 0.863 |

correctly rs = Spearman's correlation coefficient; CI, confidence interval.

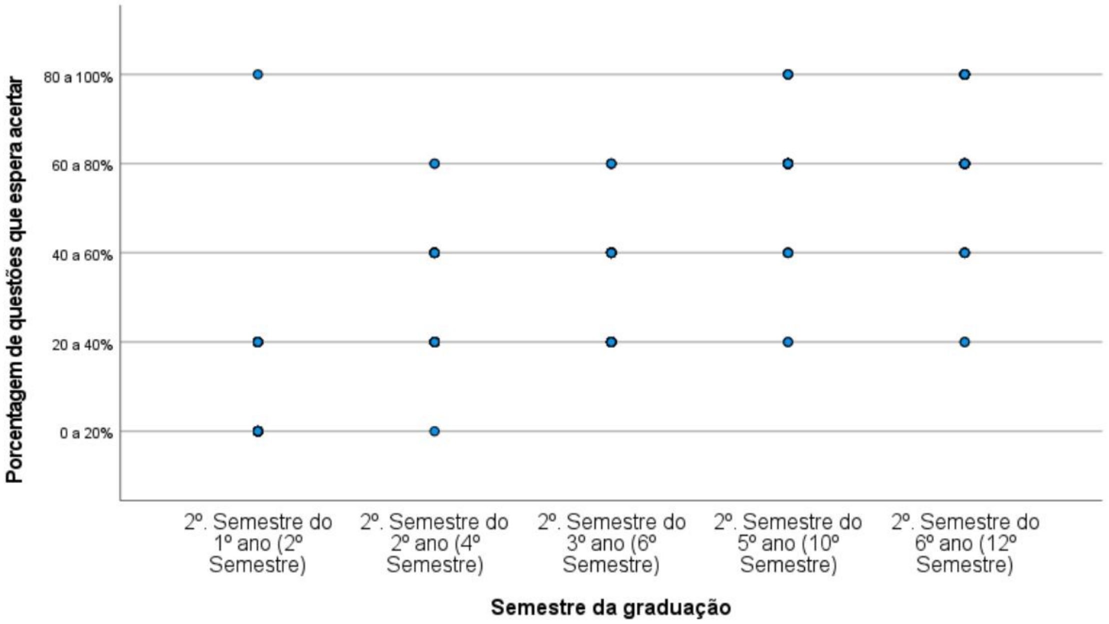


**FIGURE**

Scatter plot between the item “Percentage of questions you expect to get right” and semester of graduation.

Machine Translated by Google

The following is a correlation analysis between the undergraduate semester (2nd semester) of each year of the course) and the responses to each of the 12 items of the instrument, which allowed response on a Likert scale.

In the Table below, we can observe the correlation between the responses regarding adequacy the construction of the TP and possible institutional movements to promote the adhesion of the academic period in carrying it out and the semester of graduation. Regarding the item “Time of realization is adequate for the content”, we can observe that there was a weak positive correlation (observe the 95% CI), that is, as we progress through the semester, the lower scores have been disappearing and the higher scores remain response (observe the scatter plot). On the other hand, for the items “The statements of the questions and the alternatives are clear to answer” and “Received prior information of the Institution on the importance of the test” it is considered that there was no correlation.

Table

Correlation between the undergraduate semester and the responses regarding the adequacy of the construction of the TP and possible institutional movements to promote academic adherence to its completion *.

| Item | Undergraduate Semester | |
|---|---|---|
| | lol | IC (95%) |
| The questions and alternatives are clear to answer. | 0.087 | -0.050; 0.221 |
| Completion time is appropriate for the content | 0.441 | 0.318; 0.549 |
| Received prior information from the Institution about the importance of the test | 0.203 | 0.067; 0.332 |

rs = Spearman correlation coefficient; CI, confidence interval.
*The responses to the instrument's questions were reported using a Likert-type scale, where: 0 = I completely disagree; 1 = I partially disagree; 2 = I neither agree nor disagree; 3 = I partially agree; 4 = I completely agree.

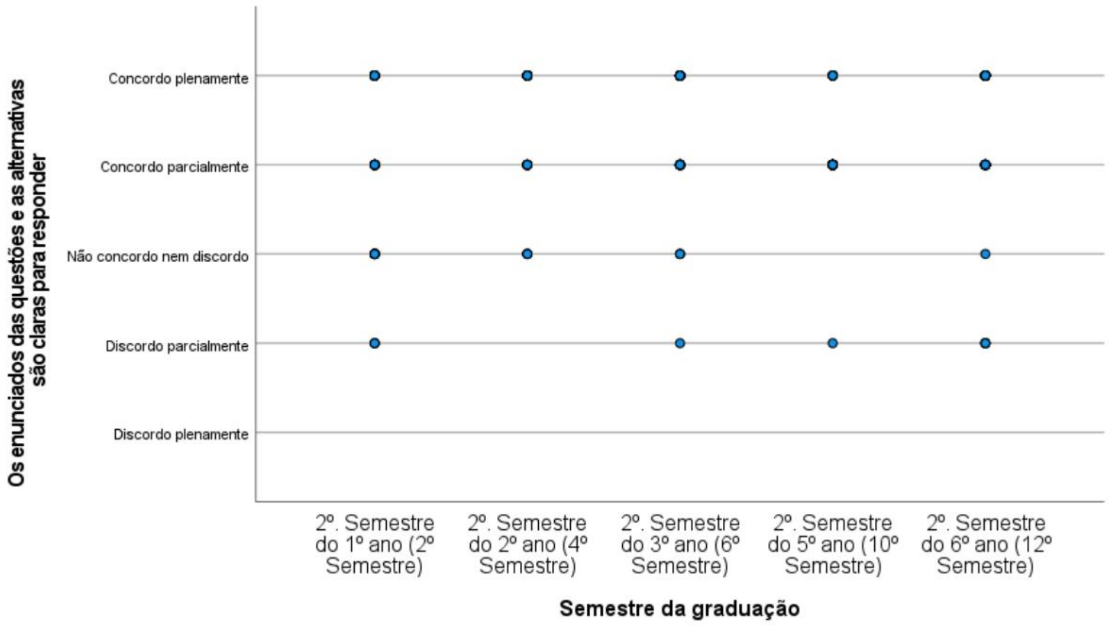


**FIGURE**

Scatter plot between the item "The questions and alternatives are clear to answer" and undergraduate semester.

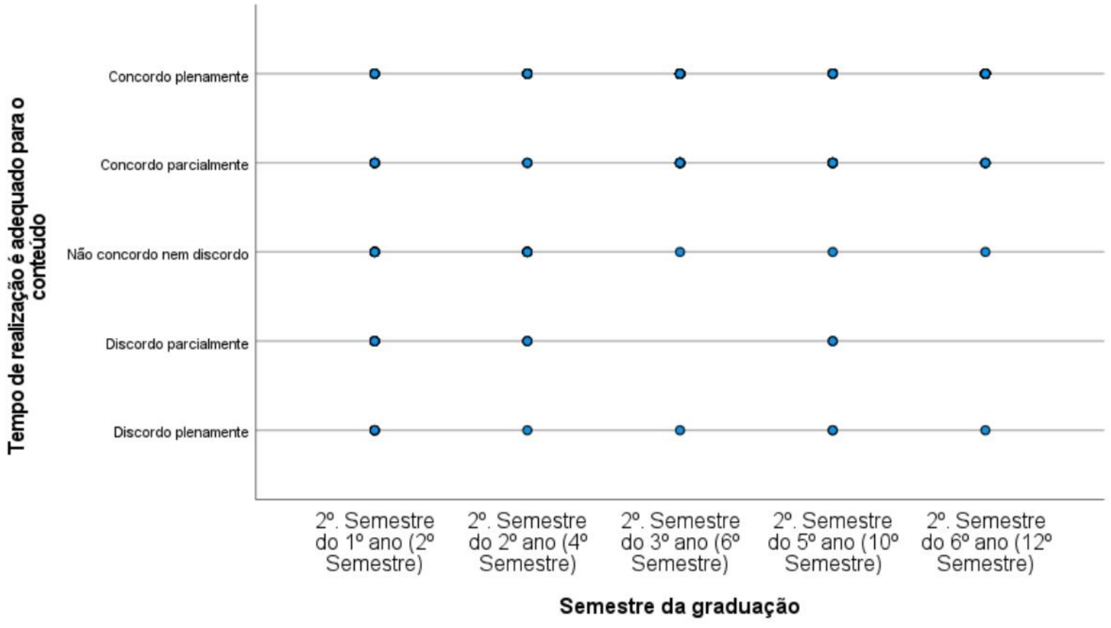


**FIGURE**

Scatter plot between the item "The completion time is adequate for the content" and undergraduate semester.

Machine Translated by Google

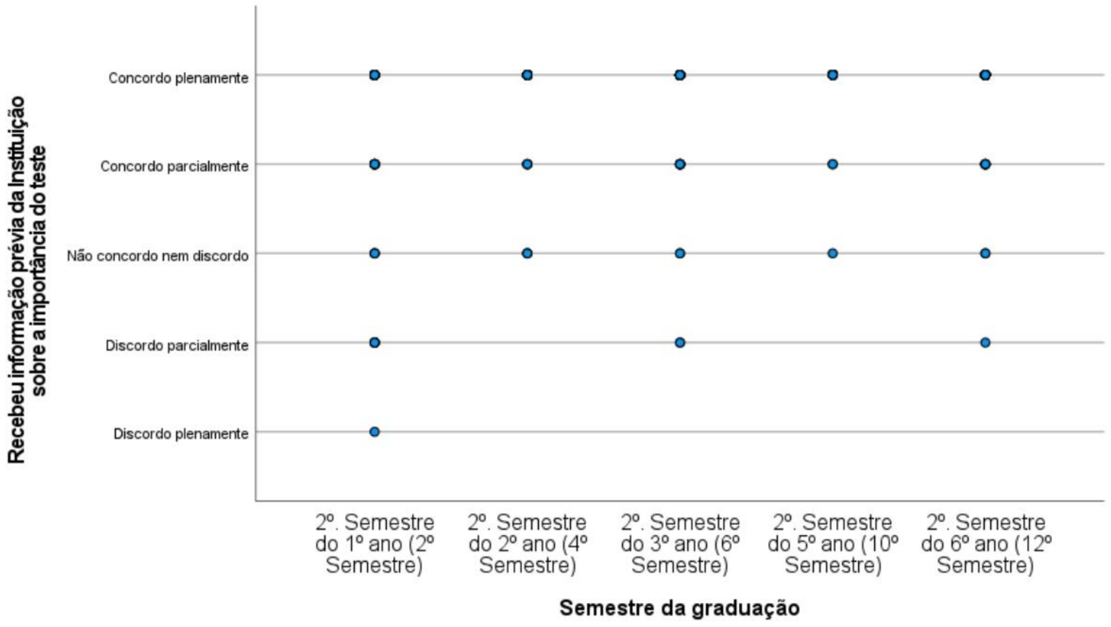


**FIGURE**

Scatter plot between the item “Received prior information from the Institution about the importance of the test” and semester of graduation.

Machine Translated by Google

In the Table below, we can see the correlation between the answers about whether they intend to access the commented template and TP result and the semester of graduation. As for the item “Do you want to access the commented template”, we can see that there was a weak positive correlation (observe the 95% CI), that is, as we progress through the semester, the lower scores have been disappearing and the higher scores remain response (observe the scatter plot). On the other hand, for the item “Do you intend to access the result” is considered to have no correlation.

Table

Correlation between the semester of the undergraduate course and the answers about whether they intend to access the commented answer sheet and the TP result*

| Item | Undergraduate Semester | |
|---|---|---|
| | lol | IC (95%) |
| Do you want to access the commented template? | 0.296 | 0.164; 0.418 |
| Want to access the result? | 0.095 | -0.042; 0.229 |

rs = Spearman correlation coefficient; CI, confidence interval.

*The responses to the instrument's questions were reported using a Likert-type scale, where: 0 = I completely disagree; 1 = I partially disagree; 2 = I neither agree nor disagree; 3 = I partially agree; 4 = I completely agree.

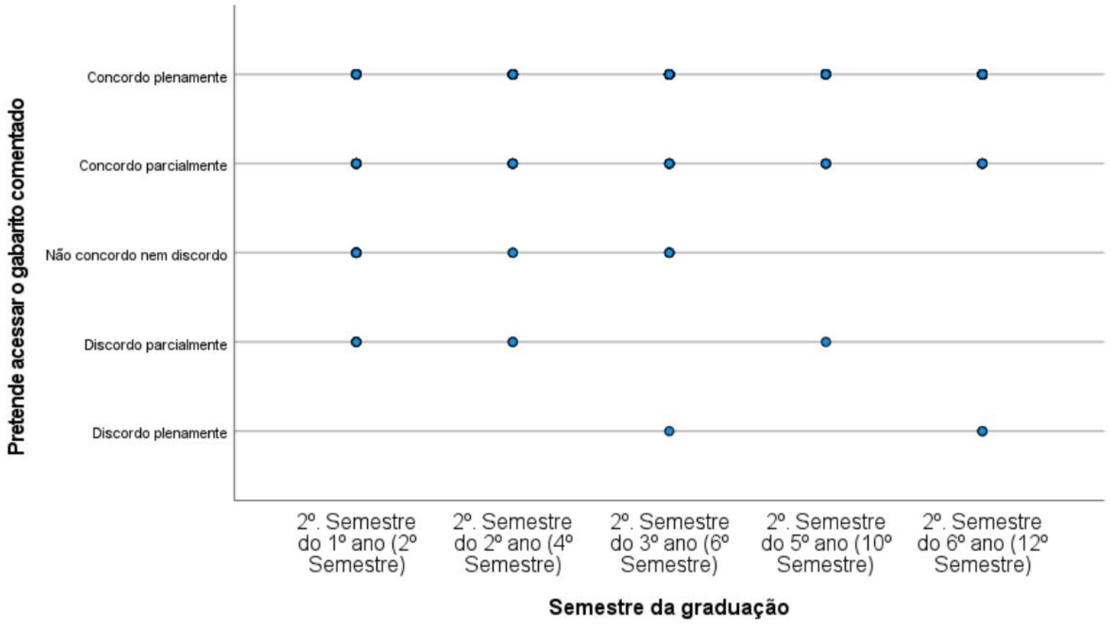


**FIGURE**

Scatter plot between the item “Do you intend to access the commented answer sheet” and undergraduate semester.

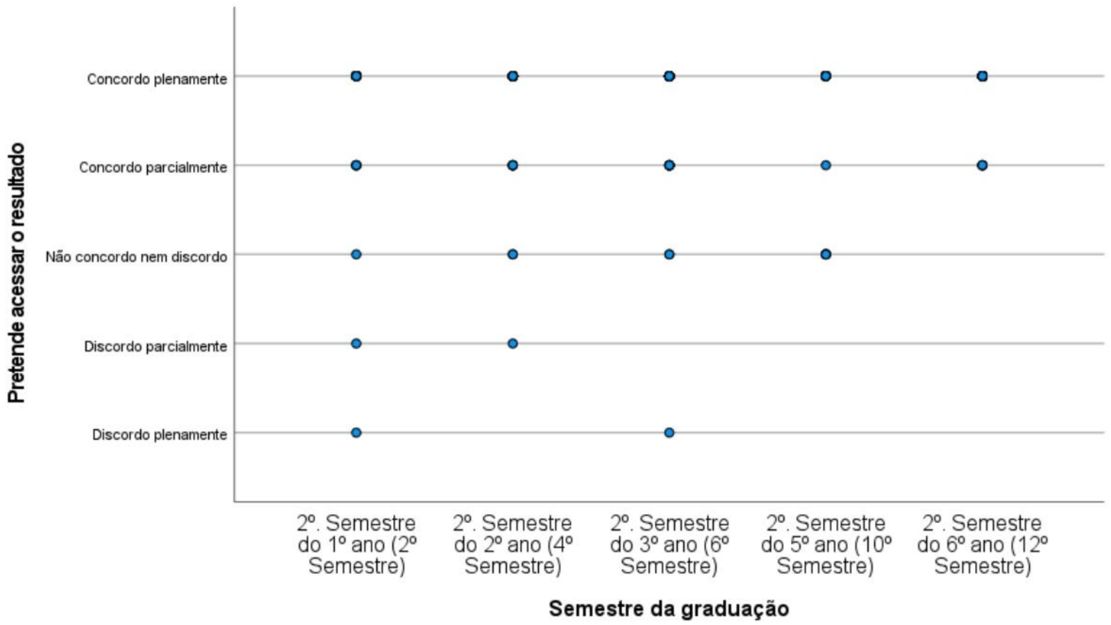


**FIGURE**

Scatter plot between the item “Intend to access the result” and semester of graduation.

In the Table below, we can observe the correlation between the responses regarding use of TP results by the HEI and the semester of graduation. The coefficient correlation for the item “The questions are later discussed in the classroom” demonstrated a weak negative correlation (confirmed by the 95% confidence interval), that is, the further the semester progresses, the lower the score for the scaled responses Likert.

The student's perception regarding the other questions was independent of the semester attended, as verified by the correlation coefficient. Observe the respective graphs of dispersal.

Table

Correlation between the undergraduate semester and the responses regarding the use of TP results by the HEI*.

| Item | Undergraduate Semester | |
|---|---|---|
| | lol | IC (95%) |
| The questions are then discussed in the classroom. | -0.411 | -0.523; -0.285 |
| Importance of discussing issues in the classroom | 0.154 | 0.012; 0.290 |
| The content covered at your institution is suitable for taking the test | 0.157 | 0.015; 0.293 |

rs = Spearman correlation coefficient; CI, confidence interval.

*The responses to the instrument's questions were reported using a Likert-type scale, where: 0 = I completely disagree; 1 = I partially disagree; 2 = I neither agree nor disagree; 3 = I partially agree; 4 = I completely agree.

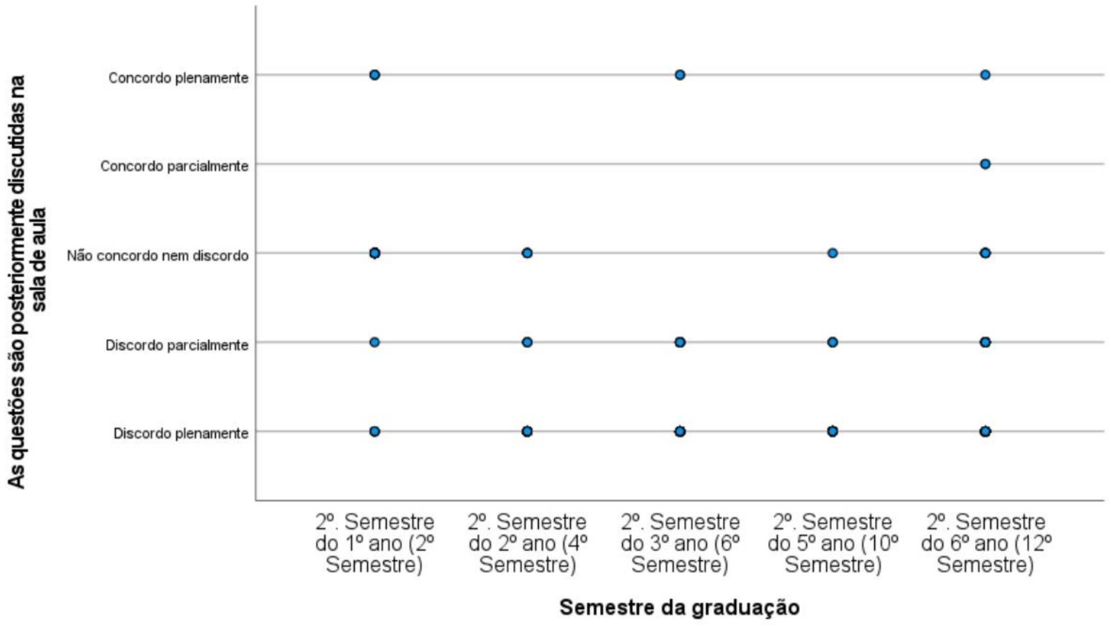


**FIGURE**

Scatter plot between the item "The questions are later discussed in the classroom" and undergraduate semester.

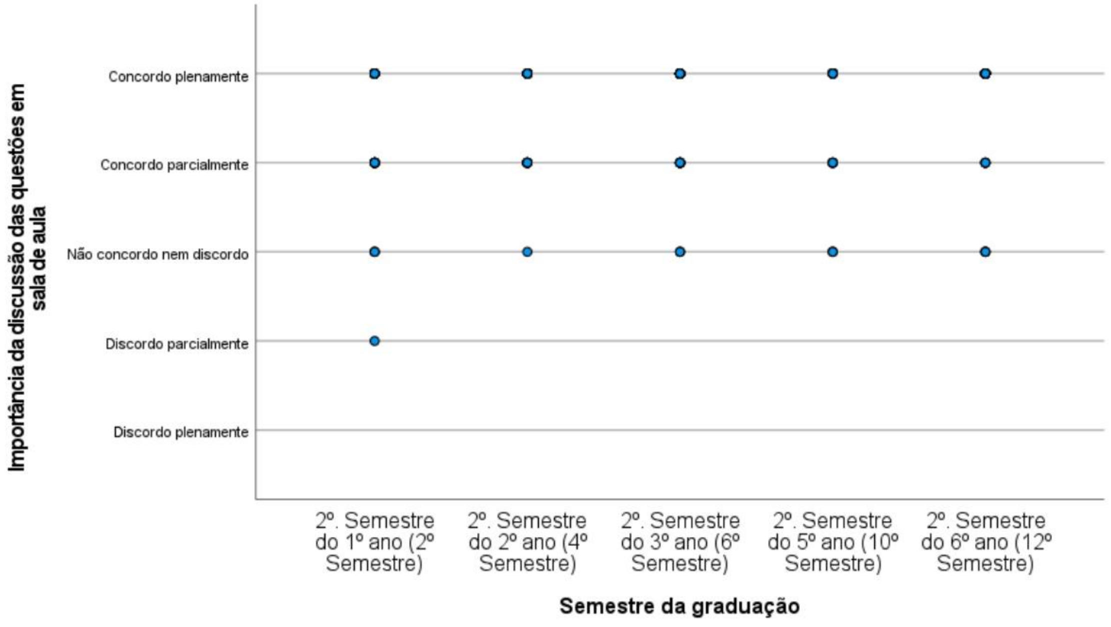


**FIGURE**

Scatter plot between the item "Importance of discussing issues in the classroom" and undergraduate semester.

Machine Translated by Google

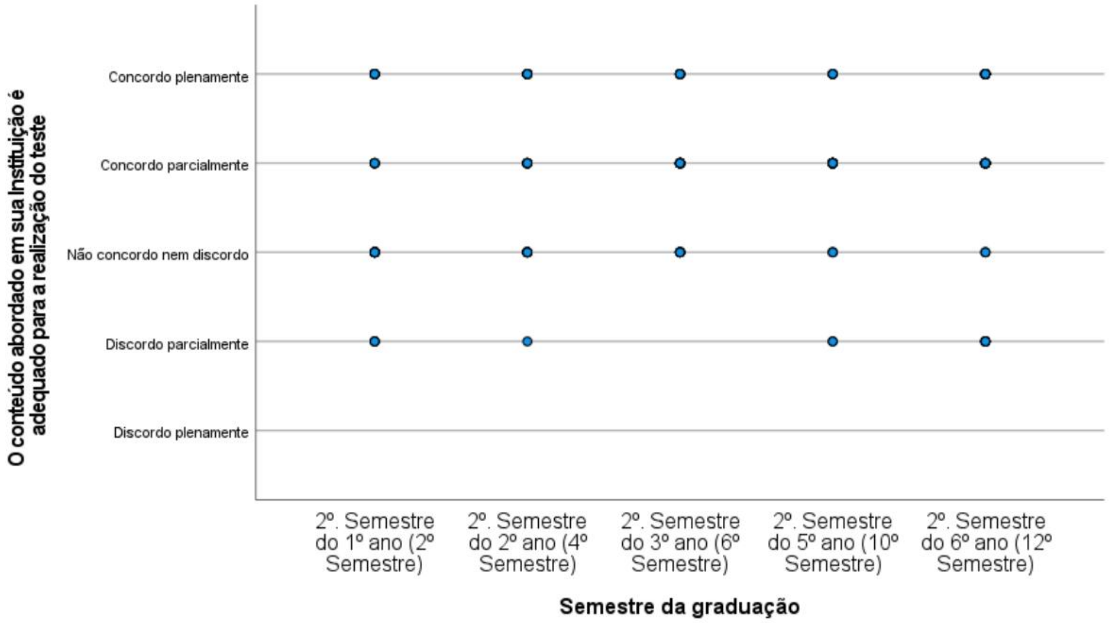


**FIGURE**

Scatter plot between the item "The content covered in your Institution is suitable for taking the test" and undergraduate semester.

Machine Translated by Google

In the Table below, we can see that there was no correlation between the responses regarding motivation and use of TP results by the student himself for his own development academic year and the undergraduate semester. Therefore, the student's perception of these questions was independent of the semester attended. This is illustrated in the respective scatter plots.

Table

Correlation between the undergraduate semester and the responses regarding motivation and use of the TP results by the student himself for his academic development*.

| Item | Graduation series | |
|---|---|---|
| | lol | IC (95%) |
| Motivated to take the test | 0.147 | 0.005;0.283 |
| Importance of taking the test for academic development | -0.001 | -0.142; 0.140 |
| Takes into account development on the test to assess academic development | 0.093 | -0.068; 0.249 |
| Takes into account the evolution of test performance to guide studies | 0.075 | -0.085; 0.232 |

rs = Spearman correlation coefficient; CI, confidence interval.

*The responses to the instrument's questions were reported using a Likert-type scale, where: 0 = I completely disagree; 1 = I partially disagree; 2 = I neither agree nor disagree; 3 = I partially agree; 4 = I completely agree.

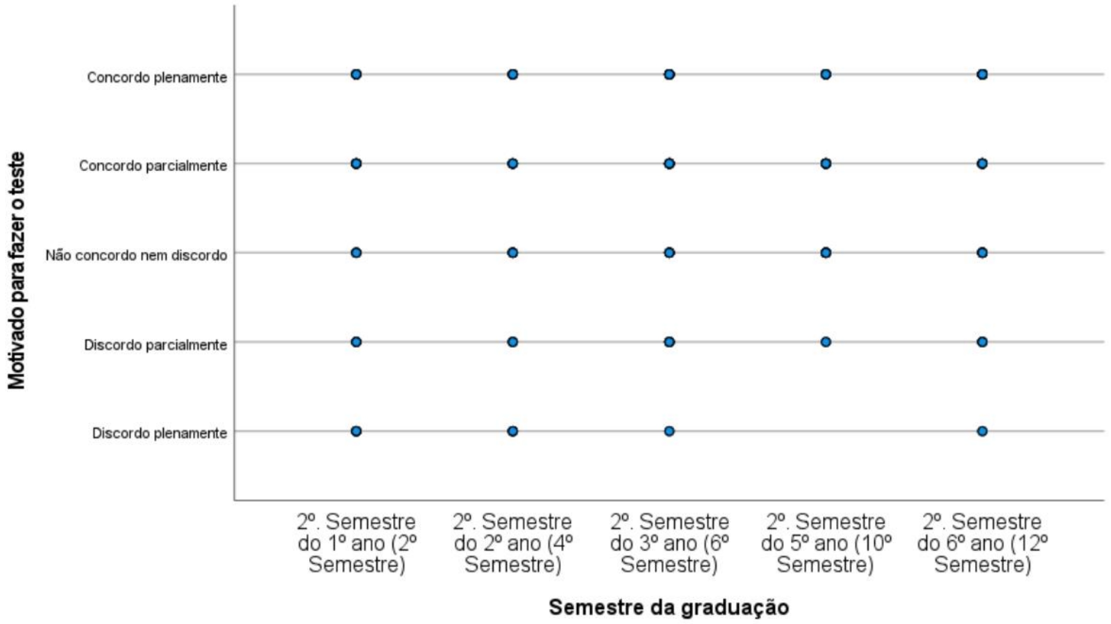


**FIGURE**

Scatter plot between the item "Motivated to take the test" and semester of graduation.

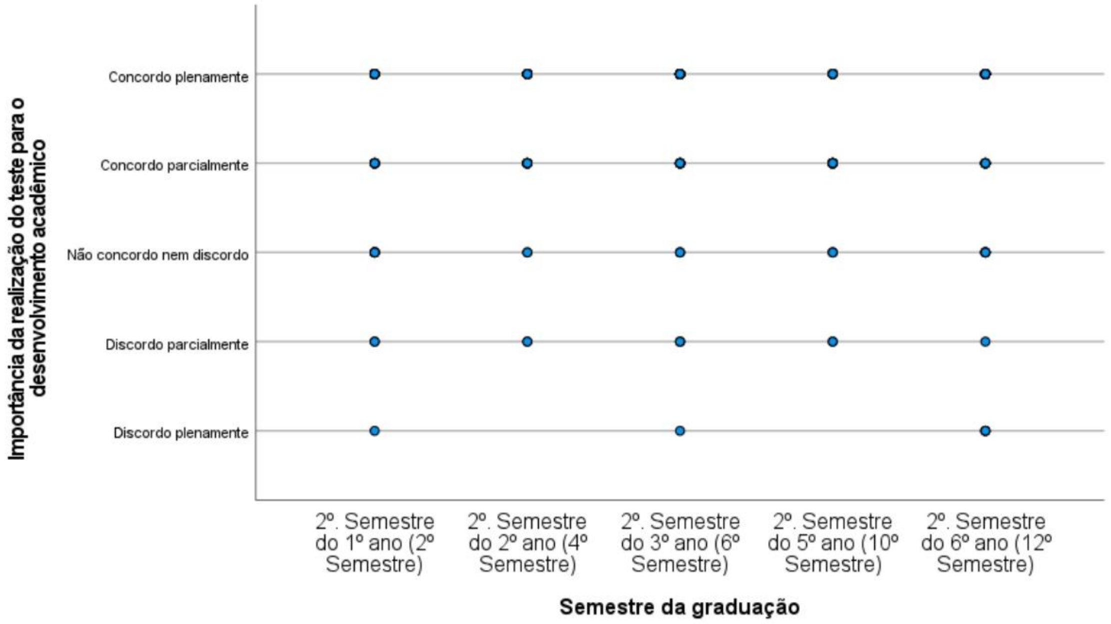


**FIGURE**

Scatter plot between the item "Importance of taking the test for academic development" and undergraduate semester.

Machine Translated by Google

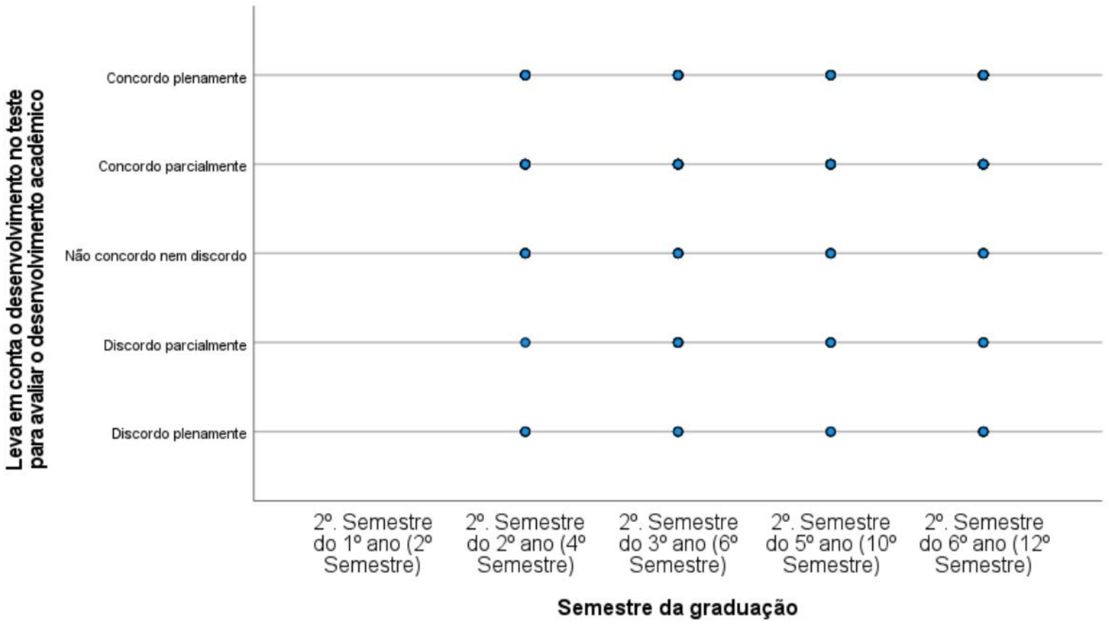


**FIGURE**

Scatter plot between the item “Takes into account development in the test to assess academic development” and undergraduate semester.

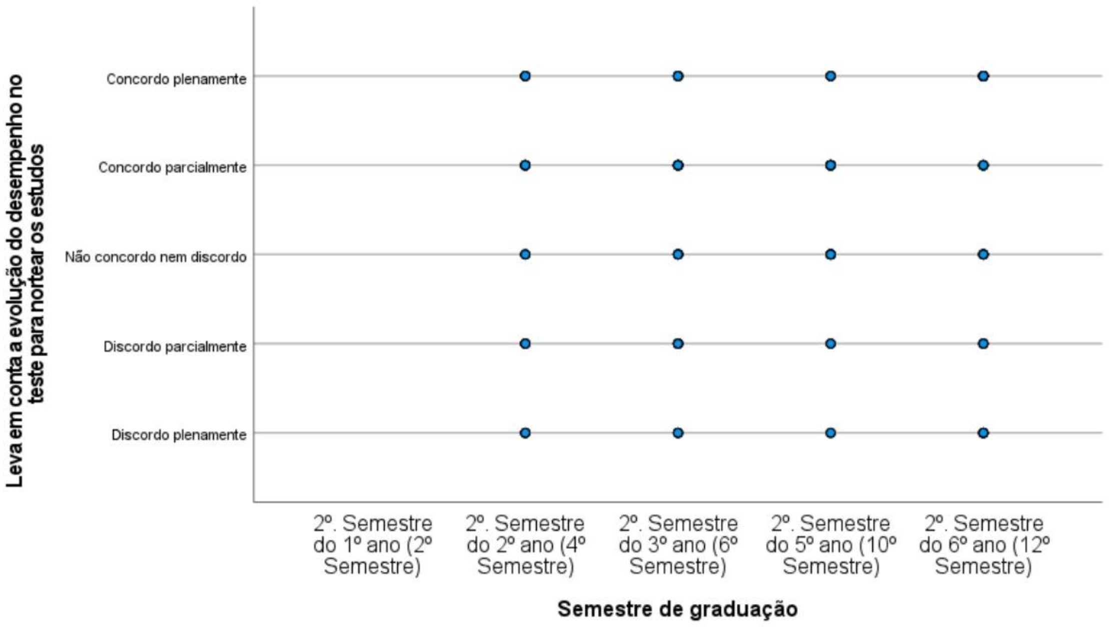


**FIGURE**

Scatter plot between the item “Takes into account the evolution of test performance to guide studies” and the semester of graduation.

Machine Translated by Google

**ANALYSIS 2: Comparative analysis between the two HEIs**

**4. Descriptive analysis of the sample**

Table

Age range and year of graduation of students from both HEIs.

| | IES | |
|---|---|---|
| | UNISA | FAMERP |
| Variable | N = 709 | N = 220 |
| Age, n (%) | | |
| 17 to 20 years old | 263 (37.1) | 32 (14.5) |
| 21 to 25 years old | 350 (49.3) | 131 (59.5) |
| 26 to 30 years old | 67 (9.4) | 38 (17.3) |
| 31 to 35 years old | 16 (2.3) | 5 (2.3) |
| 36 to 39 years old | 4 (0.6) | 0 (0.0) |
| Over 40 years old | 9 (1.3) | 1 (0.5) |
| Did not respond | 0 (0.0) | 13 (5.9) |
| Graduation year, n (%) | | |
| 1st Year | 260(36.7) | 46 (20.9) |
| 2nd Year | 148(20.9) | 32 (14.5) |
| 3rd Year | 99(14) | 42 (19.1) |
| 4th Year | 67(9.4) | 0 (0.0) |
| 5th Year | 61(8.6) | 37 (16.8) |
| 6th Year | 74(10.4) | 50 (22.8) |
| Did not respond | 0 (0.0) | 13 (5.9) |

Categorical variables are described in number (percentage); 1st Year = 1st semester and 2nd semester; 2nd Year = 3rd semester and 4th semester; 3rd Year = 5th semester and 6th semester; 4th Year = 7th semester and 8th semester; 5th Year = 9th semester and 10th semester; 6th Year = 11th semester and 12th.

Semester; IES, higher education institution.

Machine Translated by Google

Table

Distribution of responses to the item "Percentage of questions you expect to get right", according to the year of graduation attended by students at the two HEIs.

| Graduation year | Percentage of questions you expect to get right | IES | |
|---|---|---|---|
| | | UNISA | FAMERP |
| 1st year | | | |
| | 0 to 20% | 157 (60.4) | 31/46 (67.4) |
| | 20 to 40% | 86 (33.1) | 14/46 (30.4) |
| | 40 to 60% | 10 (3.8) | 0/46 (0.0) |
| | 60 to 80% | 2 (0.8) | 0/46 (0.0) |
| | 80 to 100% | 5 (1.9) | 1/46 (2.2) |
| 2nd year | | | |
| | 0 to 20% | 28 (18.9) | 1/32 (3.1) |
| | 20 to 40% | 90 (60.8) | 17/32 (53.1) |
| | 40 to 60% | 26 (17.6) | 12/32 (37.5) |
| | 60 to 80% | 3 (2.0) | 2/32 (6.3) |
| | 80 to 100% | 1 (0.7) | 0/32 (0.0) |
| 3rd year | | | |
| | 0 to 20% | 2 (2.0) | 0/42 (0.0) |
| | 20 to 40% | 32 (32.3) | 17/42 (40.5) |
| | 40 to 60% | 48 (48.6) | 21/42 (50) |
| | 60 to 80% | 13 (13.1) | 4/42 (9.5) |
| | 80 to 100% | 4 (4.0) | 0/42 (0.0) |
| 4th year | | | |
| | 20 to 40% | 6 (9.0) | 0 (0.0) |
| | 40 to 60% | 37 (55.2) | 0 (0.0) |
| | 60 to 80% | 23 (34.3) | 0 (0.0) |
| | 80 to 100% | 1 (1.5) | 0 (0.0) |
| 5th year | | | |
| | 0 to 20% | 1 (1.6) | 2/36 (5.6) |
| | 20 to 40% | 1 (1.6) | 0/36 (0.0) |
| | 40 to 60% | 27 (44.3) | 5/36 (13.9) |
| | 60 to 80% | 30 (49.2) | 25/36 (69.4) |
| | 80 to 100% | 2 (3.3) | 4/36 (11.1) |
| 6th year | | | |
| | 0 to 20% | 0 (0.0) | 2/50 (4) |
| | 20 to 40% | 1 (1.4) | 0/50 (0.0) |
| | 40 to 60% | 22 (29.7) | 6/50 (12) |
| | 60 to 80% | 46 (62.1) | 24/50 (48) |
| | 80 to 100% | 5 (6.8) | 18/50 (36) |

1st Year = 1st semester and 2nd semester; 2nd Year = 3rd semester and 4th semester; 3rd Year = 5th semester and 6th semester; 4th Year = 7th semester and 8th semester; 5th Year = 9th semester and 10th semester; 6th Year = 11th semester and 12th semester; HEI, higher education institution.

Machine Translated by Google

## 5. Comparative analysis between HEIs

Table

Comparative analysis of the item “area believed to have better performance” among students from the two HEIs.

| Item | Answers | IES | | Total | P-value |
|---|---|---|---|---|---|
| | | UNISA | FAMERP | | |
| Area that believes have better performance | Basic | 255<br>36.0% | 71<br>34.3% | 326<br>35.6% | 0.117 |
| | Clinic | 127<br>17.9% | 41<br>19.8% | 168<br>18.3% | |
| | Surgery | 74<br>10.4% | 30<br>14.5% | 104<br>11.4% | |
| | GO | 78<br>11.0% | 11<br>5.3% | 89<br>9.7% | |
| | Pediatrics | 41<br>5.8% | 10<br>4.8% | 51<br>5.6% | |
| | Collective health | 134<br>18.9% | 44<br>21.3% | 178<br>19.4% | |
| | Total | 709<br>100.0% | 207<br>100.0% | 916<br>100.0% | |

Pearson's Chi-square test; HEI, higher education institution.

Machine Translated by Google

Table

Comparative analysis of items on TP adequacy and possible institutional movements to promote academic adherence to TP among students at both HEIs.

| Item | Answers | IES | | | P-value |
|---|---|---|---|---|---|
| | | UNISA | FAMERP | Total | |
| The questions and alternatives are clear to answer. | I completely disagree | 2<br>0.3% | 0<br>0.0% | 2<br>0.2% | 0.101 |
| | I partially disagree | 22<br>3.1% | 9<br>4.3% | 31<br>3.4% | |
| | I neither agree nor disagree | 90<br>12.8% | 17<br>8.2% | 107<br>11.8% | |
| | I partially agree | 248<br>35.3% | 9<br>43.5% | 338<br>37.1% | |
| | I completely agree | 341<br>48.5% | 91<br>44.0% | 432<br>47.5% | |
| | Total | 703<br>100.0% | 207<br>100.0% | 910<br>100.0% | |
| Completion time is appropriate for the content | I completely disagree | 23<br>3.3% | 11<br>5.3% | 34<br>3.7% | 0.689 |
| | I partially disagree | 51<br>7.2% | 14<br>6.8% | 65<br>7.1% | |
| | I neither agree nor disagree | 78<br>11.0% | 21<br>10.1% | 99<br>10.8% | |
| | I partially agree | 163<br>23.1% | 44<br>21.3% | 207<br>22.6% | |
| | I completely agree | 392<br>55.4% | 117<br>56.5% | 509<br>55.7% | |
| | Total | 707<br>100.0% | 207<br>100.0% | 914<br>100.0% | |
| Received prior information from the Institution about the importance of the test | I completely disagree | 14<br>2.0% | 1<br>0.5% | 15<br>1.6% | 0.621 |
| | I partially disagree | 27<br>3.8% | 9<br>4.3% | 36<br>3.9% | |
| | I neither agree nor disagree | 31<br>4.4% | 11<br>5.3% | 42<br>4.6% | |
| | I partially agree | 106<br>15.0% | 30<br>14.5% | 136<br>14.9% | |
| | I completely agree | 530<br>74.9% | 156<br>75.4% | 686<br>75.0% | |
| | Total | 708<br>100.0% | 207<br>100.0% | 915<br>100.0% | |

Pearson's Chi-square test; PT, progress test; HEI, higher education institution.

Machine Translated by Google

Table

Comparative analysis of the items on “intend to access the commented answer sheet” and “intend to access the result” between students from both HEIs.

| Item | Answers | IES | | | P-value |
|---|---|---|---|---|---|
| | | UNISA | FAMERP | Total | |
| Intends access the commented template | I completely disagree | 13<br>1.8% | 3<br>1.4% | 16<br>1.7% | 0.364 |
| | I partially disagree | 19<br>2.7% | 8<br>3.9% | 27<br>3.0% | |
| | I neither agree nor disagree | 59<br>8.3% | 14<br>6.8% | 73<br>8.0% | |
| | I partially agree | 75<br>10.6% | 31<br>15.0% | 106<br>11.6% | |
| | I completely agree | 542<br>76.6% | 151<br>72.9% | 693<br>75.7% | |
| | Total | 708<br>100.0% | 207<br>100.0% | 915<br>100.0% | |
| Intends access the result | I completely disagree | 7<br>1.0% | 2<br>1.0% | 9<br>1.0% | 0.964 |
| | I partially disagree | 9<br>1.3% | 2<br>1.0% | 11<br>1.2% | |
| | I neither agree nor disagree | 40<br>5.6% | 10<br>4.8% | 50<br>5.5% | |
| | I partially agree | 63<br>8.9% | 21<br>10.1% | 84<br>9.2% | |
| | I completely agree | 589<br>83.2% | 172<br>83.1% | 761<br>83.2% | |
| | Total | 708<br>100.0% | 207<br>100.0% | 915<br>100.0% | |

Pearson's Chi-square test; PT, progress test; HEI, higher education institution.

Machine Translated by Google

Below are comparisons regarding the use of TP results by the HEI, from the student's perspective.

As can be seen in the table below, there was a significant difference in the responses to the item “the issues are discussed later in the classroom”. Only the category “partially disagree” was similar between the two HEIs, while all the others presented significant differences, as can be seen by the notation of the letters “a” and “b”. A greater proportion of Famerp students completely disagreed that the issues are discussed later in the classroom, while UNISA students presented a greater proportion of neutral or agreement than Famerp students.

Regarding the item “the content covered at your institution is suitable for taking the test”, there was a difference only for the categories “partially agree” (higher proportion of Famerp students) and “fully agree” (higher proportion of UNISA students).

Table

Comparative analysis of items on the use of TP results by the HEI among students from both HEIs.

| Item | Answers | IES | | | |
|---|---|---|---|---|---|
| | | UNISA | FAMERP | Total | Value P |
| The questions are later discussed in the classroom | I completely disagree | 200a | 128b | 328 | |
| | | 28.3% | 62.4% | 36.0% | |
| | I partially disagree | 105a | 22a | 127 | |
| | | 14.9% | 10.7% | 13.9% | |
| | I neither agree nor disagree | 231a | 47b | 278 | **< 0.001*** |
| | | 32.7% | 22.9% | 30.5% | |
| | I partially agree | 60a | | 62 | |
| | | 8.5% | 2b | 6.8% | |
| | I completely agree | 110a | | 116 | |
| | | 15.6% | 1.0% 6b 2.9% | 12.7% | |
| | Total | 706 | 205 | 911 | |
| | | 100.0% | 100.0% | 100.0% | |
| Importance of discussing issues in the classroom | I completely disagree | 10 | 0 | 10 | |
| | | 1.4% | 0.0% | 1.1% | |
| | I partially disagree | 16 | 1 | 17 | |
| | | 2.3% | 0.5% | 1.9% | |
| | I neither agree nor disagree | 48 | 14 | 62 | 0.051† |
| | | 6.8% | 6.8% | 6.8% | |
| | I partially agree | 125 | 50 | 175 | |
| | | 17.7% | 24.3% | 19.2% | |
| | I completely agree | 507 | 141 | 648 | |
| | | 71.8% | 68.4% | 71.1% | |
| | Total | 706 | 206 | 912 | |

| | | | | | |
|---|---|---|---|---|---|
| | | 100.0% | 100.0% | 100.0% | |
| The content covered in your Institution is suitable for conducting the test | I completely disagree | 8th | 0a | 8 | < 0.001* |
| | | 1.1% | 0.0% | 0.9% | |
| | I partially disagree | 49a | 13a | 62 | |
| | | 7.0% | 6.3% | 6.8% | |
| | I neither agree nor disagree | 122a | 45a | 167 | |
| | | 17.3% | 21.8% | 18.4% | |
| | I partially agree | 230a | 94b | 324 | |
| | | 32.7% | 45.6% | 35.6% | |
| | I completely agree | 295a | 54b | 349 | |
| | | 41.9% | 26.2% | 38.4% | |
| | Total | 704 | 206 | 910 | |
| | | 100.0% | 100.0% | 100.0% | |

*Pearson's chi-square test; †Fisher's exact test; PT, progress test; HEI, higher education institution.

Machine Translated by Google

Below, comparisons are shown for the items on motivation and use of TP results by the student himself for his academic development, between students from the two HEIs.

As can be seen in the tables below, there was a significant difference for 3 items.

For the item "motivated to take the test", there was a difference in the categories "partially disagree", with a greater proportion of Famerp than UNISA students, neutral position "neither agree nor disagree" also greater for Famerp students and "fully agree" greater proportion of UNISA students.

For the item "importance of taking the test for academic development", the categories "partially disagree" and "partially agree" had a higher proportion of Famerp students; on the other hand, the category "fully agree" was chosen by the majority of UNISA students (71.8%), with a significant difference in relation to Famerp students (51%).

For the item "takes into account test performance to guide studies", the "partially disagree" category had a higher proportion for Famerp students, while the "fully agree" category had a higher proportion for UNISA students.

Table

Comparative analysis of items on motivation and use of TP results by the student himself for his academic development among students from the two HEIs.

| Item | Answers | IES | | | P-value |
|---|---|---|---|---|---|
| | | UNISA | FAMERP | Total | |
| Motivated to take the test | I completely disagree | | | 57 | < 0.001 |
| | | 41st 5.8% | 16th 7.8% | 6.3% | |
| | I partially disagree | 37a | 25b | 62 | |
| | | 5.3% | 12.1% | 6.8% | |
| | I neither agree nor disagree | 82a | 40b | 122 | |
| | | 11.7% | 19.4% | 13.4% | |
| | I partially agree | 179a | 58a | 237 | |
| | | 25.5% | 28.2% | 26.1% | |
| | I completely agree | 364a | 67b | 431 | |
| | | 51.8% | 32.5% | 47.4% | |
| | Total | 703 | 206 | 909 | |
| | | 100.0% | 100.0% | 100.0% | |
| Importance of taking the test for academic development | I completely disagree | | | 17 | < 0.001 |
| | | 11th 1.6% | 6th 2.9% | 1.9% | |
| | I partially disagree | 9a | 10b | 19 | |
| | | 1.3% | 4.9% | 2.1% | |

| | | | | | |
|---|---|---|---|---|---|
| | I neither agree nor disagree | 49a | 21st | 70 | |
| | | 7.0% | 10.2% | 7.7% | |
| | I partially agree | 129a | 64b | 193 | |
| | | 18.4% | 31.1% | 21.3% | |
| | I completely agree | 504a | 105b | 609 | |
| | | 71.8% | 51.0% | 67.1% | |
| | Total | 702 | 206 | 908 | |
| | | 100.0% | 100.0% | 100.0% | |
| Takes into account development on the test to assess academic development | I completely disagree | 36 | 19 | 55 | 0.132 |
| | | 8.0% | 11.5% | 8.9% | |
| | I partially disagree | 30 | 18 | 48 | |
| | | 6.6% | 10.9% | 7.8% | |
| | I neither agree nor disagree | 64 | 25 | 89 | |
| | | 14.2% | 15.2% | 14.4% | |
| | I partially agree | 140 | 51 | 191 | |
| | | 31.0% | 30.9% | 31.0% | |
| | I completely agree | 182 | 52 | 234 | |
| | | 40.3% | 31.5% | 37.9% | |
| | Total | 452 | 165 | 617 | |
| | | 100.0% | 100.0% | 100.0% | |
| Takes into account the evolution of test performance to guide studies | I completely disagree | 49a | 24a | 73 | **0.001** |
| | | 10.9% | 14.5% | 11.9% | |
| | I partially disagree | 31st | 25b | 56 | |
| | | 6.9% | 15.2% | 9.1% | |
| | I neither agree nor disagree | 82a | 31st | 113 | |
| | | 18.2% | 18.8% | 18.3% | |
| | I partially agree | 123a | 48a | 171 | |
| | | 27.3% | 29.1% | 27.8% | |
| | I completely agree | 166a | 37b | 203 | |
| | | 36.8% | 22.4% | 33.0% | |
| | Total | 451 | 165 | 616 | |
| | | 100.0% | 100.0% | 100.0% | |

Pearson's Chi-square test; PT, progress test; HEI, higher education institution.

Machine Translated by Google

## 6. References


1. BISHARA, A. J; HITTNER JB **Confidence intervals for correlations when data are not normal.** Behav Res 49, 294–309, 2017.

2. BONETT, D. G; WRIGHT, TA **Sample size requirements for estimating Pearson, Kendall and Spearman correlations.** Psychometrika, 65, 23–28, 2000.

3. BUSSAB, WO; MORETTIN, PA **Basic Statistics.** Saraiva, 9th edition., São Paulo, 2017.
4. CONOVER, WJ **Practical nonparametric statistics.** New York: John Wiley & Sons, 1999.
5. FIELD, A. **Discovering Statistics using SPSS.** Translation, consultancy and supervision by Lorí Viali. Artmed Publishing, 2009.
6. IBM Corporation. **IBM SPSS Statistics Algorithms,** 27th edition. IBM Corp.: Armonk, NY, USA, 2020.

7. MACDONALD, PL; GARDNER, RC **Type I error rate comparisons of post hoc procedures for I j Chi-Square tables.** Educational and psychological measurement, 60(5), 735-754, 2000.
8. R CORE TEAM. A: **A language and environment for statistical computing.** R Foundation for Statistical Computing, Vienna, Austria, 2015. URL https://www.R-project.org/
9. SIEGEL S.; CASTELLAN Jr NJ. **Nonparametric Statistics for Behavioral Sciences.** Bookman, 2nd edition, São Paulo, 2006.
